# Global Existence and Fixed-Time Synchronization of a Hyperchaotic Financial System Governed by Semi-Linear Parabolic Partial Differential Equations Equipped with the Homogeneous Neumann Boundary Condition

**DOI:** 10.3390/e25020359

**Published:** 2023-02-15

**Authors:** Chengqiang Wang, Xiangqing Zhao, Yulin Zhang, Zhiwei Lv

**Affiliations:** 1 School of Mathematics, Suqian University, Suqian 223800, China; 2 School of Mathematics, Chengdu Normal University, Chengdu 611130, China

**Keywords:** hyperchaotic systems, financial systems, well-posedness, fixed-time synchronization, modified energy functionals

## Abstract

Chaotic nonlinear dynamical systems, in which the generated time series exhibit high entropy values, have been extensively used and play essential roles in tracking accurately the complex fluctuations of the real-world financial markets. We are concerned with a system of semi-linear parabolic partial differential equations supplemented by the homogeneous Neumann boundary condition, which governs a financial system comprising the labor force, the stock, the money, and the production sub-blocks distributed in a certain line segment or planar region. The system derived by removing the terms involved with partial derivatives with respect to space variables from our concerned system was demonstrated to be hyperchaotic. We firstly prove, via Galerkin’s method and establishing a priori inequalities, that the initial-boundary value problem for the concerned partial differential equations is globally well posed in Hadamard’s sense. Secondly, we design controls for the response system to our concerned financial system, prove under some additional conditions that our concerned system and its controlled response system achieve drive-response fixed-time synchronization, and provide an estimate on the settling time. Several modified energy functionals (i.e., Lyapunov functionals) are constructed to demonstrate the global well-posedness and the fixed-time synchronizability. Finally, we perform several numerical simulations to validate our synchronization theoretical results.

## 1. Introduction

In recent years, due to the frequent application of advanced economic and/or financial tools in dealing with problems from real financial markets or social governance, it is increasingly interesting to construct nonlinear dynamical systems, which could induce (hyper) chaotic behavior, to study in-depth the economic and/or financial behaviors from the mathematical viewpoint; see [1,2,3,4,5,6,7] for instance, among the vast related references. Huang and Li ([1], pp. 55–60) studied, in the 1990s, the financial scenario that the interest rate is sufficiently influenced only by the investment demand and the price index, and introduced the following 3D chaotic financial model which comprises the labor force sub-block, the stock sub-block, the money sub-block, and the production sub-block:

(1) $$\left\{\begin{array}{cccc} {\dot{v}}_{1}= & \left(v_{2}-a\right)v_{1}+v_{3}\; & \; & \mathrm{in}\;R^{+},\\ {\dot{v}}_{2}= & -bv_{2}-{\left(v_{1}\right)}^{2}+1 & \; & \mathrm{in}\;R^{+},\\ {\dot{v}}_{3}= & -cv_{3}-v_{1} & \; & \mathrm{in}\;R^{+}. \end{array}\right.$$

Here and hereafter: the unknowns $v_{1}$, $v_{2}$, and $v_{3}$ are written for the interest rate, the investment demand, and the price index, respectively; $\dot{v}$ denotes the derivative of the unknown *v* with respect to the time variable *t*, that is, $\dot{v}=\frac{dv}{dt}$; the parameters *a*, *b*, *c*, required to be non-negative, represents the savings, the cost per investment, and the elasticity of demand of commercial markets, respectively; $R^{+}=\left[0,+\infty \right)$; see ([1], pp. 55–60) for details. It is not difficult to find from model (1) that the surplus $v_{2}-a$ between investment and savings contributes positively to the interest rate in a proportional way, and the price index adjusts the interest rate structurally; the cost of investment and the interest rate could reduce the invest demand; the (nominal and/or real) interest rate can influence the inflation rate (and equivalently, the price index). Yu, Cai, and Li [5] considered another financial scenario in which the interest rate is influenced dramatically not only by the investment demand and the price index, but also by the average profit margin, and modified the financial system (1) into the following new financial system:

(2) $$\left\{\begin{array}{cccc} {\dot{v}}_{1}= & \left(v_{2}-a\right)v_{1}+v_{3}+v_{4} & \; & \mathrm{in}\;R^{+},\\ {\dot{v}}_{2}= & -bv_{2}-{\left(v_{1}\right)}^{2}+1 & \; & \mathrm{in}\;R^{+},\\ {\dot{v}}_{3}= & -cv_{3}-v_{1} & \; & \mathrm{in}\;R^{+},\\ {\dot{v}}_{4}= & -\alpha v_{4}-\beta v_{1}v_{2} & \; & \mathrm{in}\;R^{+}, \end{array}\right.$$

where the unknown $v_{4}$ is written for the average profit margin (see (1) for the financial explanation of the unknowns $v_{k}$, k=1,2,3), and the parameters α and β, required to be non-negative, represent the intrinsic restriction on the financial development and the intensity of cost due to investment and the increase in interest rate, respectively. Interested readers can consult [1,2,3,4,5,6] for a more detailed explanation of the unknowns $v_{k}$ (k=1,2,3,4) and the parameters *a*, *b*, *c*, α and β.

As can be seen clearly from models (1) and (2), the regional disparities of the economy have not been taken into consideration in the existing references yet. Since different subregions have different resource advantages and different development strategies and policies, economic development exhibits inevitably regional disparities. For example, regional inequalities of income and wealth exist commonly in eastern and western cities in China. In the meantime, every measure taken to deal with problems in the financial system would necessarily influence the economic development in the future, and therefore, the memory effect that exists in dynamical systems concerning economic evolution should not be neglected. However, as remarked in Reference [8] (see also References [4,9,10]), the memory effect in dynamical systems driven by financial behaviors could result in dissipation. In view of these two phenomena, it seems to be more natural to construct systems of parabolic partial differential equations, whose space variables run over a line segment or a planar region, to track the complex financial behaviors in the real-world markets.

Let Ω be a bounded connected open subset of the *N*-dimensional Euclidean space $R^{N}$ (N∈N, the set of positive integers). In the sequel, we shall always assume that Ω has a $C^{4}$ boundary, and consider our economic development in the region Ω. We shall write hereafter ∇ and div for the gradient and the divergence operator on $R^{N}$, respectively, namely $\nabla ={\left(\partial_{x_{1}},\ldots ,\partial_{x_{N}}\right)}^{⊤}$ and $\mathrm{div}=\sum_{\ell =1}^{N}\partial_{x_{\ell }}$, where $\partial_{x_{\ell }}=\frac{\partial }{\partial x_{\ell }}$ denotes the partial derivative with respect to the space variable $x_{\ell }$, ℓ=1,…,N. In the meantime, we shall denote by ν(x) the outward unit normal vector field along the boundary ∂Ω of the region Ω. As alluded above, memory effect and regional inequalities in economic development in the whole region Ω shall not be neglected in the rest of the paper. To facilitate our explanation here, we write here temporarily v=v(x,t), $\left(x,t\right)\in \Omega \times R^{+}$, for a certain economic quantity occupying the region Ω and evolving in time *t*. Assume that the diffusion of v(x,t) due to regional disparities and/or memory effect can be formulated as div(D(x)∇v(x,t)), where the space-varying coefficient function D(x) is included to stress that the diffusion of the economic quantity depends on the space variables. Following these ideas and the results in References [5,11], we are motivated to consider the new model

(3) $$\left\{\begin{array}{ccc} \partial_{t}v_{1}=\mathrm{div}\left(D_{1}\nabla v_{1}\right)+\left(v_{2}-a\right)v_{1}+v_{3}+v_{4} & \; & \mathrm{in}\;\Omega \times R^{+},\\ \partial_{t}v_{2}=\mathrm{div}\left(D_{2}\nabla v_{2}\right)-bv_{2}-{\left(v_{1}\right)}^{2}+1 & \; & \mathrm{in}\;\Omega \times R^{+},\\ \partial_{t}v_{3}=\mathrm{div}\left(D_{3}\nabla v_{3}\right)-cv_{3}-v_{1} & \; & \mathrm{in}\;\Omega \times R^{+},\\ \partial_{t}v_{4}=\mathrm{div}\left(D_{4}\nabla v_{4}\right)-\alpha v_{4}-\beta v_{1}v_{2} & \; & \mathrm{in}\;\Omega \times R^{+},\\ \partial_{\nu }v_{1}=\partial_{\nu }v_{2}=\partial_{\nu }v_{3}=\partial_{\nu }v_{4}=0 & \; & \mathrm{on}\;\partial \Omega \times R^{+}, \end{array}\right.$$

where $v_{k}=v_{k}\left(x,t\right)$ is the unknown, $D_{k}=D_{k}\left(x\right)$ represents the intensity of the diffusion of $v_{k}$, $\partial_{\nu }v_{k}$ denotes the normal directional derivative of $v_{k}$ (namely $\partial_{\nu }v_{k}=\frac{\partial v_{k}}{\partial \nu }=\nu^{⊤}\nabla v_{k}$), and $\partial_{\nu }v_{k}=0$ means that there is no flux of $v_{k}$ along the boundary of Ω, k=1,2,3,4. The no-flux of $v_{k}$ (k=1,2,3,4) on the boundary means that we are focused on the financial behaviors in Ω and all the ‘external effects’ on $v_{k}$ (k=1,2,3,4) can be neglected. The interested readers could consult [9,12,13,14] for more discussions on motivations to introduce diffusion terms to the financial system (1).

**Remark** **1.** *It is not difficult to find that all the equilibrium states $P_{k}$ (k=1,2,3) (see (4)) of the financial system (2) are indeed equilibrium states of the system (3). This is the very preliminary result of the system (3) from the viewpoint of dynamical system theory.*

**Remark** **2.** *From the mathematical point of view, it seems to be unnecessary to demand that Ω be connected. From the practical viewpoint of finance, if the concerned region is not connected, then we can study the economic behavior separately in each connected component of the whole region. The concerned Ω is usually very large in size; in this situation, the economic development is not sensitive to changes in relatively small portions of the boundary of Ω, and therefore it is natural to assume that Ω has a $C^{4}$ boundary. Besides, the assumption that Ω has its boundary in the class $C^{4}$ would facilitate our later mathematical presentation.*

In the literature, the financial systems (1) and (2) have been widely studied from the point of view of dynamical system theory; see References [3,4,5,6,15,16,17]. We are inspired by these results, and therefore, we are glad to use several lines to recall the related results in these references. It is observed by authors of Reference [5] that when

$$\frac{\alpha b+c\beta +abc\alpha -c\alpha }{c(\beta -\alpha )}>0,$$

the system (2) has three equilibrium states, that is,

(4) $$\begin{array}{c} P_{1}\left(\sqrt{\frac{\alpha b(1+ac)}{c(\beta -\alpha )}+1},\frac{\alpha (1+ac)}{c(\beta -\alpha )},-\frac{1}{c}\sqrt{\frac{\alpha b(1+ac)}{c(\beta -\alpha )}+1},-\frac{\beta (1+ac)}{c(\beta -\alpha )}\sqrt{\frac{\alpha b(1+ac)}{c(\beta -\alpha )}+1}\right),\\ P_{2}\left(0,\frac{1}{b},0,0\right),\;\mathrm{and}\\ P_{3}\left(-\sqrt{\frac{\alpha b(1+ac)}{c(\beta -\alpha )}+1},\frac{\alpha (1+ac)}{c(\beta -\alpha )},\frac{1}{c}\sqrt{\frac{\alpha b(1+ac)}{c(\beta -\alpha )}+1},-\frac{\beta (1+ac)}{c(\beta -\alpha )}\sqrt{\frac{\alpha b(1+ac)}{c(\beta -\alpha )}+1}\right). \end{array}$$

When a=0.9, b=0.2, c=1.5, α=0.17, and β=0.2, it is concluded in Reference [5] that $P_{k}$ is an unstable saddle point of the dynamical system (2), k=1,2,3, and that the dynamical system (2) has four Lyapunov exponents: −1.1499, 0, 0.018041, 0.034432. These numerical simulations, together with some other calculations, imply that system (2) is indeed hyperchaotic. As explained and visualized in Reference [18], the entropy of time series generated by the financial system (2) exhibits relatively high values when system (2) undergoes the hyperchaos phenomenon; see References [18,19,20,21,22], for instance, for more explanation on the relation between entropy and chaos. This could help us locate accurately chaotic and periodic attractors in the system (2). Chaos (or hyperchaos) in financial systems brings in difficulties in predictions and financial systems planning; see References [2,23].

To provide scientific suggestions in management and decision-making strategies to maintain the saving amount to a certain level that restores the economic cycle’s normalization, it is necessary to study, via mathematical tools, the possibility of coming up with suitable controls to suppress (stabilize) or synchronize the (hyper)chaos; see Reference [24]. For control systems, stabilization and synchronization have close relationships. The stabilization problem has already been extensively investigated for (hyper)chaotic financial systems; the interested readers can consult, for instance, References [13,14,25,26,27], for some intuition and new observations concerning this topic. Analogously to the stabilization problem, the synchronization problem for (hyper)chaotic financial systems has also been investigated in several references; see References [28,29,30,31,32,33]. Synchronization is one of the most interesting collective behaviors of dynamical systems, and therefore has aroused tremendous interest in many application fields, such as secure communication, biological systems, and information processing; see References [26,34,35,36,37] and the references cited therein.

As will be shown graphically, the financial system (3) is ‘chaotic’; see Section 4. One of our aims in this paper is to study the synchronization problem for system (3). To formulate clearly our synchronization problem, we have to introduce the response system

(5) $$\left\{\begin{array}{ccc} \partial_{t}{\tilde{v}}_{1}=\mathrm{div}\left(D_{1}\nabla {\tilde{v}}_{1}\right)+\left({\tilde{v}}_{2}-a\right){\tilde{v}}_{1}+{\tilde{v}}_{3}+{\tilde{v}}_{4}+W_{1} & \; & \mathrm{in}\;\Omega \times R^{+},\\ \partial_{t}{\tilde{v}}_{2}=\mathrm{div}\left(D_{2}\nabla {\tilde{v}}_{2}\right)-b{\tilde{v}}_{2}-{\left({\tilde{v}}_{1}\right)}^{2}+1+W_{2} & \; & \mathrm{in}\;\Omega \times R^{+},\\ \partial_{t}{\tilde{v}}_{3}=\mathrm{div}\left(D_{3}\nabla {\tilde{v}}_{3}\right)-c{\tilde{v}}_{3}-{\tilde{v}}_{1}+W_{3} & \; & \mathrm{in}\;\Omega \times R^{+},\\ \partial_{t}{\tilde{v}}_{4}=\mathrm{div}\left(D_{4}\nabla {\tilde{v}}_{4}\right)-\alpha {\tilde{v}}_{4}-\beta {\tilde{v}}_{1}{\tilde{v}}_{2}+W_{4} & \; & \mathrm{in}\;\Omega \times R^{+},\\ \partial_{\nu }{\tilde{v}}_{1}=\partial_{\nu }{\tilde{v}}_{2}=\partial_{\nu }{\tilde{v}}_{3}=\partial_{\nu }{\tilde{v}}_{4}=0 & \; & \mathrm{on}\;\partial \Omega \times R^{+}, \end{array}\right.$$

in which the unknown ${\tilde{v}}_{k}={\tilde{v}}_{k}\left(x,t\right)$ has the same meaning as $v_{k}$ in system (3), and $W_{k}=W_{k}\left(x,t\right)$ denotes a control input, k=1,2,3,4. For system (5), (3) is called the drive system. Additionally, our synchronization problem is closely related to the following definition.

**Definition** **1.** *The drive system (3) and the response system (5) are said to achieve fixed-time synchronization, or be synchronized in a fixed time, provided that there exists a positive time instant T (usually referred to as the settling time) and a control quadruple ${\left(W_{1},W_{2},W_{3},W_{4}\right)}^{⊤}$, such that for every trajectory quadruple ${\left(v_{1},v_{2},v_{3},v_{4}\right)}^{⊤}$ of system (3) and every trajectory quadruple ${\left({\tilde{v}}_{1},{\tilde{v}}_{2},{\tilde{v}}_{3},{\tilde{v}}_{4}\right)}^{⊤}$ of system (5) with the control ${\left(W_{1},W_{2},W_{3},W_{4}\right)}^{⊤}$ implemented, we have*

(6) $$\begin{array}{cccccc} \; & {\tilde{v}}_{k}={\tilde{v}}_{k} & \; & a.e.\;\mathrm{in}\;\Omega \times [T,+\infty ), & \; & k=1,2,3,4. \end{array}$$

**Remark** **3.** *The sense in which a quadruple ${\left(v_{1},v_{2},v_{3},v_{4}\right)}^{⊤}$ is a trajectory of system (3) and a quadruple ${\left({\tilde{v}}_{1},{\tilde{v}}_{2},{\tilde{v}}_{3},{\tilde{v}}_{4}\right)}^{⊤}$ is a trajectory of system (5) will be given as in Definition 3. As will be illuminated by Theorems 2 and 4, we shall give ${\left(W_{1},W_{2},W_{3},W_{4}\right)}^{⊤}$ as in (48), and prove that both the trajectory of system (3) and the trajectory of the controlled system (5) exist globally in time and belong to the space $C(R^{+};L^{2}\left(\Omega ;R^{4}\right))$. This ensures that (6) makes sense and implies that Definition 1 is indeed equivalent to (58), and also equivalent to (6) along with*

$$\begin{array}{cc} \; & {\left({\tilde{v}}_{1}\left(\cdot ,t\right)-v_{1}\left(\cdot ,t\right),{\tilde{v}}_{2}\left(\cdot ,t\right)-v_{2}\left(\cdot ,t\right),{\tilde{v}}_{3}\left(\cdot ,t\right)-v_{3}\left(\cdot ,t\right),{\tilde{v}}_{4}\left(\cdot ,t\right)-v_{4}\left(\cdot ,t\right)\right)}^{⊤}\\ \to & {\left({\tilde{v}}_{1}\left(\cdot ,T\right)-v_{1}\left(\cdot ,T\right),{\tilde{v}}_{2}\left(\cdot ,T\right)-v_{2}\left(\cdot ,T\right),{\tilde{v}}_{3}\left(\cdot ,T\right)-v_{3}\left(\cdot ,T\right),{\tilde{v}}_{4}\left(\cdot ,T\right)-v_{4}\left(\cdot ,T\right)\right)}^{⊤}\\ = & 0\;\mathrm{in}\;L^{2}\left(\Omega ;R^{4}\right),\;\mathrm{as}\;t\to T. \end{array}$$

*Due to these equivalences, to establish fixed-time synchronizability, it suffices to prove (58).*

As pointed out above, several papers have already been published concerning the synchronization problem for (hyper)chaotic financial systems. Among them, as will be seen later, are the results in References [30,31,32], which are closer to the synchronization result in this paper. Yousefpour et al. [30] designed an adaptive terminal sliding mode control, equipped with a radial basis function neural network estimator, for the response system to the time-fractional-order (in the Grünwald–Letnikov sense) counterpart of the financial system (2), and came up with a criterion to guarantee that the the time-fractional-order (in the Grünwald-Letnikov sense) counterpart of (2) and its response system with the designed control implemented achieves finite-time synchronization. The idea of using a neural network estimator in designing synchronization control was also applied to the financial system (1). Yao et al. [31] proposed a suitable control for the response system corresponding to the financial system (1) based on a neural adaptive control approach, and proved, with the aid of a barrier Lyapunov function, that the financial system (1) and its response system with the proposed control implemented achieved fixed-time synchronization. As mentioned in Reference [31], the control designed in Reference [31] has many advantages over the linear feedback control used in several other references; among them, it ensures that the synchronization errors remain always within the predefined output constraints. Almost in the same time period, He, Peng, and Zheng [32] considered the fixed-time synchronization problem for time-fractional-order counterparts of the financial system (1), more precisely, they designed an appropriate control for the response system to the time-fractional-order (in Caputo’s sense) counterpart of the system (1), and provided a criterion to guarantee that the the time-fractional-order (in Caputo’s sense) counterpart of (1) and its response system with the designed control implemented achieved fixed-time synchronization.

Our another aim of this paper comes from the observation: we have to explain in detail what we mean by a trajectory of the financial system (3) or the financial system (5). As is known to all, the definition of trajectories of the financial systems (1) and (2) is very clear, and the justification of the existence of trajectories is very classical in the literature. However, for the financial systems (3) and (5), it is very complicated to define the trajectory, and it is difficult to verify the existence of trajectories. To overcome these difficulties, we start by equipping the financial system (3) with the initial condition

(7) $$\left\{\begin{array}{ccc} v_{1}\left(\cdot ,0\right)=v_{1}^{0} & \; & \mathrm{in}\;\Omega ,\\ v_{2}\left(\cdot ,0\right)=v_{2}^{0} & \; & \mathrm{in}\;\Omega ,\\ v_{3}\left(\cdot ,0\right)=v_{3}^{0} & \; & \mathrm{in}\;\Omega ,\\ v_{4}\left(\cdot ,0\right)=v_{4}^{0} & \; & \mathrm{in}\;\Omega , \end{array}\right.$$

and by equipping the financial system (5) with the initial condition

(8) $$\left\{\begin{array}{ccc} {\tilde{v}}_{1}\left(\cdot ,0\right)={\tilde{v}}_{1}^{0} & \; & \mathrm{in}\;\Omega ,\\ {\tilde{v}}_{2}\left(\cdot ,0\right)={\tilde{v}}_{2}^{0} & \; & \mathrm{in}\;\Omega ,\\ {\tilde{v}}_{3}\left(\cdot ,0\right)={\tilde{v}}_{3}^{0} & \; & \mathrm{in}\;\Omega ,\\ {\tilde{v}}_{4}\left(\cdot ,0\right)={\tilde{v}}_{4}^{0} & \; & \mathrm{in}\;\Omega , \end{array}\right.$$

where $v_{k}^{0}$ and ${\tilde{v}}_{k}^{0}$ are to be given in a certain function space, k=1,2,3,4. Now, we would like to clarify the exact sense of a solution to the Neumann boundary value problem (3) or a trajectory of the model (3). The definition of the trajectory of the financial system (5) can be given in a similar way. We start by recalling the notion of classical solutions to systems of parabolic-type partial differential equations.

**Definition** **2.** *Let T∈(0,+∞). The quadruple*

$${\left(v_{1},v_{2},v_{3},v_{4}\right)}^{⊤}\in C^{2,1}\left(\bar{\Omega }\times \left(0,T\right];R^{4}\right)\cap C\left(\bar{\Omega }\times \left[0,T\right];R^{4}\right)$$

*is said to be a classical solution to the boundary value problem (3), or a trajectory of the financial system (3) in the strict sense, in the interval [0,T], provided that the quadruple ${\left(v_{1},v_{2},v_{3},v_{4}\right)}^{⊤}$ satisfies the partial differential equations in the problem (3), and satisfies the boundary conditions $\partial_{\nu }v_{1}=\partial_{\nu }v_{2}=\partial_{\nu }v_{3}=\partial_{\nu }v_{4}=0$ on ∂Ω×[0,T).*

*Let 0<T⩽+∞. The quadruple ${\left(v_{1},v_{2},v_{3},v_{4}\right)}^{⊤}$ is said to be a classical solution to the boundary value problem (3), or a trajectory of the financial system (3) in the strict sense, in the interval [0,T), provided that the restriction ${\left(v_{1},v_{2},v_{3},v_{4}\right)}^{⊤}{|}_{\Omega \times [0,\tilde{T}]}$, for every $0<\tilde{T}<T$, is a classical solution to the problem (3), or a trajectory of the system (3) in the strict sense, in the interval $\left[0,\tilde{T}\right]$.*

In the real world, the values of ${\left(v_{1},v_{2},v_{3},v_{4}\right)}^{⊤}$ are indeed collected by financial workers or the government. Therefore, the function vector ${\left(v_{1},v_{2},v_{3},v_{4}\right)}^{⊤}$ should have a lower regularity. Thus, we are led to the following definition:

**Definition** **3.** *Given ${\left(v_{1}^{0},v_{2}^{0},v_{3}^{0},v_{4}^{0}\right)}^{⊤}\in L^{2}\left(\Omega ;R^{4}\right)$. Let T∈(0,+∞). The quadruple*

$${\left(v_{1},v_{2},v_{3},v_{4}\right)}^{⊤}\in C\left(\left[0,T\right];L^{2}\left(\Omega ;R^{4}\right)\right)\cap L^{2}\left(0,T;H^{1}\left(\Omega ;R^{4}\right)\right)$$

*is said to be a weak solution to to the initial-boundary value problem (3)–(7), or a trajectory of the financial system (3) satisfying (7), in the interval [0,T], provided that the quadruple ${\left(v_{1},v_{2},v_{3},v_{4}\right)}^{⊤}$ satisfies the following: For every quadruple ${\left(\varphi_{1},\varphi_{2},\varphi_{3},\varphi_{4}\right)}^{⊤}\in H^{1}\left(\Omega ;R^{4}\right)$ of test functions, it holds that*

(9) $$\begin{array}{cc} \; & \int_{\Omega }v_{1}\left(x,t\right)\varphi_{1}\left(x\right)dx-\int_{\Omega }v_{1}^{0}\left(x\right)\varphi_{1}\left(x\right)dx\\ = & \int_{0}^{t}\int_{\Omega }\varphi_{1}\left(x\right)\left(\left(v_{2}\left(x,s\right)-a\right)v_{1}\left(x,s\right)+v_{3}\left(x,s\right)+v_{4}\left(x,s\right)\right)dxds\\ \; & -\int_{0}^{t}\int_{\Omega }D_{1}\left(x\right)\nabla^{⊤}v_{1}\left(x,s\right)\nabla \varphi_{1}\left(x\right)dxds\;\mathrm{for}\;t\in \left[0,T\right],\\ \; & \int_{\Omega }v_{2}\left(x,t\right)\varphi_{2}\left(x\right)dx-\int_{\Omega }v_{2}^{0}\left(x\right)\varphi_{2}\left(x\right)dx\\ = & \int_{0}^{t}\int_{\Omega }\varphi_{2}\left(x\right)\left(1-bv_{2}\left(x,s\right)-{\left(v_{1}\left(x,s\right)\right)}^{2}\right)dxds\\ \; & -\int_{0}^{t}\int_{\Omega }D_{2}\left(x\right)\nabla^{⊤}v_{2}\left(x,s\right)\nabla \varphi_{2}\left(x\right)dxds\;\mathrm{for}\;t\in \left[0,T\right],\\ \; & \int_{\Omega }v_{3}\left(x,t\right)\varphi_{3}\left(x\right)dx-\int_{\Omega }v_{3}^{0}\left(x\right)\varphi_{3}\left(x\right)dx\\ = & -\int_{0}^{t}\int_{\Omega }\varphi_{3}\left(x\right)\left(cv_{3}\left(x,s\right)+v_{1}\left(x,s\right)\right)dxds\\ \; & -\int_{0}^{t}\int_{\Omega }D_{3}\left(x\right)\nabla^{⊤}v_{3}\left(x,s\right)\nabla \varphi_{3}\left(x\right)dxds\;\mathrm{for}\;t\in \left[0,T\right],\\ \; & \int_{\Omega }v_{4}\left(x,t\right)\varphi_{4}\left(x\right)dx-\int_{\Omega }v_{4}^{0}\left(x\right)\varphi_{4}\left(x\right)dx\\ = & -\int_{0}^{t}\int_{\Omega }\varphi_{4}\left(x\right)\left(\alpha v_{4}\left(x,s\right)+\beta v_{1}\left(x,s\right)v_{2}\left(x,s\right)\right)dxds\\ \; & -\int_{0}^{t}\int_{\Omega }D_{4}\left(x\right)\nabla^{⊤}v_{4}\left(x,s\right)\nabla \varphi_{4}\left(x\right)dxds\;\mathrm{for}\;t\in \left[0,T\right]. \end{array}$$

*Let 0<T⩽+∞. The quadruple ${\left(v_{1},v_{2},v_{3},v_{4}\right)}^{⊤}$ is said to be a weak solution to the initial-boundary value problem (3)–(7), or a trajectory of the financial system (3) satisfying (7), in the interval [0,T), provided that the restriction ${\left(v_{1},v_{2},v_{3},v_{4}\right)}^{⊤}{|}_{\Omega \times [0,\tilde{T}]}$, for every $0<\tilde{T}<T$, is a weak solution to the initial-boundary value problem (3)–(7), or a trajectory of the financial system (3) satisfying (7), in the interval $\left[0,\tilde{T}\right]$.*

Definition 3 is essential in our later presentation of this paper. As will be seen later, before we establish fixed-time synchronization results for the drive financial system (3) and the controlled response financial system (5), we shall prove that all trajectories of these two financial systems exist globally in time in the sense of Definition 3.

**Assumption** **1.** *1⩽N⩽2 is an integer; Ω, required to have a $C^{4}$ boundary, designated by ∂Ω, is a bounded connected open subset of the Euclidean space $R^{N}$.*

**Assumption** **2.** *Let k=1,2,3,4. $D_{k}\in C^{3}\left(\bar{\Omega }\right)$, the totality of uniformly continuous functions of which all first-order partial derivatives are uniformly continuous. We write henceforth*

(10) $${\underline{D}}_{k}=\underset{x\in \Omega }{\inf}D_{k}\left(x\right)>0.$$

**Remark** **4.** *Let k=1,2,3,4. Since the domain Ω concerned in this paper is bounded and $D_{k}$ is uniformly continuous on the domain Ω (see Assumption 2), $D_{k}$ is bounded on the domain Ω. We shall write in the rest of the paper*

$${\bar{D}}_{k}=\underset{x\in \Omega }{\sup}D_{k}\left(x\right).$$

Our main contributions in this paper are delineated as follows:

- We introduce diffusion terms to the hyperchaotic financial system (2) to stress that the aftereffect (or memory) in economy and regional disparities of economic development cannot always be neglected, and equip these semi-linear parabolic partial differential equations with the homogeneous boundary condition, thus obtaining the principal research object of this paper, i.e., (3). To the best of our knowledge, the research object of Reference [11] is most closely related to our research object in this paper, and the research aims of References [9,12,13,14] are most closely related to our aims in this paper. However, as remarked above, the systems concerned in References [9,12,13,14] are hyperchaotic financial systems (1) incorporating diffusion terms. The inclusion of diffusion terms in the hyperchaotic financial system (2), and the coefficients of the diffusion terms as functions in Ω, facilitate our application of theoretical results concerning the system (3) obtained in this paper to coming up with suggestions for decision-making in real-world finance or economics.
- We prove rigorously that the initial-boundary value problem (3)–(7) is globally well posed in lower regularity space $L^{2}\left(\Omega ;R^{4}\right)$ in Hadamard’s sense: for every initial datum in $L^{2}\left(\Omega ;R^{4}\right)$, the initial-boundary value problem (3)–(7) admits a unique global solution; in addition, the data-to-solution map is continuous. As alluded in Reference [11], the initial-boundary value problem (3)–(7) admits mild solutions; we find in this paper that mild solutions coincide with weak solutions to the initial-boundary value problem (3)–(7). We provide this assertion a complete rigorous proof via Galerkin’s method and by establishing two a priori estimates, and prove via utilizing the aforementioned a priori estimates that all solutions to the initial-boundary value problem (3)–(7) exist globally in time. Furthermore, we prove, via exploiting semigroup theory, two new assertions (which have not been claimed in Reference [11] or any other published paper): there exists a unique global weak (or equivalently, mild) solution in the Fréchet space $C(R^{+};L^{2}\left(\Omega ;R^{4}\right))$ corresponding to every initial datum in $L^{2}\left(\Omega ;R^{4}\right)$, thus defining a mapping of the Hilbert space $L^{2}\left(\Omega ;R^{4}\right)$ into the Fréchet space $C(R^{+};L^{2}\left(\Omega ;R^{4}\right))$; the aforementioned mapping is continuous.
- We come up with a synchronization control for the response system corresponding to the drive financial system (3), and provide two criteria ensuring that the drive system (3) and its response system with the proposed control implemented achieve fixed-time synchronization. To the authors’ knowledge, among the results in the vast references concerning synchronization problems for (hyper)chaotic financial systems, only the results in References [30,31,32], whose main contributions were introduced briefly above, are highly close to our fixed-time synchronization results in this paper. The results in Reference [30] are concerned with finite-time synchronizability of the time-fractional-order (in the Grünwald–Letnikov sense) counterpart of the financial system (2). The results in Reference [31] are concerned with the fixed-time synchronizability of the financial system (1). The results in Reference [32] are concerned with the fixed-time synchronizability of the time-fractional-order (in Caputo’s sense) counterpart of the financial system (1). In view of these summaries, we conclude that our fixed-time synchronization results in this paper are indeed new.

*Notational Conventions.* We write N for the totality of positive integers. $C^{k}\left(\bar{\Omega }\right)$ with *k* as a positive integer denotes the totality of bounded uniformly continuous functions defined in Ω whose partial derivatives of orders not exceeding *k* are bounded uniformly continuous functions in Ω. We denote by $C\left(\bar{\Omega }\right)=C^{0}\left(\bar{\Omega }\right)$ the totality of bounded uniformly continuous functions defined in Ω. $R^{k}$ with *k* as a positive integer denotes the *k* dimensional Euclidean space. $L^{p}\left(\Omega \right)$ denotes the usual Lebesgue space, 1⩽p⩽+∞. $H^{k}\left(\Omega \right)$ with *k* as a positive integer denotes the totality of square-integrable functions in Ω whose partial derivatives, in the distributional sense, of orders not exceeding *k*, are square-integrable functions in Ω. $D^{+}$ denotes the upper-right Dini derivative with respect to the time variable *t*.

The rest of this paper is organized as follows. In Section 2, we prove via standard Galerkin’s method that for all initial data in $L^{2}\left(\Omega ;R^{4}\right)$, the initial-boundary value problem (3)–(7) admits local weak solutions in the sense of Definition 3; we establish two a priori estimates which play important roles in guaranteeing our successful application of Galerkin’s scheme to obtain the desired local existence; we prove, with the aid of the aforementioned two estimates, that solutions to the initial-boundary value problem (3)–(7) actually exist globally in time; and we leave the proof of the uniqueness and continuous dependence of solutions on initial data to the Appendix (see Appendix A). In Section 3, we design a synchronization control candidate, namely (48), for the response system (5) of the financial system (3); we prove, with the new global well-posedness of the initial-boundary value problem (3)–(7) as the main ingredient, the global existence and uniqueness of the initial-boundary value problem (5)–(8)–(48); we provide a criterion ensuring that the drive system (3) and the response system (5), with the designed control (48) implemented, can achieve fixed-time synchronization; and we discuss the possibility of improving the synchronization control. In Section 4, we perform several numerical simulations to ‘verify’ the effectiveness of the synchronization control (48). In Section 5, we present several concluding remarks.

## 2. Global Well-Posedness of the Initial-Boundary Value Problem (3)–(7)

### 2.1. Preliminaries

As indicated previously, it is relatively easy to prove the global existence of trajectories of models (1) and (2). Actually, it is obvious that the right-hand sides of models (1) and (2) are both locally Lipschitz continuous. By the Cauchy–Lipschitz theory of ordinary differential equations, this implies the local existence of models (1) and (2). On the other hand, for every trajectory ${\left(v_{1},v_{2},v_{3}\right)}^{⊤}$ of model (1), it holds that

(11) $$\begin{array}{cc} \frac{d}{dt}\sum_{k=1}^{3}{\left|v_{k}\left(t\right)\right|}^{2}= & 2\left(v_{2}\left(t\right)-a\right)|v_{1}{\left(t\right)|}^{2}+2v_{1}\left(t\right)v_{3}\left(t\right)-2b{\left|v_{2}\left(t\right)\right|}^{2}\\ \; & -2|v_{1}{\left(t\right)|}^{2}v_{2}\left(t\right)+2v_{2}\left(t\right)-2c{\left|v_{3}\left(t\right)\right|}^{2}-2v_{1}\left(t\right)v_{3}\left(t\right)\\ = & -2a|v_{1}{\left(t\right)|}^{2}-2b|v_{2}{\left(t\right)|}^{2}-2c{\left|v_{3}\left(t\right)\right|}^{2}+2v_{2}\left(t\right),\;t\in R^{+}, \end{array}$$

and for every trajectory ${\left(v_{1},v_{2},v_{3},v_{4}\right)}^{⊤}$ of model (2), it holds similarly that

(12) $$\begin{array}{cc} \; & \frac{d}{dt}\left(\left(2+\beta^{2}\right){\left|v_{1}\left(t\right)\right|}^{2}+2{\left|v_{2}\left(t\right)\right|}^{2}+\sum_{k=3}^{4}{\left|v_{k}\left(t\right)\right|}^{2}+2\beta v_{1}\left(t\right)v_{4}\left(t\right)\right)\\ = & 2\left(2+\beta^{2}\right)\left(|v_{1}{\left(t\right)|}^{2}v_{2}\left(t\right)-a{\left|v_{1}\left(t\right)\right|}^{2}+v_{1}\left(t\right)v_{3}\left(t\right)+v_{1}\left(t\right)v_{4}\left(t\right)\right)\\ \; & +4\left(v_{2}\left(t\right)-b|v_{2}{\left(t\right)|}^{2}-{\left|v_{1}\left(t\right)\right|}^{2}v_{2}\left(t\right)\right)\\ \; & -2\left(c|v_{3}{\left(t\right)|}^{2}+v_{1}\left(t\right)v_{3}\left(t\right)\right)-2\left(\alpha |v_{4}{\left(t\right)|}^{2}+\beta v_{1}\left(t\right)v_{2}\left(t\right)v_{4}\left(t\right)\right)\\ \; & -2\beta v_{1}\left(t\right)\left(\alpha v_{4}\left(t\right)+\beta v_{1}\left(t\right)v_{2}\left(t\right)\right)\\ \; & +2\beta v_{4}\left(t\right)\left(v_{1}\left(t\right)v_{2}\left(t\right)-av_{1}\left(t\right)+v_{3}\left(t\right)+v_{4}\left(t\right)\right)\\ = & -2a\left(2+\beta^{2}\right){\left|v_{1}\left(t\right)\right|}^{2}+2\left(1+\beta^{2}\right)v_{1}\left(t\right)v_{3}\left(t\right)\\ \; & +2\left(2+\beta^{2}-\alpha \beta -a\beta \right)v_{1}\left(t\right)v_{4}\left(t\right)-4b{\left|v_{2}\left(t\right)\right|}^{2}\\ \; & -2c|v_{3}{\left(t\right)|}^{2}+2\beta v_{3}\left(t\right)v_{4}\left(t\right)+2\left(\beta -\alpha \right){\left|v_{4}\left(t\right)\right|}^{2}+4v_{2}\left(t\right),\;t\in R^{+}. \end{array}$$

With the aid of local existence theory and the a priori differential identities, (11) and (12), we can prove the global existence of models (1) and (2) via a standard continuation argument. Let us mention that the a priori differential identity (11) could lead to ultimate boundedness of trajectories of the financial system (1). This topic of boundedness of the trajectories of the system (1) was investigated, via an approach different from ours in this paper, by Rao and Li [14].

Now, we are in a position to prove the global existence of the model (3). The weak solution to the boundary value problem (3) is closely related to the mild solution associated to the strongly continuous semigroup generated by the dynamics of (3).

In this paragraph, we fix k=1,2,3,4 arbitrarily. Let us define an unbounded linear operator in the Hilbert space $L^{2}\left(\Omega \right)$ by

(13) $$\begin{array}{cc} {𝒜}_{k}:L^{2}\left(\Omega \right)⊃D\left(A_{k}\right) & \to L^{2}\left(\Omega \right),\\ D(A_{k})∋\varphi & \mapsto \mathrm{div}(D_{k}\nabla \varphi ), \end{array}$$

in which $D_{k}$ is given as in the model (3), and

(14) $$D\left(A_{k}\right)=\left\{\varphi \in H^{2}\left(\Omega \right);\;\partial_{\nu }\varphi =0\right\}.$$

Owing to Assumption 2, we have, by ([38], Theorem 2.7, p. 211), that $A_{k}$, given by (13) along with (14), is exactly the infinitesimal generator of an analytic semigroup ${\left\{e^{tA_{k}}\right\}}_{t\in [0,+\infty )}$ of contraction operators on the Hilbert space $L^{2}\left(\Omega \right)$. With the help of semigroup theory, the definition of solutions to to the initial-boundary value problem (3)–(7) can be reformulated as follows, appealing to Duhamel’s principle.

**Definition** **4.** *Given ${\left(v_{1}^{0},v_{2}^{0},v_{3}^{0},v_{4}^{0}\right)}^{⊤}\in L^{2}\left(\Omega ;R^{4}\right)$. Let T∈(0,+∞). The quadruple*

$${\left(v_{1},v_{2},v_{3},v_{4}\right)}^{⊤}\in C\left(\left[0,T\right];L^{2}\left(\Omega ;R^{4}\right)\right)\cap L^{2}\left(0,T;H^{1}\left(\Omega ;R^{4}\right)\right)$$

*is said to be a mild solution to to the initial-boundary value problem (3)–(7), in the interval [0,T], provided that the quadruple ${\left(v_{1},v_{2},v_{3},v_{4}\right)}^{⊤}$ satisfies the following: For every t∈[0,T], it holds that*

$$\begin{array}{cc} \; & v_{1}\left(\cdot ,t\right)=e^{tA_{1}}v_{1}^{0}+\int_{0}^{t}e^{\left(t-s\right)A_{1}}\left(\left(v_{2}\left(\cdot ,s\right)-a\right)v_{1}\left(\cdot ,s\right)+v_{3}\left(\cdot ,s\right)+v_{4}\left(\cdot ,s\right)\right)ds,\\ \; & v_{2}\left(\cdot ,t\right)=e^{tA_{2}}v_{2}^{0}+\int_{0}^{t}e^{\left(t-s\right)A_{2}}\left(1-bv_{2}\left(\cdot ,s\right)-{\left(v_{1}\left(\cdot ,s\right)\right)}^{2}\right)ds,\\ \; & v_{3}\left(\cdot ,t\right)=e^{tA_{3}}v_{3}^{0}-\int_{0}^{t}e^{\left(t-s\right)A_{3}}\left(cv_{3}\left(\cdot ,s\right)+v_{1}\left(\cdot ,s\right)\right)ds,\\ \; & v_{4}\left(\cdot ,t\right)=e^{tA_{4}}v_{4}^{0}-\int_{0}^{t}e^{\left(t-s\right)A_{4}}\left(\alpha v_{4}\left(\cdot ,s\right)+\beta v_{1}\left(\cdot ,s\right)v_{2}\left(\cdot ,s\right)\right)ds. \end{array}$$

*Let 0<T⩽+∞. The quadruple ${\left(v_{1},v_{2},v_{3},v_{4}\right)}^{⊤}$ is said to be a mild solution to the initial-boundary value problem (3)–(7), in the interval [0,T), provided that the restriction*

$${\left(v_{1},v_{2},v_{3},v_{4}\right)}^{⊤}{|}_{\Omega \times [0,\tilde{T}]},$$

*for every $0<\tilde{T}<T$, is a mild solution to the problem (3)–(7), in the interval $\left[0,\tilde{T}\right]$.*

The integration in Definition 4 makes sense indeed, due to the smoothing effect in space variables of solutions to the the problem (3). The smoothing effect is attributed to the analyticity of the semigroup ${\left\{e^{tA_{k}}\right\}}_{t\in [0,+\infty )}$. More precisely, we have the following lemma:

**Lemma** **1** (See [39]). *Let Ω be a bounded open subset of $R^{N}$ (without assuming N⩽2) with a $C^{1}$ boundary. There exist $M_{1}>0$ and $M_{2}>0$ such that for every k=1,2,3,4, we have*

(15) $$\begin{array}{cccccc} \; & ∥e^{tA_{k}}{\varphi ∥}_{L^{2}\left(\Omega \right)}⩽M_{1}t^{-\frac{N}{4}}{∥\varphi ∥}_{L^{1}\left(\Omega \right)},\; & \; & \forall \varphi \in L^{1}\left(\Omega \right),\; & \; & \forall t\in (0,+\infty ),\\ \; & ∥e^{tA_{k}}{\varphi ∥}_{L^{2}\left(\Omega \right)}⩽M_{2}t^{-\frac{N}{8}}{∥\varphi ∥}_{L^{\frac{4}{3}}\left(\Omega \right)},\; & \; & \forall \varphi \in L^{\frac{4}{3}}\left(\Omega \right),\; & \; & \forall t\in (0,+\infty ), \end{array}$$

*where $A_{k}$ is given by (13).*

To treat the nonlinearity in the model (3) in an appropriate way, it is helpful to recall the following embedding result concerning Sobolev spaces.

**Lemma** **2** (See [40], Theorem 2, p. 279). *Let Ω be a bounded open subset of $R^{N}$ with N⩽4. The Lebesgue space $L^{4}\left(\Omega \right)$ is continuously embedded into the Sobolev space $H^{1}\left(\Omega \right)$; more precisely, there exists an $M_{3}>0$ depending merely on Ω, such that*

(16) $$\begin{array}{cccc} \; & {∥\varphi ∥}_{L^{4}\left(\Omega \right)}⩽M_{3}{∥\varphi ∥}_{H^{1}\left(\Omega \right)},\; & \; & \forall \varphi \in H^{1}\left(\Omega \right). \end{array}$$

In this paragraph, we apply Lemmas 1 and 2 to explain in detail the reason why Definition 4 makes sense. By applying Lemma 2 and the Cauchy–Schwarz inequality, we have after some tedious calculations the following series of inequalities

$$\begin{array}{cc} \; & ∥\int_{0}^{t}e^{\left(t-s\right)A_{1}}v_{1}\left(\cdot ,s\right)v_{2}{\left(\cdot ,s\right)ds∥}_{L^{2}\left(\Omega \right)}\\ ⩽\; & \int_{0}^{t}{∥e^{\left(t-s\right)A_{1}}v_{1}\left(\cdot ,s\right)v_{2}\left(\cdot ,s\right)∥}_{L^{2}\left(\Omega \right)}ds\\ ⩽\; & \int_{0}^{t}{∥v_{1}\left(\cdot ,s\right)v_{2}\left(\cdot ,s\right)∥}_{L^{2}\left(\Omega \right)}ds\\ ⩽\; & \int_{0}^{t}∥v_{1}{\left(\cdot ,s\right)∥}_{L^{4}\left(\Omega \right)}{∥v_{2}\left(\cdot ,s\right)∥}_{L^{4}\left(\Omega \right)}ds\\ ⩽\; & {\left(M_{3}\right)}^{2}\int_{0}^{t}∥v_{1}\left(\cdot ,s\right){∥}_{H^{1}\left(\Omega \right)}{∥v_{2}\left(\cdot ,s\right)∥}_{H^{1}\left(\Omega \right)}ds,\;t\in \left[0,T\right], \end{array}$$

which, together with the Cauchy–Schwarz inequality, implies directly

(17) $$\begin{array}{cc} \; & ∥\int_{0}^{t}e^{\left(t-s\right)A_{1}}v_{1}\left(\cdot ,s\right)v_{2}{\left(\cdot ,s\right)ds∥}_{C(\left[0,T\right];L^{2}\left(\Omega \right))}\\ ⩽ & {\left(M_{3}\right)}^{2}∥v_{1}{∥}_{L^{2}\left(0,T;H^{1}\left(\Omega \right)\right)}{∥v_{2}∥}_{L^{2}\left(0,T;H^{1}\left(\Omega \right)\right)}, \end{array}$$

where $M_{3}$, in this paragraph, is a positive constant given exactly as in (16) in Lemma 2. Additionally, by applying Hölder’s inequality and Lemmas 1 and 2, we have, after some tedious but routine calculations, the following: there exists a positive constant $C_{1T}$ depending merely on *T* and Ω, such that

$$\begin{array}{cc} \; & ∥\nabla \int_{0}^{t}e^{\left(t-s\right)A_{1}}v_{1}\left(\cdot ,s\right)v_{2}{\left(\cdot ,s\right)ds∥}_{L^{2}\left(0,T;L^{2}\left(\Omega \right)\right)}\\ ⩽\; & ∥\int_{0}^{t}e^{\left(t-s\right)A_{1}}\left(v_{1}\left(\cdot ,s\right)\nabla v_{2}\left(\cdot ,s\right)+v_{2}\left(\cdot ,s\right)\nabla v_{1}\left(\cdot ,s\right)\right){ds∥}_{L^{2}\left(0,T;L^{2}\left(\Omega \right)\right)}\\ \; & +C_{1T}∥v_{1}{∥}_{L^{2}\left(0,T;H^{1}\left(\Omega \right)\right)}{∥v_{2}∥}_{L^{2}\left(0,T;H^{1}\left(\Omega \right)\right)}\\ ⩽\; & \frac{2M_{2}}{\sqrt{4-N}}T^{\frac{4-N}{8}}∥v_{1}\nabla v_{2}+v_{2}\nabla v_{1}{∥}_{L^{1}\left(0,T;L^{\frac{4}{3}}\left(\Omega \right)\right)}+C_{1T}∥v_{1}{∥}_{L^{2}\left(0,T;H^{1}\left(\Omega \right)\right)}{∥v_{2}∥}_{L^{2}\left(0,T;H^{1}\left(\Omega \right)\right)}\\ ⩽\; & \frac{2M_{2}}{\sqrt{4-N}}T^{\frac{4-N}{8}}(∥v_{1}{∥}_{L^{2}\left(0,T;L^{4}\left(\Omega \right)\right)}{∥\nabla v_{2}∥}_{L^{2}\left(0,T;L^{2}\left(\Omega ;R^{4}\right)\right)}\\ \; & +∥v_{2}{∥}_{L^{2}\left(0,T;L^{4}\left(\Omega \right)\right)}{∥\nabla v_{1}∥}_{L^{2}\left(0,T;L^{2}\left(\Omega ;R^{4}\right)\right)})\\ \; & +C_{1T}∥v_{1}{∥}_{L^{2}\left(0,T;H^{1}\left(\Omega \right)\right)}{∥v_{2}∥}_{L^{2}\left(0,T;H^{1}\left(\Omega \right)\right)}\\ ⩽\; & \left(C_{1T}+\frac{4M_{2}M_{3}}{\sqrt{4-N}}T^{\frac{4-N}{8}}\right)∥v_{1}{∥}_{L^{2}\left(0,T;H^{1}\left(\Omega \right)\right)}{∥v_{2}∥}_{L^{2}\left(0,T;H^{1}\left(\Omega \right)\right)}. \end{array}$$

This, together with (17), implies

(18) $$\begin{array}{cc} \; & ∥\int_{0}^{t}e^{\left(t-s\right)A_{1}}v_{1}\left(\cdot ,s\right)v_{2}{\left(\cdot ,s\right)ds∥}_{L^{2}\left(0,T;H^{1}\left(\Omega \right)\right)}\\ ⩽\; & C_{2T}∥v_{1}{∥}_{L^{2}\left(0,T;H^{1}\left(\Omega \right)\right)}{∥v_{2}∥}_{L^{2}\left(0,T;H^{1}\left(\Omega \right)\right)}, \end{array}$$

where $C_{2T}$ is a positive constant depending merely on *T* and Ω. Analogously, we have

(19) $$∥\int_{0}^{t}e^{\left(t-s\right)A_{2}}{\left(v_{1}\left(\cdot ,s\right)\right)}^{2}{ds∥}_{C(\left[0,T\right];L^{2}\left(\Omega \right))}⩽{\left(M_{3}\right)}^{2}{∥v_{1}∥}_{L^{2}\left(0,T;H^{1}\left(\Omega \right)\right)}^{2};$$

(20) $$∥\int_{0}^{t}e^{\left(t-s\right)A_{2}}{\left(v_{1}\left(\cdot ,s\right)\right)}^{2}{ds∥}_{L^{2}\left(0,T;H^{1}\left(\Omega \right)\right)}⩽C_{3T}{∥v_{1}∥}_{L^{2}\left(0,T;H^{1}\left(\Omega \right)\right)}^{2},$$

where $C_{3T}$ is a positive constant depending merely on *T* and Ω;

(21) $$\begin{array}{cc} \; & ∥\int_{0}^{t}e^{\left(t-s\right)A_{4}}v_{1}\left(\cdot ,s\right)v_{2}{\left(\cdot ,s\right)ds∥}_{C(\left[0,T\right];L^{2}\left(\Omega \right))}\\ ⩽\; & {\left(M_{3}\right)}^{2}∥v_{1}{∥}_{L^{2}\left(0,T;H^{1}\left(\Omega \right)\right)}{∥v_{2}∥}_{L^{2}\left(0,T;H^{1}\left(\Omega \right)\right)}; \end{array}$$

and there exists a positive constant $C_{4T}$ depending merely on *T* and Ω, such that

$$\begin{array}{cc} \; & ∥\int_{0}^{t}e^{\left(t-s\right)A_{4}}v_{1}\left(\cdot ,s\right)v_{2}{\left(\cdot ,s\right)ds∥}_{L^{2}\left(0,T;H^{1}\left(\Omega \right)\right)}\\ ⩽\; & C_{4T}∥v_{1}{∥}_{L^{2}\left(0,T;H^{1}\left(\Omega \right)\right)}{∥v_{2}∥}_{L^{2}\left(0,T;H^{1}\left(\Omega \right)\right)}. \end{array}$$

This, together with (17)–(21), guarantees that Definition 4 makes sense. To end this paragraph, let us point that if $D_{1}\left(x\right)$, $D_{2}\left(x\right)$, $D_{3}\left(x\right)$, and $D_{4}\left(x\right)$ are all constants, then

$$C_{2T}=C_{3T}=C_{3T}=M_{3}\sqrt{\frac{16{\left(M_{2}\right)}^{2}}{4-N}T^{\frac{4-N}{4}}+T{\left(M_{3}\right)}^{2}}.$$

It is worth mentioning that, for the Neumann boundary value problem (3), classical solutions (see Definition 2) are weak solutions (see Definition 3) and also are mild solutions (see Definition 4); weak solutions are mild solutions, and vice versa. By the Rellich–Kondrachov theorem, we conclude that the unbounded operator $A_{k}$ has compact resolvents. Therefore, the spectrum $\sigma (A_{k})$ consists merely of eigenvalues of $A_{k}$. More detailed information in this direction can be reformulated as follows. Aided by Assumption 2, by recalling the theory of boundary value problems for elliptic partial differential equations, we know that, for k=1,2,3,4, the homogeneous Neumann problem

(22) $$\left\{\begin{array}{cc} -\mathrm{div}\left(D_{k}\nabla \psi_{k}\right)=\lambda \psi_{k} & \mathrm{in}\;\Omega ,\\ \partial_{\nu }v_{k}=0 & \mathrm{on}\;\partial \Omega \end{array}\right.$$

admits a sequence $\left\{\frac{1}{\sqrt{\mathrm{meas}\Omega }};\;\psi_{kn},\;n\in N\right\}$ of solutions which form an orthonormal basis for the Hilbert space $L^{2}\left(\Omega \right)$; we assume in the rest of the paper that the sequence $\left\{\frac{1}{\sqrt{\mathrm{meas}\Omega }};\;\psi_{kn},\;n\in N\right\}$ is arranged so that the sequence $\left\{0;\;\lambda_{kn},\;n\in N\right\}$ of corresponding eigenvalues satisfies

(23) $$0<\lambda_{kn}⩽\lambda_{km}$$

whenever n<m, m,n∈N. We write hereafter $\psi_{k0}=\frac{1}{\sqrt{\mathrm{meas}\Omega }}$ and $\lambda_{k0}=0$.

**Remark** **5.** *Since the boundary ∂Ω belongs to the class $C^{4}$ and $D_{k}\in C^{3}\left(\bar{\Omega }\right)$, by regularity theory of elliptic partial differential equations (see [40], pp. 326–346), solutions to the eigenvalue problem (31) belong to $H^{4}\left(\Omega \right)$. Thanks to 1⩽N⩽2 (see Assumption 1), $H^{4}\left(\Omega \right)\subset C^{2}\left(\bar{\Omega }\right)$; see ([38], Theorem 1.2, p. 208).*

### 2.2. Two Useful a Priori Inequalities

Let 0<T⩽+∞. To every quadruple

$${\left(v_{1},v_{2},v_{3},v_{4}\right)}^{⊤}\in C\left(\left[0,T\right);L^{2}\left(\Omega ;R^{4}\right)\right)\cap L_{\mathrm{loc}}^{2}\left(\left[0,T\right);H^{1}\left(\Omega ;R^{4}\right)\right),$$

we associate the functional

(24) $$\begin{array}{cc} \Psi_{\varepsilon }^{v_{1},v_{2},v_{3},v_{4}}\left(t\right)= & \left(1+\beta^{2}+\varepsilon \right)\int_{\Omega }|v_{1}\left(x,t\right){|}^{2}dx+\left(1+\varepsilon \right)\int_{\Omega }{\left|v_{2}\left(x,t\right)\right|}^{2}dx\\ \; & +\sum_{k=3}^{4}\int_{\Omega }|v_{k}{\left(x,t\right)|}^{2}dx+2\left(1+\beta^{2}+\varepsilon \right)\int_{0}^{t}\int_{\Omega }D_{1}\left(x\right){\left|\nabla v_{1}\left(x,s\right)\right|}^{2}dxds\\ \; & +2\left(1+\varepsilon \right)\int_{0}^{t}\int_{\Omega }D_{2}\left(x\right){\left|\nabla v_{2}\left(x,s\right)\right|}^{2}dxds\\ \; & +2\sum_{k=3}^{4}\int_{0}^{t}\int_{\Omega }D_{k}\left(x\right){\left|\nabla v_{k}\left(x,s\right)\right|}^{2}dxds+2\beta \int_{\Omega }v_{1}\left(x,t\right)v_{4}\left(x,t\right)dx\\ \; & +2a\left(1+\beta^{2}+\varepsilon \right)\int_{0}^{t}\int_{\Omega }|v_{1}\left(x,s\right){|}^{2}dxds+2b\left(1+\varepsilon \right)\int_{0}^{t}\int_{\Omega }{\left|v_{2}\left(x,s\right)\right|}^{2}dxds\\ \; & +2\int_{0}^{t}\int_{\Omega }\left(c|v_{3}{\left(x,s\right)|}^{2}+\alpha {\left|v_{4}\left(x,s\right)\right|}^{2}\right)dxds\\ \; & +2\beta \int_{0}^{t}\int_{\Omega }\left(D_{1}\left(x\right)+D_{4}\left(x\right)\right)\nabla^{⊤}v_{1}\left(x,s\right)\nabla v_{4}\left(x,s\right)dxds,\;t\in \left[0,T\right), \end{array}$$

and associate the functional

(25) $$\begin{array}{cc} \Phi_{\varepsilon }^{v_{1},v_{2},v_{3},v_{4}}\left(t\right)= & \sum_{k=1}^{4}∥v_{k}{\left(\cdot ,t\right)∥}_{L^{2}\left(\Omega \right)}^{2}+2\sum_{k=1}^{4}\int_{0}^{t}{∥\nabla v_{k}\left(\cdot ,s\right)∥}_{L^{2}\left(\Omega ;R^{N}\right)}^{2}ds,\;t\in \left[0,T\right). \end{array}$$

By applying the Cauchy–Schwarz inequality, utilizing Lemma 2, and performing some tedious calculations, we can prove that there exists an $M_{4}>0$, depending merely on *a*, *b*, *c*, α, β, ε, Ω, $D_{k}$ (k=1,2,3,4), but independent of *t* and of $v_{k}$ (k=1,2,3,4), such that

(26) $$\begin{array}{cccc} \; & \Psi_{\varepsilon }^{v_{1},v_{2},v_{3},v_{4}}\left(t\right)⩽M_{4}\Phi_{\varepsilon }^{v_{1},v_{2},v_{3},v_{4}}\left(t\right), & \; & t\in [0,T). \end{array}$$

Analogously, we can prove that when ε is sufficiently large, for instance,

$$\varepsilon >\max\left(\frac{\beta^{2}{\left({\bar{D}}_{1}+{\bar{D}}_{2}\right)}^{2}}{4{\underline{D}}_{1}{\underline{D}}_{2}}-1-\beta^{2},0\right),$$

there exists an $M_{5}>0$, depending merely on *a*, *b*, *c*, α, β, ε, Ω, $D_{k}$ (k=1,2,3,4), but independent of *t* and of $v_{k}$ (k=1,2,3,4), such that

(27) $$\begin{array}{cccc} \; & \Psi_{\varepsilon }^{v_{1},v_{2},v_{3},v_{4}}\left(t\right)⩾M_{5}\Phi_{\varepsilon }^{v_{1},v_{2},v_{3},v_{4}}\left(t\right), & \; & t\in [0,T). \end{array}$$

### 2.3. The Global Well-Posedness

**Theorem** **1.** *Suppose that Assumptions 1 and 2 hold true. For every quadruple ${\left(v_{1}^{0},v_{2}^{0},v_{3}^{0},v_{4}^{0}\right)}^{⊤}$ of initial data in the Hilbert space $L^{2}\left(\Omega ;R^{4}\right)$, there exists a*

(28) $$T=T(∥v_{1}^{0}{∥}_{L^{2}\left(\Omega \right)},∥v_{2}^{0}{∥}_{L^{2}\left(\Omega \right)},∥v_{3}^{0}{∥}_{L^{2}\left(\Omega \right)},∥v_{4}^{0}{∥}_{L^{2}\left(\Omega \right)},a,b,c,\alpha ,\beta ,\Omega )>0,$$

*such that the initial-boundary value problem (3)–(7) admits a unique weak solution*

(29) $${\left(v_{1},v_{2},v_{3},v_{4}\right)}^{⊤}\in C\left(\left[0,T\right];L^{2}\left(\Omega ;R^{4}\right)\right)\cap L^{2}\left(0,T;H^{1}\left(\Omega ;R^{4}\right)\right).$$

*in the sense of Definition 3. Furthermore, for every r∈(0,+∞), the data-to-solution map*

$$\begin{array}{cc} \; & \{{\left(\zeta_{1},\zeta_{2},\zeta_{3},\zeta_{4}\right)}^{⊤}\in L^{2}\left(\Omega ;R^{4}\right);\;\max_{1⩽k⩽4}∥\zeta_{k}{∥}_{L^{2}\left(\Omega \right)}⩽r\}∋{\left(v_{1}^{0},v_{2}^{0},v_{3}^{0},v_{4}^{0}\right)}^{⊤}\\ \; & \mapsto {\left(v_{1},v_{2},v_{3},v_{4}\right)}^{⊤}\in C\left(\left[0,T\right];L^{2}\left(\Omega ;R^{4}\right)\right) \end{array}$$

*is Lipschitz continuous, where T depends merely on r, a, b, c, α, β, Ω.*

**Proof.**  The uniqueness and continuous dependence parts are not as close as the existence part to the fixed-time synchronization problem concerned later in this paper. We relegate the proof of the uniqueness and continuous dependence parts into the Appendix, and write down in detail the proof of the existence part here. We shall prove the existence part of Theorem 1 by a standard Galerkin procedure. First, we assume that for every positive integer *n*, the homogeneous Neumann problem (3) supplemented by the initial condition

(30) $$\left\{\begin{array}{ccc} v_{1n}\left(x,0\right)=\sum_{\ell =0}^{n}\int_{\Omega }v_{1}^{0}\left(x\right)\psi_{1\ell }\left(x\right)dx\psi_{1\ell }\left(x\right)\; & \; & \mathrm{for}\;x\in \Omega ,\\ v_{2n}\left(x,0\right)=\sum_{\ell =0}^{n}\int_{\Omega }v_{2}^{0}\left(x\right)\psi_{2\ell }\left(x\right)dx\psi_{2\ell }\left(x\right)\; & \; & \mathrm{for}\;x\in \Omega ,\\ v_{3n}\left(x,0\right)=\sum_{\ell =0}^{n}\int_{\Omega }v_{3}^{0}\left(x\right)\psi_{3\ell }\left(x\right)dx\psi_{3\ell }\left(x\right)\; & \; & \mathrm{for}\;x\in \Omega ,\\ v_{4n}\left(x,0\right)=\sum_{\ell =0}^{n}\int_{\Omega }v_{4}^{0}\left(x\right)\psi_{4\ell }\left(x\right)dx\psi_{k\ell }\left(x\right)\; & \; & \mathrm{for}\;x\in \Omega , \end{array}\right.$$

admits a solution ${\left(v_{1n},v_{1n},v_{1n},v_{1n}\right)}^{⊤}$ of the form

(31) $$\left\{\begin{array}{ccc} v_{1n}\left(x,t\right)=\sum_{\ell =0}^{n}{\hat{v}}_{1n\ell }\left(t\right)\psi_{1\ell }\left(x\right)\; & \; & \mathrm{for}\;(x,t)\in \Omega \times [0,+\infty ),\\ v_{2n}\left(x,t\right)=\sum_{\ell =0}^{n}{\hat{v}}_{2n\ell }\left(t\right)\psi_{2\ell }\left(x\right)\; & \; & \mathrm{for}\;(x,t)\in \Omega \times [0,+\infty ),\\ v_{3n}\left(x,t\right)=\sum_{\ell =0}^{n}{\hat{v}}_{3n\ell }\left(t\right)\psi_{3\ell }\left(x\right)\; & \; & \mathrm{for}\;(x,t)\in \Omega \times [0,+\infty ),\\ v_{4n}\left(x,t\right)=\sum_{\ell =0}^{n}{\hat{v}}_{4n\ell }\left(t\right)\psi_{4\ell }\left(x\right)\; & \; & \mathrm{for}\;(x,t)\in \Omega \times [0,+\infty ). \end{array}\right.$$

With the aid of (31), we can find readily that initial condition (30) is equivalent to

(32) $$\left\{\begin{array}{ccc} {\hat{v}}_{1n\ell }\left(0\right)=\int_{\Omega }v_{1}^{0}\left(x\right)\psi_{1\ell }\left(x\right)dx\; & \; & \mathrm{for}\;\ell =0,1,\ldots ,n,\\ {\hat{v}}_{2n\ell }\left(0\right)=\int_{\Omega }v_{2}^{0}\left(x\right)\psi_{2\ell }\left(x\right)dx\; & \; & \mathrm{for}\;\ell =0,1,\ldots ,n,\\ {\hat{v}}_{3n\ell }\left(0\right)=\int_{\Omega }v_{3}^{0}\left(x\right)\psi_{3\ell }\left(x\right)dx\; & \; & \mathrm{for}\;\ell =0,1,\ldots ,n,\\ {\hat{v}}_{4n\ell }\left(0\right)=\int_{\Omega }v_{4}^{0}\left(x\right)\psi_{4\ell }\left(x\right)dx\; & \; & \mathrm{for}\;\ell =0,1,\ldots ,n. \end{array}\right.$$

Recalling that ${\left\{\psi_{kn}\right\}}_{n\in N_{0}}$ is an orthonormal basis for $L^{2}\left(\Omega \right)$, k=1,2,3,4, we substitute (31) into (3) and conduct some further routine but tedious calculations, to find that the necessary and sufficient condition for the quadruple ${\left(v_{1n},v_{1n},v_{1n},v_{1n}\right)}^{⊤}$ of functions given by (31) to be a solution to the homogeneous Neumann problem (3) is that

$${\left({\hat{v}}_{1n0}\left(t\right),\ldots ,{\hat{v}}_{1nn}\left(t\right);{\hat{v}}_{2n0}\left(t\right),\ldots ,{\hat{v}}_{2nn}\left(t\right);{\hat{v}}_{3n0}\left(t\right),\ldots ,{\hat{v}}_{3nn}\left(t\right);{\hat{v}}_{4n0}\left(t\right),\ldots ,{\hat{v}}_{4nn}\left(t\right)\right)}^{⊤}.$$

This is the solution to the following system of ordinary differential equations

(33) $$\left.\begin{array}{cc} \frac{d}{dt}{\hat{v}}_{1n\ell }\left(t\right)= & -\left(\lambda_{1\ell }+a\right){\hat{v}}_{1n\ell }\left(t\right)\\ \; & +\sum_{i=0}^{n}\sum_{j=0}^{n}{\hat{v}}_{1ni}\left(t\right){\hat{v}}_{2nj}\left(t\right)\int_{\Omega }\psi_{1i}\left(x\right)\psi_{1\ell }\left(x\right)\psi_{2j}\left(x\right)dx\\ \; & +\sum_{i=0}^{n}{\hat{v}}_{3ni}\left(t\right)\int_{\Omega }\psi_{1\ell }\left(x\right)\psi_{3i}\left(x\right)dx\\ \; & +\sum_{i=0}^{n}{\hat{v}}_{4ni}\left(t\right)\int_{\Omega }\psi_{1\ell }\left(x\right)\psi_{4i}\left(x\right)dx,\\ \frac{d}{dt}{\hat{v}}_{2n\ell }\left(t\right)= & -\left(\lambda_{2\ell }+b\right){\hat{v}}_{2n\ell }\left(t\right)+\int_{\Omega }\psi_{2\ell }\left(x\right)dx\\ \; & -\sum_{i=0}^{n}\sum_{j=0}^{n}{\hat{v}}_{1ni}\left(t\right){\hat{v}}_{1nj}\left(t\right)\int_{\Omega }\psi_{1i}\left(x\right)\psi_{1j}\left(x\right)\psi_{2\ell }\left(x\right)dx,\\ \frac{d}{dt}{\hat{v}}_{3n\ell }\left(t\right)= & -\left(\lambda_{3\ell }+c\right){\hat{v}}_{3n\ell }\left(t\right)\\ \; & -\sum_{i=0}^{n}{\hat{v}}_{1ni}\left(t\right)\int_{\Omega }\psi_{1i}\left(x\right)\psi_{3\ell }\left(x\right)dx,\\ \frac{d}{dt}{\hat{v}}_{4n\ell }\left(t\right)= & -\left(\lambda_{4\ell }+\alpha \right){\hat{v}}_{4n\ell }\left(t\right)\\ \; & -\beta \sum_{i=0}^{n}\sum_{j=0}^{n}{\hat{v}}_{1ni}\left(t\right){\hat{v}}_{2nj}\left(t\right)\int_{\Omega }\psi_{1i}\left(x\right)\psi_{2j}\left(x\right)\psi_{4\ell }\left(x\right)dx, \end{array}\right\}\;\ell =0,\ldots ,n.$$

By observing that ordinary differential equations in the system (33) have local Lipschitz continuous nonlinearity, we can apply the Cauchy–Lipschitz existence theorem to obtain the following: There exists a *T* as in (28) such that (33) admits a unique solution in the interval [0,T]. In light of Remark 5, we find that

(34) $${\left(v_{1n},v_{2n},v_{3n},v_{4n}\right)}^{⊤}\in C^{1}\left(\left[0,T\right];H^{4}\left(\Omega ;R^{4}\right)\right).$$

To prove Theorem 1 for general initial data, we need some a priori estimates for solutions to the homogeneous Neumann problem (3). To this end, we associate, in the rest of this proof, to every solution quadruple given in (31) (see also (34)), the modified energy functional

(35) $$\begin{array}{cccc} \; & E\left(t\right)=\Psi_{\varepsilon }^{v_{1n},v_{2n},v_{3n},v_{4n}}\left(t\right), & \; & t\in [0,T]; \end{array}$$

see (24) for the definition of the functional $\Psi_{\varepsilon }^{v_{1},v_{2},v_{3},v_{4}}\left(t\right)$. To proceed further, let us recall that the quadruple (31) (see also (34)) satisfies

(36) $$\left\{\begin{array}{ccc} \partial_{t}v_{1n}=\mathrm{div}\left(D_{1}\nabla v_{1n}\right)+\left(v_{2n}-a\right)v_{1n}+v_{3n}+v_{4n} & \; & \mathrm{in}\;\Omega \times (0,T),\\ \partial_{t}v_{2n}=\mathrm{div}\left(D_{2}\nabla v_{2n}\right)-bv_{2n}-{\left(v_{1n}\right)}^{2}+1 & \; & \mathrm{in}\;\Omega \times (0,T),\\ \partial_{t}v_{3n}=\mathrm{div}\left(D_{3}\nabla v_{3n}\right)-cv_{3n}-v_{1n} & \; & \mathrm{in}\;\Omega \times (0,T),\\ \partial_{t}v_{4n}=\mathrm{div}\left(D_{4}\nabla v_{4n}\right)-\alpha v_{4n}-\beta v_{1n}v_{2n} & \; & \mathrm{in}\;\Omega \times (0,T),\\ \partial_{\nu }v_{1n}=\partial_{\nu }v_{2n}=\partial_{\nu }v_{3n}=\partial_{\nu }v_{4n}=0 & \; & \mathrm{on}\;\partial \Omega \times [0,T]. \end{array}\right.$$

Differentiating both sides of (35), taking integration by parts, and conducting some other routine calcuations, we deduce with the aid of (36) that

(37) $$\begin{array}{cc} E^{\prime }\left(t\right)= & 2\left(1+\beta^{2}+\varepsilon \right)\int_{\Omega }v_{1n}\left(x,t\right)\partial_{t}v_{1n}\left(x,t\right)dx+2\left(1+\varepsilon \right)\int_{\Omega }v_{2n}\left(x,t\right)\partial_{t}v_{2n}\left(x,t\right)dx\\ \; & +2\sum_{k=3}^{4}\int_{\Omega }v_{kn}\left(x,t\right)\partial_{t}v_{kn}\left(x,t\right)dx+2\left(1+\beta^{2}+\varepsilon \right)\int_{\Omega }D_{1}\left(x\right){\left|\nabla v_{1n}\left(x,t\right)\right|}^{2}dx\\ \; & +2\left(1+\varepsilon \right)\int_{\Omega }D_{2}\left(x\right)|\nabla v_{2n}{\left(x,t\right)|}^{2}dx+2\sum_{k=3}^{4}\int_{\Omega }D_{k}\left(x\right){\left|\nabla v_{kn}\left(x,t\right)\right|}^{2}dx\\ \; & +2\beta \int_{\Omega }v_{1n}\left(x,t\right)\partial_{t}v_{4n}\left(x,t\right)dx+2\beta \int_{\Omega }v_{4n}\left(x,t\right)\partial_{t}v_{1n}\left(x,t\right)dx\\ \; & +2a\left(1+\beta^{2}+\varepsilon \right)\int_{\Omega }|v_{1n}\left(x,t\right){|}^{2}dx+2b\left(1+\varepsilon \right)\int_{\Omega }{\left|v_{2n}\left(x,t\right)\right|}^{2}dx\\ \; & +2\int_{\Omega }\left(c|v_{3n}{\left(x,t\right)|}^{2}+\alpha {\left|v_{4n}\left(x,t\right)\right|}^{2}\right)dx\\ \; & +2\beta \int_{\Omega }\left(D_{1}\left(x\right)+D_{4}\left(x\right)\right)\nabla^{⊤}v_{1n}\left(x,t\right)\nabla v_{4n}\left(x,t\right)dx\\ = & 2\left(1+\beta^{2}+\varepsilon \right)\int_{\Omega }|v_{1n}\left(x,t\right){|}^{2}v_{2n}\left(x,t\right)dx-2\left(1+\beta^{2}+\varepsilon \right)\int_{\Omega }D_{1}\left(x\right){\left|\nabla v_{1n}\left(x,t\right)\right|}^{2}dx\\ \; & +2\left(1+\beta^{2}+\varepsilon \right)\int_{\Omega }v_{1n}\left(x,t\right)\left(v_{3n}\left(x,t\right)+v_{4n}\left(x,t\right)\right)dx\\ \; & +2\left(1+\varepsilon \right)\int_{\Omega }v_{2n}\left(x,t\right)dx-2\left(1+\varepsilon \right)\int_{\Omega }D_{2}\left(x\right){\left|\nabla v_{2n}\left(x,t\right)\right|}^{2}dx\\ \; & -2\left(1+\varepsilon \right)\int_{\Omega }{\left|v_{1n}\left(x,t\right)\right|}^{2}v_{2n}\left(x,t\right)dx-2\int_{\Omega }v_{1n}\left(x,t\right)v_{3n}\left(x,t\right)dx\\ \; & -2\int_{\Omega }D_{3}\left(x\right){\left|\nabla v_{3n}\left(x,t\right)\right|}^{2}dx-2\beta \int_{\Omega }v_{1n}\left(x,t\right)v_{2n}\left(x,t\right)v_{4n}\left(x,t\right)dx\\ \; & -2\int_{\Omega }D_{4}\left(x\right)|\nabla v_{4n}{\left(x,t\right)|}^{2}dx+2\left(1+\beta^{2}+\varepsilon \right)\int_{\Omega }D_{1}\left(x\right){\left|\nabla v_{1n}\left(x,t\right)\right|}^{2}dx\\ \; & +2\left(1+\varepsilon \right)\int_{\Omega }D_{2}\left(x\right)|\nabla v_{2n}{\left(x,t\right)|}^{2}dx+2\sum_{k=3}^{4}\int_{\Omega }D_{k}\left(x\right){\left|\nabla v_{kn}\left(x,t\right)\right|}^{2}dx\\ \; & -2\beta \int_{\Omega }D_{4}\left(x\right)\nabla^{⊤}v_{1n}\left(x,t\right)\nabla v_{4n}\left(x,t\right)dx-2\alpha \beta \int_{\Omega }v_{1n}\left(x,t\right)v_{4n}\left(x,t\right)dx\\ \; & -2\beta^{2}\int_{\Omega }{\left|v_{1n}\left(x,t\right)\right|}^{2}v_{2n}\left(x,t\right)dx-2\beta \int_{\Omega }D_{1}\left(x\right)\nabla^{⊤}v_{1n}\left(x,t\right)\nabla v_{4n}\left(x,t\right)dx\\ \; & +2\beta \int_{\Omega }v_{1n}\left(x,t\right)v_{2n}\left(x,t\right)v_{4n}\left(x,t\right)dx+2\beta \int_{\Omega }{\left|v_{4n}\left(x,t\right)\right|}^{2}dx\\ \; & +2\beta \int_{\Omega }v_{4n}\left(x,t\right)\left(v_{3n}\left(x,t\right)-av_{1n}\left(x,t\right)\right)dx\\ \; & +2\beta \int_{\Omega }\left(D_{1}\left(x\right)+D_{4}\left(x\right)\right)\nabla^{⊤}v_{1n}\left(x,t\right)\nabla v_{4n}\left(x,t\right)dx\\ = & \left(1+2\beta^{2}+2\varepsilon \right)\int_{\Omega }v_{1n}\left(x,t\right)v_{3n}\left(x,t\right)dx+2\left(1+\varepsilon \right)\int_{\Omega }v_{2n}\left(x,t\right)dx\\ \; & +2\left(1+\beta^{2}+\varepsilon -\alpha \beta -a\beta \right)\int_{\Omega }v_{1n}\left(x,t\right)v_{4n}\left(x,t\right)dx\\ \; & +2\beta \int_{\Omega }{\left|v_{4n}\left(x,t\right)\right|}^{2}dx+2\beta \int_{\Omega }v_{3n}\left(x,t\right)v_{4n}\left(x,t\right)dx,\;t\in \left[0,T\right]. \end{array}$$

By utilizing the Cauchy–Schwarz inequality, we have immediately

(38) $$\begin{array}{cccc} \; & 2\int_{\Omega }v_{2n}\left(x,t\right)dx⩽\mathrm{meas}\Omega +\int_{\Omega }{\left|v_{2n}\left(x,t\right)\right|}^{2}dx, & \; & t\in [0,T], \end{array}$$

(39) $$\begin{array}{cccc} \; & 2\int_{\Omega }v_{1n}\left(x,t\right)v_{3n}\left(x,t\right)dx⩽\int_{\Omega }|v_{1n}{\left(x,t\right)|}^{2}dx+\int_{\Omega }{\left|v_{3n}\left(x,t\right)\right|}^{2}dx,\; & \; & t\in [0,T], \end{array}$$

(40) $$\begin{array}{cccc} \; & 2\int_{\Omega }v_{1n}\left(x,t\right)v_{4n}\left(x,t\right)dx⩽\int_{\Omega }|v_{1n}{\left(x,t\right)|}^{2}dx+\int_{\Omega }{\left|v_{4n}\left(x,t\right)\right|}^{2}dx,\; & \; & t\in [0,T], \end{array}$$

$$\begin{array}{cccc} \; & 2\int_{\Omega }v_{3n}\left(x,t\right)v_{4n}\left(x,t\right)dx⩽\int_{\Omega }|v_{3n}{\left(x,t\right)|}^{2}dx+\int_{\Omega }{\left|v_{4n}\left(x,t\right)\right|}^{2}dx,\; & \; & t\in [0,T]. \end{array}$$

This, together with (37)–(40) and (27), implies

(41) $$\begin{array}{cc} E^{\prime }\left(t\right)⩽ & M_{6}\sum_{k=1}^{4}{∥v_{kn}\left(\cdot ,t\right)∥}_{L^{2}\left(\Omega \right)}^{2}+\left(1+\varepsilon \right)\mathrm{meas}\Omega \\ ⩽ & M_{6}\Psi_{\varepsilon }^{v_{1n},v_{2n},v_{3n},v_{4n}}\left(t\right)+\left(1+\varepsilon \right)\mathrm{meas}\Omega \\ ⩽ & \frac{M_{6}}{M_{5}}E\left(t\right)+\left(1+\varepsilon \right)\mathrm{meas}\Omega ,\;t\in \left[0,T\right], \end{array}$$

where $\Phi_{\varepsilon }^{v_{1},v_{2},v_{3},v_{4}}\left(t\right)$ is given as in (25), and the constant $M_{6}$ is given by

(42) $$\begin{array}{cc} M_{6}=\max( & \frac{1+2\beta^{2}+2\varepsilon }{2}+\left|1+\beta^{2}+\varepsilon -\alpha \beta -a\beta \right|,1+\varepsilon ,\\ \; & \frac{1+2\beta^{2}+2\varepsilon }{2}+\beta ,\left|1+\beta^{2}+\varepsilon -\alpha \beta -a\beta \right|+3\beta ). \end{array}$$

By applying Gronwall’s Lemma, we deduce from (41) that

$$\begin{array}{c} E\left(t\right)⩽\left(E\left(0\right)+\frac{\left(1+\varepsilon \right)M_{5}\mathrm{meas}\Omega }{M_{6}}\right)e^{\frac{M_{6}}{M_{5}}t}-\frac{\left(1+\varepsilon \right)M_{5}\mathrm{meas}\Omega }{M_{6}},\;t\in \left[0,T\right]. \end{array}$$

This, together with (25) and (27), implies

(43) $$\begin{array}{cc} \; & \sum_{k=1}^{4}∥v_{kn}{\left(\cdot ,t\right)∥}_{L^{2}\left(\Omega \right)}^{2}+2\sum_{k=1}^{4}\int_{0}^{t}{∥\nabla v_{kn}\left(\cdot ,s\right)∥}_{L^{2}\left(\Omega ;R^{N}\right)}^{2}ds\\ ⩽ & \frac{1}{M_{5}}E\left(t\right)⩽\frac{M_{7}}{M_{5}}e^{\frac{M_{6}}{M_{5}}T}-\frac{(1+\varepsilon )\mathrm{meas}\Omega }{M_{6}},\;t\in \left[0,T\right]. \end{array}$$

(44) $$\begin{array}{cc} M_{7}= & \left(1+\beta^{2}+\varepsilon \right)∥v_{1}^{0}{∥}_{L^{2}\left(\Omega \right)}^{2}+\left(1+\varepsilon \right)∥v_{2}^{0}{∥}_{L^{2}\left(\Omega \right)}^{2}+\sum_{k=3}^{4}{∥v_{k}^{0}∥}_{L^{2}\left(\Omega \right)}^{2}\\ \; & +2\beta ∥v_{1}^{0}{∥}_{L^{2}\left(\Omega \right)}{∥v_{4}^{0}∥}_{L^{2}\left(\Omega \right)}+\frac{\left(1+\varepsilon \right)M_{5}\mathrm{meas}\Omega }{M_{6}} \end{array}$$

From (43), it follows that the sequence ${\left\{{\left(v_{1n},v_{2n},v_{3n},v_{4n}\right)}^{⊤}\right\}}_{n\in N}$ is bounded in the Banach space

$$C\left(\left[0,T\right];L^{2}\left(\Omega ;R^{4}\right)\right)\cap L^{2}\left(0,T;H^{1}\left(\Omega ;R^{4}\right)\right).$$

From this, it follows immediately that the sequence ${\left\{{\left(v_{1n}v_{2n},{\left(v_{1n}\right)}^{2}\right)}^{⊤}\right\}}_{n\in N}$ of pairs is bounded in the Hilbert space $L^{2}\left(0,T;L^{2}\left(\Omega ;R^{2}\right)\right)$. This boundedness, together with (36), implies that the sequence ${\left\{{\left(\partial_{t}v_{1n},\partial_{t}v_{2n},\partial_{t}v_{3n},\partial_{t}v_{4n}\right)}^{⊤}\right\}}_{n\in N}$ is bounded in the Hilbert space $L^{2}\left(0,T;H^{-1}\left(\Omega ;R^{4}\right)\right)$. Therefore, there exists a quadruple

(45) $${\left(v_{1},v_{2},v_{3},v_{4}\right)}^{⊤}\in L^{2}\left(0,T;H^{1}\left(\Omega ;R^{4}\right)\right)$$

with

$${\left(\partial_{t}v_{1},\partial_{t}v_{2},\partial_{t}v_{3},\partial_{t}v_{4}\right)}^{⊤}\in L^{2}\left(0,T;H^{-1}\left(\Omega ;R^{4}\right)\right)$$

such that the sequence ${\left\{{\left(v_{1n},v_{2n},v_{3n},v_{4n}\right)}^{⊤}\right\}}_{n\in N}$ (of solution quadruples) admits a subsequence ${\left\{{\left(v_{1n^{\prime }},v_{2n^{\prime }},v_{3n^{\prime }},v_{4n^{\prime }}\right)}^{⊤}\right\}}_{n^{\prime }\in N}$ satisfying the following: when $n^{\prime }$ tends to *∞*, the following limit assertions hold true

$$\begin{array}{cc} \; & {\left(v_{1n^{\prime }},v_{2n^{\prime }},v_{3n^{\prime }},v_{4n^{\prime }}\right)}^{⊤}\to {\left(v_{1},v_{2},v_{3},v_{4}\right)}^{⊤}\;\mathrm{weakly}\;\mathrm{in}\;L^{2}\left(0,T;H^{1}\left(\Omega ;R^{4}\right)\right),\\ \; & {\left(v_{1n^{\prime }}v_{2n^{\prime }},{\left(v_{1n^{\prime }}\right)}^{2}\right)}^{⊤}\to {\left(v_{1}v_{2},{\left(v_{1}\right)}^{2}\right)}^{⊤}\;\mathrm{weakly}\;\mathrm{in}\;L^{2}\left(0,T;L^{2}\left(\Omega ;R^{2}\right)\right),\\ \; & {\left(\partial_{t}v_{1n^{\prime }},\partial_{t}v_{2n^{\prime }},\partial_{t}v_{3n^{\prime }},\partial_{t}v_{4n^{\prime }}\right)}^{⊤}\to {\left(\partial_{t}v_{1},\partial_{t}v_{2},\partial_{t}v_{3},\partial_{t}v_{4}\right)}^{⊤}\;\mathrm{weakly}\;\mathrm{in}\;L^{2}\left(0,T;H^{-1}\left(\Omega ;R^{4}\right)\right). \end{array}$$

Since the sequence ${\left\{{\left(v_{1n^{\prime }},v_{2n^{\prime }},v_{3n^{\prime }},v_{4n^{\prime }}\right)}^{⊤}\right\}}_{n^{\prime }\in N}$ satisfies the initial-boundary value problem (30)–(36), by Definition 3, for every quadruple ${\left(\varphi_{1},\varphi_{2},\varphi_{3},\varphi_{4}\right)}^{⊤}\in H^{1}\left(\Omega ;R^{4}\right)$, it holds that

(46) $$\begin{array}{cc} \; & \int_{\Omega }v_{1n^{\prime }}\left(x,t\right)\varphi_{1}\left(x\right)dx-\int_{\Omega }\sum_{\ell =0}^{n^{\prime }}\int_{\Omega }v_{1}^{0}\left(x\right)\psi_{1\ell }\left(x\right)dx\psi_{1\ell }\left(x\right)\varphi_{1}\left(x\right)dx\\ = & \int_{0}^{t}\int_{\Omega }\varphi_{1}\left(x\right)\left(\left(v_{2n^{\prime }}\left(x,s\right)-a\right)v_{1n^{\prime }}\left(x,s\right)+v_{3n^{\prime }}\left(x,s\right)+v_{4n^{\prime }}\left(x,s\right)\right)dxds\\ \; & -\int_{0}^{t}\int_{\Omega }D_{1}\left(x\right)\nabla^{⊤}v_{1n^{\prime }}\left(x,s\right)\nabla \varphi_{1}\left(x\right)dxds\;\mathrm{for}\;t\in \left[0,T\right],\\ \; & \int_{\Omega }v_{2n^{\prime }}\left(x,t\right)\varphi_{2}\left(x\right)dx-\int_{\Omega }\sum_{\ell =0}^{n^{\prime }}\int_{\Omega }v_{2}^{0}\left(x\right)\psi_{2\ell }\left(x\right)dx\psi_{2\ell }\left(x\right)\varphi_{2}\left(x\right)dx\\ = & \int_{0}^{t}\int_{\Omega }\varphi_{2}\left(x\right)\left(1-bv_{2n^{\prime }}\left(x,s\right)-{\left(v_{1n^{\prime }}\left(x,s\right)\right)}^{2}\right)dxds\\ \; & -\int_{0}^{t}\int_{\Omega }D_{2}\left(x\right)\nabla^{⊤}v_{2n^{\prime }}\left(x,s\right)\nabla \varphi_{2}\left(x\right)dxds\;\mathrm{for}\;t\in \left[0,T\right],\\ \; & \int_{\Omega }v_{3n^{\prime }}\left(x,t\right)\varphi_{3}\left(x\right)dx-\int_{\Omega }\sum_{\ell =0}^{n^{\prime }}\int_{\Omega }v_{3}^{0}\left(x\right)\psi_{3\ell }\left(x\right)dx\psi_{3\ell }\left(x\right)\varphi_{3}\left(x\right)dx\\ = & -\int_{0}^{t}\int_{\Omega }\varphi_{3}\left(x\right)\left(cv_{3n^{\prime }}\left(x,s\right)+v_{1n^{\prime }}\left(x,s\right)\right)dxds\\ \; & -\int_{0}^{t}\int_{\Omega }D_{3}\left(x\right)\nabla^{⊤}v_{3n^{\prime }}\left(x,s\right)\nabla \varphi_{3}\left(x\right)dxds\;\mathrm{for}\;t\in \left[0,T\right],\\ \; & \int_{\Omega }v_{4n^{\prime }}\left(x,t\right)\varphi_{4}\left(x\right)dx-\int_{\Omega }\sum_{\ell =0}^{n^{\prime }}\int_{\Omega }v_{4}^{0}\left(x\right)\psi_{4\ell }\left(x\right)dx\psi_{4\ell }\left(x\right)\varphi_{4}\left(x\right)dx\\ = & -\int_{0}^{t}\int_{\Omega }\varphi_{4}\left(x\right)\left(\alpha v_{4n^{\prime }}\left(x,s\right)+\beta v_{1n^{\prime }}\left(x,s\right)v_{2n^{\prime }}\left(x,s\right)\right)dxds\\ \; & -\int_{0}^{t}\int_{\Omega }D_{4}\left(x\right)\nabla^{⊤}v_{4n^{\prime }}\left(x,s\right)\nabla \varphi_{4}\left(x\right)dxds\;\mathrm{for}\;t\in \left[0,T\right]. \end{array}$$

Note that when $n^{\prime }$ tends to *∞*, it holds that

$$\begin{array}{cc} ( & \sum_{\ell =0}^{n^{\prime }}\int_{\Omega }v_{1}^{0}\left(x\right)\psi_{1\ell }\left(x\right)dx\psi_{1\ell },\sum_{\ell =0}^{n^{\prime }}\int_{\Omega }v_{2}^{0}\left(x\right)\psi_{2\ell }\left(x\right)dx\psi_{2\ell },\\ \; & \sum_{\ell =0}^{n^{\prime }}\int_{\Omega }v_{3}^{0}\left(x\right)\psi_{3\ell }\left(x\right)dx\psi_{3\ell },\sum_{\ell =0}^{n^{\prime }}\int_{\Omega }v_{4}^{0}\left(x\right)\psi_{4\ell }\left(x\right)dx\psi_{4\ell }{)}^{⊤}\\ \; & \to {\left(v_{1}^{0},v_{2}^{0},v_{3}^{0},v_{4}^{0}\right)}^{⊤}\;\mathrm{strongly}\;\mathrm{in}\;L^{2}\left(\Omega ;R^{4}\right), \end{array}$$

we derive from (41) by passing to the limit that the quadruple ${\left(v_{1},v_{2},v_{3},v_{4}\right)}^{⊤}$, given by (45), indeed satisfies (9) in Definition 3. That is, the quadruple ${\left(v_{1},v_{2},v_{3},v_{4}\right)}^{⊤}$ is a weak solution to the initial-boundary value problem (30)–(36), in the sense of Definition 3. This, together with Appendix A, implies that the proof of Theorem 1 is complete. □

**Theorem** **2.** *Suppose that Assumptions 1 and 2 hold true. For every quadruple ${\left(v_{1}^{0},v_{2}^{0},v_{3}^{0},v_{4}^{0}\right)}^{⊤}$ of initial data in the Hilbert space $L^{2}\left(\Omega ;R^{4}\right)$, the initial-boundary value problem (3)–(7) admits a unique weak solution*

$${\left(v_{1},v_{2},v_{3},v_{4}\right)}^{⊤}\in C\left(R^{+};L^{2}\left(\Omega ;R^{4}\right)\right)\cap L_{\mathrm{loc}}^{2}\left(R^{+};H^{1}\left(\Omega ;R^{4}\right)\right)$$

*in the sense of Definition 3. Moreover, the solution quadruple ${\left(v_{1},v_{2},v_{3},v_{4}\right)}^{⊤}$ automatically satisfies*

(47) $$\begin{array}{cccc} \; & \sum_{k=1}^{4}∥v_{k}{\left(\cdot ,t\right)∥}_{L^{2}\left(\Omega \right)}^{2}+2\sum_{k=1}^{4}\int_{0}^{t}∥\nabla v_{k}{\left(\cdot ,s\right)∥}_{L^{2}\left(\Omega ;R^{N}\right)}^{2}ds⩽M_{8}\sum_{k=1}^{4}{∥v_{k}^{0}∥}_{L^{2}\left(\Omega \right)}^{2}e^{M_{9}t}, & \; & t\in R^{+}, \end{array}$$

*with the positive constants $M_{8}$ and $M_{9}$ depending merely on a, b, c, α, β, Ω, $D_{k}$ (k=1,2,3,4), and independent of t and of $v_{k}$ (k=1,2,3,4). Moreover, for every T∈(0,+∞), the following map*

$$L^{2}\left(\Omega ;R^{4}\right)∋{\left(v_{1}^{0},v_{2}^{0},v_{3}^{0},v_{4}^{0}\right)}^{⊤}\mapsto {\left(v_{1},v_{2},v_{3},v_{4}\right)}^{⊤}\in C\left(\left[0,T\right];L^{2}\left(\Omega ;R^{4}\right)\right)$$

*is locally Lipschitz continuous.*

**Proof.**  The estimate (47), alongside with a standard continuation argument, implies immediately the global well-posedness of the initial-boundary value problem (3)–(7) and follows directly from (43) and (44). We choose to omit the detailed proof here. □

## 3. Existence Result of (5)–(8)–(48) and the Fixed-Time Synchronizability of the Drive-Response Systems (3) and (5) Controlled by (48)

In this section, our aim is to design for the response system (5) a control to enable the drive-response systems (3) and (5) to achieve fixed-time synchronization.

### 3.1. Design of the Synchronization Control

In this subsection, we are focused on finding the clue to designing a suitable control which would synchronize certainly, in a fixed time, the drive-response systems (3) and (5). Enlightened by the result in Lemma 3, we introduce the following control candidate:

(48) $$\begin{array}{cc} W_{1}\left(x,t\right)=\; & -(m_{11}+\frac{1}{2}|v_{1}\left(x,t\right)|+\frac{\beta +2}{2}|v_{2}\left(x,t\right)\left|\right)\left({\tilde{v}}_{1}\left(x,t\right)-v_{1}\left(x,t\right)\right)\\ & -m_{12}{\left(\sum_{k=1}^{4}{∥{\tilde{v}}_{k}\left(\cdot ,t\right)-v_{k}\left(\cdot ,t\right)∥}_{L^{2}\left(\Omega \right)}^{2}\right)}^{\mu -1}\left({\tilde{v}}_{1}\left(x,t\right)-v_{1}\left(x,t\right)\right)\\ & -m_{13}{\left|{\tilde{v}}_{1}\left(x,t\right)-v_{1}\left(x,t\right)\right|}^{2\gamma -2}\left({\tilde{v}}_{1}\left(x,t\right)-v_{1}\left(x,t\right)\right)\\ & -\frac{2\beta }{3}\left|{\tilde{v}}_{1}\left(x,t\right)-v_{1}\left(x,t\right)\right|\left({\tilde{v}}_{1}\left(x,t\right)-v_{1}\left(x,t\right)\right),\\ W_{2}\left(x,t\right)=\; & -(m_{21}+\frac{1+\beta }{2}|v_{1}\left(x,t\right)\left|\right)\left({\tilde{v}}_{2}\left(x,t\right)-v_{2}\left(x,t\right)\right)\\ & -m_{22}{\left(\sum_{k=1}^{4}{∥{\tilde{v}}_{k}\left(\cdot ,t\right)-v_{k}\left(\cdot ,t\right)∥}_{L^{2}\left(\Omega \right)}^{2}\right)}^{\mu -1}\left({\tilde{v}}_{2}\left(x,t\right)-v_{2}\left(x,t\right)\right)\\ & -m_{23}{\left|{\tilde{v}}_{2}\left(x,t\right)-v_{2}\left(x,t\right)\right|}^{2\gamma -2}\left({\tilde{v}}_{2}\left(x,t\right)-v_{2}\left(x,t\right)\right)\\ & -\frac{2\beta }{3}\left|{\tilde{v}}_{2}\left(x,t\right)-v_{2}\left(x,t\right)\right|\left({\tilde{v}}_{2}\left(x,t\right)-v_{2}\left(x,t\right)\right),\\ W_{3}\left(x,t\right)=\; & -m_{31}\left({\tilde{v}}_{3}\left(x,t\right)-v_{3}\left(x,t\right)\right)-m_{33}{\left|{\tilde{v}}_{3}\left(x,t\right)-v_{3}\left(x,t\right)\right|}^{2\gamma -2}\left({\tilde{v}}_{3}\left(x,t\right)-v_{3}\left(x,t\right)\right)\\ & -m_{32}{\left(\sum_{k=1}^{4}{∥{\tilde{v}}_{k}\left(\cdot ,t\right)-v_{k}\left(\cdot ,t\right)∥}_{L^{2}\left(\Omega \right)}^{2}\right)}^{\mu -1}\left({\tilde{v}}_{3}\left(x,t\right)-v_{3}\left(x,t\right)\right),\\ W_{4}\left(x,t\right)=\; & -(m_{41}+\frac{\beta }{2}|v_{1}\left(x,t\right)|+\frac{\beta }{2}|v_{2}\left(x,t\right)\left|\right)\left({\tilde{v}}_{4}\left(x,t\right)-v_{4}\left(x,t\right)\right)\\ & -m_{42}{\left(\sum_{k=1}^{4}{∥{\tilde{v}}_{k}\left(\cdot ,t\right)-v_{k}\left(\cdot ,t\right)∥}_{L^{2}\left(\Omega \right)}^{2}\right)}^{\mu -1}\left({\tilde{v}}_{4}\left(x,t\right)-v_{4}\left(x,t\right)\right)\\ & -m_{43}{\left|{\tilde{v}}_{4}\left(x,t\right)-v_{4}\left(x,t\right)\right|}^{2\gamma -2}\left({\tilde{v}}_{4}\left(x,t\right)-v_{4}\left(x,t\right)\right)\\ & -\frac{2\beta }{3}\left|{\tilde{v}}_{4}\left(x,t\right)-v_{4}\left(x,t\right)\right|\left({\tilde{v}}_{4}\left(x,t\right)-v_{4}\left(x,t\right)\right), \end{array}$$

where the parameter $m_{kh}$ is to be determined later, k=1,2,3,4, h=1,2,3, the parameter μ can take any value in the interval (0,1), and the parameter γ can take any value in the interval (1,+∞). It is not difficult to observe that the synchronization problem for the financial systems (3) and (5) is equivalent to the stabilization problem for the error system

(49) $$\left\{\begin{array}{cccc} \partial_{t}w_{1}= & \mathrm{div}\left(D_{1}\nabla w_{1}\right)+\left(w_{2}+v_{2}-a\right)w_{1}+v_{1}w_{2} & \; & \\ \; & +w_{3}+w_{4}+W_{1} & \; & \mathrm{in}\;\Omega \times R^{+},\\ \partial_{t}w_{2}= & \mathrm{div}\left(D_{2}\nabla w_{2}\right)-bw_{2}-\left(w_{1}+2v_{1}\right)w_{1}+W_{2} & \; & \mathrm{in}\;\Omega \times R^{+},\\ \partial_{t}w_{3}= & \mathrm{div}\left(D_{3}\nabla w_{3}\right)-cw_{3}-w_{1}+W_{3} & \; & \mathrm{in}\;\Omega \times R^{+},\\ \partial_{t}w_{4}= & \mathrm{div}\left(D_{4}\nabla w_{4}\right)-\alpha w_{4}-\beta w_{1}w_{2}-\beta v_{2}w_{1}\; & \; & \\ \; & -\beta v_{1}w_{2}+W_{4} & \; & \mathrm{in}\;\Omega \times R^{+},\\ \partial_{\nu }w_{1}= & \partial_{\nu }w_{2}=\partial_{\nu }w_{3}=\partial_{\nu }w_{4}=0 & \; & \mathrm{on}\;\partial \Omega \times R^{+} \end{array}\right.$$

where the new unknowns $w_{k}$ is given by

(50) $$\begin{array}{cccc} \; & w_{k}\left(x,t\right)={\tilde{v}}_{k}\left(x,t\right)-v_{k}\left(x,t\right), & \; & k=1,2,3,4. \end{array}$$

Plug (48) and (50) into (49), to obtain

(51) $$\left\{\begin{array}{cccc} \partial_{t}w_{1}= & \;\mathrm{div}\left(D_{1}\nabla w_{1}\right)+\left(w_{2}+v_{2}-a\right)w_{1}+v_{1}w_{2}+w_{3}+w_{4} & \; & \\ \; & -\frac{2\beta }{3}\left|w_{1}\right|w_{1}-m_{12}{\left(\sum_{k=1}^{4}{∥w_{k}\left(\cdot ,t\right)∥}_{L^{2}\left(\Omega \right)}^{2}\right)}^{\mu -1}w_{1}\; & \; & \\ \; & -m_{13}|w_{1}{|}^{2\gamma -2}w_{1}-(m_{11}+\frac{1}{2}|v_{1}|+\frac{\beta +2}{2}|v_{2}\left|\right)w_{1} & \; & \mathrm{in}\;\Omega \times R^{+},\\ \partial_{t}w_{2}= & \;\mathrm{div}\left(D_{2}\nabla w_{2}\right)-bw_{2}-\left(w_{1}+2v_{1}\right)w_{1}\; & \; & \\ \; & -\frac{2\beta }{3}\left|w_{2}\right|w_{2}-m_{22}{\left(\sum_{k=1}^{4}{∥w_{k}\left(\cdot ,t\right)∥}_{L^{2}\left(\Omega \right)}^{2}\right)}^{\mu -1}w_{2}\; & \; & \\ \; & -m_{23}|w_{2}{|}^{2\gamma -2}w_{2}-(m_{21}+\frac{1+\beta }{2}|v_{1}\left|\right)w_{2} & \; & \mathrm{in}\;\Omega \times R^{+},\\ \partial_{t}w_{3}= & \;\mathrm{div}\left(D_{3}\nabla w_{3}\right)-cw_{3}-w_{1}-m_{31}w_{3}\; & \; & \\ \; & -m_{32}{\left(\sum_{k=1}^{4}{∥w_{k}\left(\cdot ,t\right)∥}_{L^{2}\left(\Omega \right)}^{2}\right)}^{\mu -1}w_{3}-m_{33}{\left|w_{3}\right|}^{2\gamma -2}w_{3}\; & \; & \mathrm{in}\;\Omega \times R^{+},\\ \partial_{t}w_{4}= & \;\mathrm{div}\left(D_{4}\nabla w_{4}\right)-\alpha w_{4}-\beta w_{1}w_{2}-\beta v_{2}w_{1}-\beta v_{1}w_{2} & \; & \\ \; & -\frac{2\beta }{3}\left|w_{4}\right|w_{4}-m_{42}{\left(\sum_{k=1}^{4}{∥w_{k}\left(\cdot ,t\right)∥}_{L^{2}\left(\Omega \right)}^{2}\right)}^{\mu -1}w_{4}\; & \; & \\ \; & -m_{43}|w_{4}{|}^{2\gamma -2}w_{4}-(m_{41}+\frac{\beta }{2}|v_{1}|+\frac{\beta }{2}|v_{2}\left|\right)w_{4} & \; & \mathrm{in}\;\Omega \times R^{+},\\ \partial_{\nu }w_{1}= & \partial_{\nu }w_{2}=\partial_{\nu }w_{3}=\partial_{\nu }w_{4}=0 & \; & \mathrm{on}\;\partial \Omega \times R^{+}. \end{array}\right.$$

Obviously, $w_{k}$ (k=1,2,3,4) given by (50) satisfies the initial condition

(52) $$\left\{\begin{array}{ccc} w_{1}\left(\cdot ,0\right)={\tilde{v}}_{1}^{0}-v_{1}^{0} & \; & \mathrm{in}\;\Omega ,\\ w_{2}\left(\cdot ,0\right)={\tilde{v}}_{2}^{0}-v_{2}^{0} & \; & \mathrm{in}\;\Omega ,\\ w_{3}\left(\cdot ,0\right)={\tilde{v}}_{3}^{0}-v_{3}^{0} & \; & \mathrm{in}\;\Omega ,\\ w_{4}\left(\cdot ,0\right)={\tilde{v}}_{4}^{0}-v_{4}^{0} & \; & \mathrm{in}\;\Omega . \end{array}\right.$$

We shall establish a stabilization criterion for the nonlinear system (51) with variable coefficients and, in the meantime, provide two fixed-time synchronization criteria for the drive financial system (3) and the response system (5) upon which the control (48) is implemented.

### 3.2. Global Existence of the Problems (51) and (5)–(48)

In view of Theorem 2 and its proof, we give first the following global existence and uniqueness for the initial-boundary value problem (51) and (52).

**Theorem** **3.** *Suppose that Assumptions 1 and 2 hold true. Assume that $m_{kh}\in \left(0,+\infty \right)$, k=1,2,3,4, h=2,3, and that $m_{k1}\in R$, k=1,2,3,4. For every quadruple ${\left(v_{1}^{0},v_{2}^{0},v_{3}^{0},v_{4}^{0}\right)}^{⊤}$ and every quadruple ${\left({\tilde{v}}_{1}^{0},{\tilde{v}}_{2}^{0},{\tilde{v}}_{3}^{0},{\tilde{v}}_{4}^{0}\right)}^{⊤}$ of initial data in the Hilbert space $L^{2}\left(\Omega ;R^{4}\right)$, the initial-boundary value problem (51) and (52) admits a unique weak solution*

$${\left(w_{1},w_{2},w_{3},w_{4}\right)}^{⊤}\in C\left(R^{+};L^{2}\left(\Omega ;R^{4}\right)\right)\cap L_{\mathrm{loc}}^{2}\left(R^{+};H^{1}\left(\Omega ;R^{4}\right)\right)$$

*in a similar sense as that in Definition 3. Moreover, ${\left(w_{1},w_{2},w_{3},w_{4}\right)}^{⊤}$ satisfies automatically*

(53) $$\begin{array}{cccc} \; & \sum_{k=1}^{4}∥w_{k}{\left(\cdot ,t\right)∥}_{L^{2}\left(\Omega \right)}^{2}+2\sum_{k=1}^{4}\int_{0}^{t}{∥\nabla w_{k}\left(\cdot ,s\right)∥}_{L^{2}\left(\Omega ;R^{N}\right)}^{2}ds & \; & \\ \; & +2\sum_{k=1}^{4}\int_{0}^{t}\int_{\Omega }{\left|w_{k}\left(x,s\right)\right|}^{2\lambda }dxds & \; & \\ \; & +2\int_{0}^{t}\int_{\Omega }\left(|v_{1}\left(x,s\right)|+|v_{2}\left(x,s\right)|\right){\left|w_{1}\left(x,s\right)\right|}^{2}dxds & \; & \\ \; & +2\int_{0}^{t}\int_{\Omega }|v_{1}\left(x,s\right)||w_{2}{\left(x,s\right)|}^{2}dxds & \; & \\ \; & +2\int_{0}^{t}\int_{\Omega }\left(|v_{1}\left(x,s\right)|+|v_{2}\left(x,s\right)|\right){\left|w_{4}\left(x,s\right)\right|}^{2}dxds & \; & \\ \; & +\frac{2\beta }{3}\int_{0}^{t}\int_{\Omega }\left(|w_{1}{\left(x,s\right)|}^{3}+|w_{2}{\left(x,s\right)|}^{3}+{\left|w_{4}\left(x,s\right)\right|}^{3}\right)dxds\; & \; & \\ ⩽\; & M_{10}e^{M_{11}\int_{0}^{t}(1+∥v_{1}{\left(\cdot ,s\right)∥}_{H^{1}\left(\Omega \right)}+∥v_{2}\left(\cdot ,s\right){∥}_{H^{1}\left(\Omega \right)})ds}\sum_{k=1}^{4}{∥{\tilde{v}}_{k}^{0}-v_{k}^{0}∥}_{L^{2}\left(\Omega \right)}^{2}, & \; & t\in R^{+}, \end{array}$$

*where the quadruple ${\left(v_{1},v_{2},v_{3},v_{4}\right)}^{⊤}$ is the unique solution to the initial-boundary value problem (51) corresponding to the initial data ${\left(v_{1}^{0},v_{2}^{0},v_{3}^{0},v_{4}^{0}\right)}^{⊤}$ (see Theorem 2), the positive constants $M_{10}$ and $M_{11}$ depend on a, b, c, α, β, Ω, $D_{k}$ (k=1,2,3,4) and $m_{kh}$ (k=1,2,3,4, h=1,2,3), but are independent of t, $w_{k}$, and $v_{k}$.*

**Proof.**  The existence and uniqueness can be proved by applying Theorems 1 and 2, and the idea in the proof of Theorem 1. We choose to omit the detailed proof of the existence and uniqueness parts of the proof of Theorem 3.

Now, let us begin proving the estimate (53). Mimicking the proof of Theorem 1 (see (35) and (24), in particular), we come up with the following auxiliary functional for every solution quadruple ${\left(v_{1}^{0},v_{2}^{0},v_{3}^{0},v_{4}^{0}\right)}^{⊤}$ for the initial-boundary value problem (51) and (52):

(54) $$\begin{array}{cc} \tilde{E}\left(t\right)= & \sum_{k=1}^{4}\int_{\Omega }|w_{k}{\left(x,t\right)|}^{2}dx+2\sum_{k=1}^{4}\int_{0}^{t}\int_{\Omega }D_{k}\left(x\right){\left|\nabla w_{k}\left(x,s\right)\right|}^{2}dxds\\ \; & +2\sum_{k=1}^{4}m_{k3}\int_{0}^{t}\int_{\Omega }{\left|w_{k}\left(x,s\right)\right|}^{2\lambda }dxds\\ \; & +\frac{2\beta }{3}\int_{0}^{t}\int_{\Omega }\left(|w_{1}{\left(x,s\right)|}^{3}+|w_{2}{\left(x,s\right)|}^{3}+{\left|w_{4}\left(x,s\right)\right|}^{3}\right)dxds\\ \; & +2\int_{0}^{t}\int_{\Omega }\left(a|w_{1}{\left(x,s\right)|}^{2}+b|w_{2}{\left(x,s\right)|}^{2}+c|w_{3}{\left(x,s\right)|}^{2}+\alpha {\left|w_{4}\left(x,s\right)\right|}^{2}\right)dxds\\ \; & +\int_{0}^{t}\int_{\Omega }\left(|v_{1}\left(x,s\right)|+\left(\beta +2\right)|v_{2}\left(x,s\right)|\right){\left|w_{1}\left(x,s\right)\right|}^{2}dxds\\ \; & +\left(1+\beta \right)\int_{0}^{t}\int_{\Omega }|v_{1}\left(x,s\right)||w_{2}{\left(x,s\right)|}^{2}dxds\\ \; & +\beta \int_{0}^{t}\int_{\Omega }\left(|v_{1}\left(x,s\right)|+|v_{2}\left(x,s\right)|\right){\left|w_{4}\left(x,s\right)\right|}^{2}dxds,\;t\in R^{+}. \end{array}$$

Mimicking the steps in (37), we have

$$\begin{array}{cc} D^{+}\tilde{E}\left(t\right)= & \;2\sum_{k=1}^{4}\int_{\Omega }w_{k}\left(x,t\right)\partial_{t}w_{k}\left(x,t\right)dx+2\sum_{k=1}^{4}\int_{\Omega }D_{k}\left(x\right){\left|\nabla w_{k}\left(x,t\right)\right|}^{2}\;dx\\ \; & \;+2\sum_{k=1}^{4}m_{k3}\int_{\Omega }|w_{k}{\left(x,t\right)|}^{2\lambda }dx+\left(1+\beta \right)\int_{\Omega }|v_{1}\left(x,t\right)||w_{2}{\left(x,t\right)|}^{2}\;dx\\ \; & +\frac{2\beta }{3}\int_{\Omega }\left(|w_{1}{\left(x,t\right)|}^{3}+|w_{2}{\left(x,t\right)|}^{3}+{\left|w_{4}\left(x,t\right)\right|}^{3}\right)dx\\ \; & +2\int_{\Omega }\left(a|w_{1}{\left(x,t\right)|}^{2}+b|w_{2}{\left(x,t\right)|}^{2}+c|w_{3}{\left(x,t\right)|}^{2}+\alpha {\left|w_{4}\left(x,t\right)\right|}^{2}\right)dx\\ \; & +\int_{\Omega }\left(|v_{1}\left(x,s\right)|+\left(\beta +2\right)|v_{2}\left(x,t\right)|\right){\left|w_{1}\left(x,t\right)\right|}^{2}\;dx\\ \; & +\beta \int_{\Omega }\left(|v_{1}\left(x,t\right)|+|v_{2}\left(x,t\right)|\right){\left|w_{4}\left(x,t\right)\right|}^{2}\;dx\\ = & \int_{\Omega }\left(2v_{2}\left(x,t\right)-2a-2m_{11}-|v_{1}\left(x,t\right)|-\left(\beta +2\right)|v_{2}\left(x,t\right)|\right){\left|w_{1}\left(x,t\right)\right|}^{2}dx\\ \; & \;+2\int_{\Omega }\left(w_{3}\left(x,t\right)+w_{4}\left(x,t\right)-v_{1}\left(x,t\right)w_{2}\left(x,t\right)\right)w_{1}\left(x,t\right)dx\\ \; & -2\int_{\Omega }D_{1}\left(x\right)|\nabla w_{1}{\left(x,t\right)|}^{2}dx-\frac{4\beta }{3}\int_{\Omega }{\left|w_{1}\left(x,t\right)\right|}^{3}dx\\ \; & -2m_{12}{\left(\sum_{k=1}^{4}{∥w_{k}\left(\cdot ,t\right)∥}_{L^{2}\left(\Omega \right)}^{2}\right)}^{\mu -1}∥w_{1}{\left(\cdot ,t\right)∥}_{L^{2}\left(\Omega \right)}^{2}-2m_{13}\int_{\Omega }{\left|w_{1}\left(x,t\right)\right|}^{2\gamma }dx\\ \; & -\int_{\Omega }\left(2b+2m_{21}+\left(1+\beta \right)\left|v_{1}\left(x,t\right)\right|\right){\left|w_{2}\left(x,t\right)\right|}^{2}dx\\ \; & -\frac{4\beta }{3}\int_{\Omega }|w_{2}{\left(x,t\right)|}^{3}dx-2m_{22}{\left(\sum_{k=1}^{4}{∥w_{k}\left(\cdot ,t\right)∥}_{L^{2}\left(\Omega \right)}^{2}\right)}^{\mu -1}{∥w_{2}\left(\cdot ,t\right)∥}_{L^{2}\left(\Omega \right)}^{2}\\ \; & -2\int_{\Omega }D_{2}\left(x\right)|\nabla w_{2}{\left(x,t\right)|}^{2}dx-2m_{23}\int_{\Omega }{\left|w_{2}\left(x,t\right)\right|}^{2\gamma }dx\\ \; & -2\left(c+m_{31}\right)\int_{\Omega }{\left|w_{3}\left(x,t\right)\right|}^{2}dx-2\int_{\Omega }w_{1}\left(x,t\right)w_{3}\left(x,t\right)dx\\ \; & -2m_{32}{\left(\sum_{k=1}^{4}{∥w_{k}\left(\cdot ,t\right)∥}_{L^{2}\left(\Omega \right)}^{2}\right)}^{\mu -1}{∥w_{3}\left(\cdot ,t\right)∥}_{L^{2}\left(\Omega \right)}^{2}\\ \; & -2m_{33}\int_{\Omega }|w_{3}{\left(x,t\right)|}^{2\gamma }dx-2\int_{\Omega }D_{3}\left(x\right){\left|\nabla w_{3}\left(x,t\right)\right|}^{2}\;dx\\ \; & -\int_{\Omega }\left(2\alpha +2m_{41}+\beta |v_{1}\left(x,t\right)|+\beta |v_{2}\left(x,t\right)|\right){\left|w_{4}\left(x,t\right)\right|}^{2}dx\\ \; & -2\beta \int_{\Omega }\left(w_{1}\left(x,t\right)w_{2}\left(x,t\right)+v_{2}\left(x,t\right)w_{1}\left(x,t\right)+v_{1}\left(x,t\right)w_{2}\left(x,t\right)\right)w_{4}\left(x,t\right)dx\\ \; & -\frac{4\beta }{3}\int_{\Omega }|w_{4}{\left(x,t\right)|}^{3}dx-2m_{42}{\left(\sum_{k=1}^{4}{∥w_{k}\left(\cdot ,t\right)∥}_{L^{2}\left(\Omega \right)}^{2}\right)}^{\mu -1}{∥w_{4}\left(\cdot ,t\right)∥}_{L^{2}\left(\Omega \right)}^{2}\\ \; & -2m_{43}\int_{\Omega }|w_{4}{\left(x,t\right)|}^{2\gamma }dx-2\int_{\Omega }D_{4}\left(x\right){\left|\nabla w_{4}\left(x,t\right)\right|}^{2}dx\\ \; & +2\sum_{k=1}^{4}\int_{\Omega }D_{k}\left(x\right)|\nabla w_{k}{\left(x,t\right)|}^{2}dx+2\sum_{k=1}^{4}m_{k3}\int_{\Omega }{\left|w_{k}\left(x,t\right)\right|}^{2\lambda }dx\\ \; & +\frac{2\beta }{3}\int_{\Omega }\left(|w_{1}{\left(x,t\right)|}^{3}+|w_{2}{\left(x,t\right)|}^{3}+{\left|w_{4}\left(x,t\right)\right|}^{3}\right)dx\\ \; & +2\int_{\Omega }\left(a|w_{1}{\left(x,t\right)|}^{2}+b|w_{2}{\left(x,t\right)|}^{2}+c|w_{3}{\left(x,t\right)|}^{2}+\alpha {\left|w_{4}\left(x,t\right)\right|}^{2}\right)dx\\ \; & +\int_{\Omega }\left(|v_{1}\left(x,t\right)|+\left(\beta +2\right)|v_{2}\left(x,t\right)|\right){\left|w_{1}\left(x,t\right)\right|}^{2}\;dx\\ \; & +\left(1+\beta \right)\int_{\Omega }|v_{1}\left(x,t\right)||w_{2}{\left(x,t\right)|}^{2}dx+\beta \int_{\Omega }\left(|v_{1}\left(x,t\right)|+|v_{2}\left(x,t\right)|\right){\left|w_{4}\left(x,t\right)\right|}^{2}dx \end{array}$$

(55) $$\begin{array}{cc} = & 2\int_{\Omega }\left(v_{2}\left(x,t\right)-m_{11}\right){\left|w_{1}\left(x,t\right)\right|}^{2}dx-2\int_{\Omega }v_{1}\left(x,t\right)w_{1}\left(x,t\right)w_{2}\left(x,t\right)dx\\ \; & +2\int_{\Omega }\left(1-\beta v_{2}\left(x,t\right)\right)w_{1}\left(x,t\right)w_{4}\left(x,t\right)dx-2m_{21}\int_{\Omega }{\left|w_{2}\left(x,t\right)\right|}^{2}dx\\ \; & -2m_{31}\int_{\Omega }|w_{3}{\left(x,t\right)|}^{2}dx-2m_{41}\int_{\Omega }{\left|w_{4}\left(x,t\right)\right|}^{2}dx-2\beta \int_{\Omega }v_{1}\left(x,t\right)w_{2}\left(x,t\right)w_{4}\left(x,t\right)dx\\ \; & -\frac{2\beta }{3}\int_{\Omega }\left(|w_{1}{\left(x,t\right)|}^{3}+|w_{2}{\left(x,t\right)|}^{3}+{\left|w_{4}\left(x,t\right)\right|}^{3}\right)dx\\ \; & -2\beta \int_{\Omega }w_{1}\left(x,t\right)w_{2}\left(x,t\right)w_{4}\left(x,t\right)dx\\ \; & -2{\left(\sum_{k=1}^{4}{∥w_{k}\left(\cdot ,t\right)∥}_{L^{2}\left(\Omega \right)}^{2}\right)}^{\mu -1}\sum_{k=1}^{4}m_{k2}{∥w_{k}\left(\cdot ,t\right)∥}_{L^{2}\left(\Omega \right)}^{2}⩽g\left(t\right)\tilde{E}\left(t\right),\;t\in R^{+}, \end{array}$$

which, together with Gronwall’s Lemma, implies directly

(56) $$\begin{array}{cc} \tilde{E}\left(t\right)⩽\; & e^{\int_{0}^{t}g\left(s\right)ds}\tilde{E}\left(0\right)\\ =\; & e^{\int_{0}^{t}g\left(s\right)ds}\sum_{k=1}^{4}{∥{\tilde{v}}_{k}^{0}-v_{k}^{0}∥}_{L^{2}\left(\Omega \right)}^{2},\;t\in R^{+}, \end{array}$$

where the function g(t) is given by

$$\begin{array}{cc} g(t)=\max(\; & 1+2|m_{11}|+∥v_{1}{\left(\cdot ,t\right)∥}_{H^{1}\left(\Omega \right)}+\left(2+\beta \right){∥v_{2}\left(\cdot ,t\right)∥}_{H^{1}\left(\Omega \right)},\\ \; & 2|m_{21}|+\left(1+\beta \right)∥v_{1}{\left(\cdot ,t\right)∥}_{H^{1}\left(\Omega \right)},2\left|m_{31}\right|,\\ \; & 1+2|m_{41}|+\beta ∥v_{1}{\left(\cdot ,t\right)∥}_{H^{1}\left(\Omega \right)}+\beta {∥v_{2}\left(\cdot ,t\right)∥}_{H^{1}\left(\Omega \right)}),\;t\in R^{+}. \end{array}$$

This, together with (54) and (56), implies immediately that the estimate (53) in Theorem 3 on solutions to the initial-boundary value problem (51) and (52) holds true. □

Enlightened by Theorem 3, we have the following global existence and uniqueness for the initial-boundary value problem (5)–(8)–(48).

**Theorem** **4.** *Suppose that Assumptions 1 and 2 hold true. Assume that $m_{kh}\in \left(0,+\infty \right)$, k=1,2,3,4, h=2,3, and that $m_{k1}\in R$, k=1,2,3,4. For every quadruple ${\left(v_{1}^{0},v_{2}^{0},v_{3}^{0},v_{4}^{0}\right)}^{⊤}$ and every quadruple ${\left({\tilde{v}}_{1}^{0},{\tilde{v}}_{2}^{0},{\tilde{v}}_{3}^{0},{\tilde{v}}_{4}^{0}\right)}^{⊤}$ of initial data in the Hilbert space $L^{2}\left(\Omega ;R^{4}\right)$, the initial-boundary value problem (5)–(8)–(48) admits a unique weak solution*

$${\left({\tilde{v}}_{1},{\tilde{v}}_{2},{\tilde{v}}_{3},{\tilde{v}}_{4}\right)}^{⊤}\in C\left(R^{+};L^{2}\left(\Omega ;R^{4}\right)\right)\cap L_{\mathrm{loc}}^{2}\left(R^{+};H^{1}\left(\Omega ;R^{4}\right)\right)$$

*in a similar sense as that in Definition 3. Moreover, ${\left({\tilde{v}}_{1},{\tilde{v}}_{2},{\tilde{v}}_{3},{\tilde{v}}_{4}\right)}^{⊤}$ satisfies automatically*

$$\begin{array}{cc} \; & \sum_{k=1}^{4}∥{\tilde{v}}_{k}\left(\cdot ,t\right)-v_{k}{\left(\cdot ,t\right)∥}_{L^{2}\left(\Omega \right)}^{2}+2\sum_{k=1}^{4}\int_{0}^{t}{∥\nabla {\tilde{v}}_{k}\left(\cdot ,s\right)-\nabla v_{k}\left(\cdot ,s\right)∥}_{L^{2}\left(\Omega ;R^{N}\right)}^{2}\;ds\\ \; & +2\sum_{k=1}^{4}\int_{0}^{t}\int_{\Omega }{\left|{\tilde{v}}_{k}\left(x,s\right)-v_{k}\left(x,s\right)\right|}^{2\lambda }dx\;ds\\ \; & +2\int_{0}^{t}\int_{\Omega }\left(|v_{1}\left(x,s\right)|+|v_{2}\left(x,s\right)|\right){\left|{\tilde{v}}_{1}\left(x,s\right)-v_{1}\left(x,s\right)\right|}^{2}dx\;ds\\ \; & +2\int_{0}^{t}\int_{\Omega }|v_{1}\left(x,s\right)||{\tilde{v}}_{2}\left(x,s\right)-v_{2}{\left(x,s\right)|}^{2}dx\;ds\\ \; & +2\int_{0}^{t}\int_{\Omega }\left(|v_{1}\left(x,s\right)|+|v_{2}\left(x,s\right)|\right){\left|{\tilde{v}}_{4}\left(x,s\right)-v_{4}\left(x,s\right)\right|}^{2}dx\;ds\\ \; & +\frac{2\beta }{3}\int_{0}^{t}\int_{\Omega }\left(|{\tilde{v}}_{1}\left(x,s\right)-v_{1}{\left(x,s\right)|}^{3}+|{\tilde{v}}_{2}\left(x,s\right)-v_{2}{\left(x,s\right)|}^{3}+{\left|{\tilde{v}}_{4}\left(x,s\right)-v_{4}\left(x,s\right)\right|}^{3}\right)dxds\\ ⩽\; & M_{10}e^{M_{11}\int_{0}^{t}(1+∥v_{1}{\left(\cdot ,s\right)∥}_{H^{1}\left(\Omega \right)}+∥v_{2}\left(\cdot ,s\right){∥}_{H^{1}\left(\Omega \right)})ds}\sum_{k=1}^{4}{∥{\tilde{v}}_{k}^{0}-v_{k}^{0}∥}_{L^{2}\left(\Omega \right)}^{2},\;t\in R^{+}, \end{array}$$

*where the quadruple ${\left(v_{1},v_{2},v_{3},v_{4}\right)}^{⊤}$ is the unique solution to the initial boundary value problem (51) corresponding to the initial data ${\left(v_{1}^{0},v_{2}^{0},v_{3}^{0},v_{4}^{0}\right)}^{⊤}$ (see Theorem 2), the positive constants $M_{10}$ and $M_{11}$, given as in Theorem 3, depend on a, b, c, α, β, Ω, $D_{k}$ (k=1,2,3,4), $m_{kh}$ (k=1,2,3,4, h=1,2,3), but are independent of t, ${\tilde{v}}_{k}$ and $v_{k}$.*

**Proof.**  Theorem 4 is simply a reformulation of Theorem 3; therefore, the proof of Theorem 4 is omitted here. □

**Remark** **6.** *By applying mainly Hölder’s inequality, we can prove*

$$\begin{array}{cc} \sum_{k=1}^{4}\int_{0}^{t}\int_{\Omega }{\left|w_{k}\left(x,s\right)\right|}^{2\mu }dxds⩽ & \;{\left(t\mathrm{meas}\Omega \right)}^{1-\frac{\mu }{\lambda }}\sum_{k=1}^{4}{\left(\int_{0}^{t}\int_{\Omega }{\left|w_{k}\left(x,s\right)\right|}^{2\lambda }dxds\right)}^{\frac{\mu }{\lambda }}\\ ⩽ & \;{\left(t\mathrm{meas}\Omega \right)}^{1-\frac{\mu }{\lambda }}{\left(\sum_{k=1}^{4}\int_{0}^{t}\int_{\Omega }{\left|w_{k}\left(x,s\right)\right|}^{2\lambda }dxds\right)}^{\frac{\mu }{\lambda }}. \end{array}$$

*Therefore, we chose to omit the term*

$$\sum_{k=1}^{4}\int_{0}^{t}\int_{\Omega }{\left|w_{k}\left(x,s\right)\right|}^{2\mu }dxdsds$$

*in the estimate of ${\left(w_{1},w_{2},w_{3},w_{4}\right)}^{⊤}$ in Theorem 3. Similarly, we have*

$$\begin{array}{cc} \; & \sum_{k=1}^{4}\int_{0}^{t}\int_{\Omega }{\left|{\tilde{v}}_{k}\left(x,s\right)-v_{k}\left(x,s\right)\right|}^{2\mu }dx\;ds\\ ⩽ & \;{\left(t\mathrm{meas}\Omega \right)}^{1-\frac{\mu }{\lambda }}{\left(\sum_{k=1}^{4}\int_{0}^{t}\int_{\Omega }{\left|{\tilde{v}}_{k}\left(x,s\right)-v_{k}\left(x,s\right)\right|}^{2\lambda }dxds\right)}^{\frac{\mu }{\lambda }}, \end{array}$$

*therefore, we chose to omit the term*

$$\sum_{k=1}^{4}\int_{0}^{t}\int_{\Omega }{\left|{\tilde{v}}_{k}\left(x,s\right)-v_{k}\left(x,s\right)\right|}^{2\mu }dxds$$

*in the estimate of ${\left({\tilde{v}}_{1},{\tilde{v}}_{2},{\tilde{v}}_{3},{\tilde{v}}_{4}\right)}^{⊤}$ in Theorem 4.*

### 3.3. The Fixed-Time Synchronizability of the Drive-Response Systems (3) and (5) Controlled by (48)

**Theorem** **5.** *Suppose that Assumptions 1 and 2 hold true. Assume that $m_{kh}\in \left(0,+\infty \right)$, k=1,2,3,4, h=2,3, and that $m_{k1}\in R^{+}$ (k=1,2,3,4) render the matrix*

$$\left(\begin{array}{cccc} m_{11}+a & 0 & 0 & -\frac{1}{2}\\ 0 & m_{21}+b\; & 0 & 0\\ 0 & 0 & m_{31}+c & 0\\ -\frac{1}{2} & 0 & 0 & m_{31}+\alpha \end{array}\right)$$

*to be semi-positive definite. For every pair (μ,γ), with 0<μ<1<γ, there exists a*

(57) $$\begin{array}{cc} T_{0}⩽\; & \frac{1}{2(1-\mu )}\max\left(\frac{1}{m_{12}},\frac{1}{m_{22}},\frac{1}{m_{32}},\frac{1}{m_{42}}\right)\\ \; & +\frac{{\left(4\mathrm{meas}\Omega \right)}^{\gamma -1}}{2(\gamma -1)}\max\left(\frac{1}{m_{13}},\frac{1}{m_{23}},\frac{1}{m_{33}},\frac{1}{m_{43}}\right), \end{array}$$

*such that for every quadruple ${\left(v_{1}^{0},v_{2}^{0},v_{3}^{0},v_{4}^{0}\right)}^{⊤}$ and every quadruple ${\left({\tilde{v}}_{1}^{0},{\tilde{v}}_{2}^{0},{\tilde{v}}_{3}^{0},{\tilde{v}}_{4}^{0}\right)}^{⊤}$ of initial data in the Hilbert space $L^{2}\left(\Omega ;R^{4}\right)$, the solution quadruple ${\left(v_{1},v_{2},v_{3},v_{4}\right)}^{⊤}$ to the initial-boundary value problem (3)–(7) and the solution quadruple ${\left({\tilde{v}}_{1},{\tilde{v}}_{2},{\tilde{v}}_{3},{\tilde{v}}_{4}\right)}^{⊤}$ to the initial-boundary value problem (5)–(8)–(48) satisfy*

(58) $$\begin{array}{cccc} \; & \sum_{k=1}^{4}{∥{\tilde{v}}_{k}\left(\cdot ,t\right)-v_{k}\left(\cdot ,t\right)∥}_{L^{2}\left(\Omega \right)}^{2}=0, & \; & t\in [T_{0},+\infty ). \end{array}$$

**Lemma** **3** ([41,42]). *Let $V:R^{+}\to R^{+}$ be a continuous function. If there exists a quadruple ${\left(\mu ,\delta ,\gamma ,\gamma \right)}^{⊤}\in {\left(R^{+}\right)}^{4}$, with λ>0, μ>0 and 0<μ<1<γ, such that*

$$\begin{array}{cccc} \; & D^{+}V\left(t\right)⩽-\lambda V^{\mu }\left(t\right)-\delta V^{\gamma }\left(t\right),\; & \; & t\in R^{+} \end{array}$$

*then there exists a $T_{0}>0$ (the settling time) with*

$$T_{0}⩽\frac{1}{\lambda (1-\mu )}+\frac{1}{\delta (\gamma -1)}$$

*such that*

$$\begin{array}{cccc} \; & V(t)\equiv 0,\; & \; & t\in [T_{0},+\infty ). \end{array}$$

**Proof.**  As pointed in Section 3.1, the proof of Theorem 5 boils down to proving fixed-time stabilizability of the controlled error system (51). To every solution quadruple ${\left(w_{1},w_{2},w_{3},w_{4}\right)}^{⊤}$ to the initial-boundary value problem (51) and (52), we associate the following functional

(59) $$\begin{array}{cccc} \; & V\left(t\right)=\int_{\Omega }\sum_{k=1}^{4}|w_{k}{\left(x,t\right)|}^{2}dx=\sum_{k=1}^{4}{∥w_{k}\left(\cdot ,t\right)∥}_{L^{2}\left(\Omega \right)}^{2} & \; & \mathrm{for}\;t\in R^{+}. \end{array}$$

Taking similar steps as in (37) and (55), we have

$$\begin{array}{cc} D^{+}V\left(t\right)=\; & 2\sum_{k=1}^{4}\int_{\Omega }w_{k}\left(x,t\right)\partial_{t}w_{k}\left(x,t\right)dx\\ =\; & 2\int_{\Omega }\left(v_{2}\left(x,t\right)-\left|v_{2}\left(x,t\right)\right|\right)|w_{1}{\left(x,t\right)|}^{2}dx-2\sum_{k=1}^{4}\int_{\Omega }D_{k}\left(x\right){\left|\nabla w_{k}\left(x,t\right)\right|}^{2}dx\\ \; & -\int_{\Omega }\left(2v_{1}\left(x,t\right)w_{1}\left(x,t\right)w_{2}\left(x,t\right)-|v_{1}\left(x,t\right)||w_{1}{\left(x,t\right)|}^{2}-|v_{1}\left(x,t\right)||w_{2}{\left(x,t\right)|}^{2}\right)dx\\ \; & -\beta \int_{\Omega }\left(2v_{2}\left(x,t\right)w_{1}\left(x,t\right)w_{4}\left(x,t\right)-|v_{2}\left(x,t\right)||w_{1}{\left(x,t\right)|}^{2}-|v_{2}\left(x,t\right)||w_{4}{\left(x,t\right)|}^{2}\right)dx\\ \; & -\beta \int_{\Omega }\left(2v_{1}\left(x,t\right)w_{2}\left(x,t\right)w_{4}\left(x,t\right)-|v_{1}\left(x,t\right)||w_{2}{\left(x,t\right)|}^{2}-|v_{1}\left(x,t\right)||w_{4}{\left(x,t\right)|}^{2}\right)dx\\ \; & -2\int_{\Omega }{\left(\begin{array}{c} w_{1}\left(x,t\right)\\ w_{2}\left(x,t\right)\\ w_{3}\left(x,t\right)\\ w_{4}\left(x,t\right) \end{array}\right)}^{⊤}\left(\begin{array}{cccc} m_{11}+a & 0 & 0 & -\frac{1}{2}\\ 0 & m_{21}+b\; & 0 & 0\\ 0 & 0 & m_{31}+c & 0\\ -\frac{1}{2} & 0 & 0 & m_{31}+\alpha \end{array}\right)\left(\begin{array}{c} w_{1}\left(x,t\right)\\ w_{2}\left(x,t\right)\\ w_{3}\left(x,t\right)\\ w_{4}\left(x,t\right) \end{array}\right)dx\\ \; & -\frac{4\beta }{3}\int_{\Omega }\left(|w_{1}{\left(x,t\right)|}^{3}+|w_{2}{\left(x,t\right)|}^{3}+{\left|w_{4}\left(x,t\right)\right|}^{3}\right)dx\\ \; & -2\beta \int_{\Omega }w_{1}\left(x,t\right)w_{2}\left(x,t\right)w_{4}\left(x,t\right)dx-2\sum_{k=1}^{4}m_{k3}\int_{\Omega }{\left|w_{k}\left(x,t\right)\right|}^{2\gamma }dx\\ \; & -2{\left(\sum_{k=1}^{4}{∥w_{k}\left(\cdot ,t\right)∥}_{L^{2}\left(\Omega \right)}^{2}\right)}^{\mu -1}\sum_{k=1}^{4}m_{k2}{∥w_{k}\left(\cdot ,t\right)∥}_{L^{2}\left(\Omega \right)}^{2}\\ ⩽\; & -2{\left(\sum_{k=1}^{4}{∥w_{k}\left(\cdot ,t\right)∥}_{L^{2}\left(\Omega \right)}^{2}\right)}^{\mu -1}\sum_{k=1}^{4}m_{k2}∥w_{k}{\left(\cdot ,t\right)∥}_{L^{2}\left(\Omega \right)}^{2}-2\sum_{k=1}^{4}m_{k3}\int_{\Omega }{\left|w_{k}\left(x,t\right)\right|}^{2\gamma }dx\\ ⩽\; & -2\min_{1⩽k⩽4}m_{k2}{\left(V(t)\right)}^{\mu }-2\min_{1⩽k⩽4}m_{k3}\sum_{k=1}^{4}\int_{\Omega }{\left|w_{k}\left(x,t\right)\right|}^{2\gamma }dx\\ ⩽\; & -2\min_{1⩽k⩽4}m_{k2}{\left(V(t)\right)}^{\mu }-2{\left(\mathrm{meas}\Omega \right)}^{1-\gamma }\min_{1⩽k⩽4}m_{k3}\sum_{k=1}^{4}{\left(\int_{\Omega }{\left|w_{k}\left(x,t\right)\right|}^{2}dx\right)}^{\gamma }\\ ⩽\; & -2\min_{1⩽k⩽4}m_{k2}{\left(V(t)\right)}^{\mu }-2{\left(4\mathrm{meas}\Omega \right)}^{1-\gamma }\min_{1⩽k⩽4}m_{k3}{\left(V(t)\right)}^{\gamma },\;t\in R^{+}. \end{array}$$

By Lemma 3, there exists a positive time instant $T_{0}$ fulfilling (57) such that

$$\begin{array}{cccc} \; & V(t)\equiv 0,\; & \; & t\in [T_{0},+\infty ). \end{array}$$

This, together with (59), implies that the proof of Theorem 5 is complete. □

It is observed in Reference [18] that (hyper)chaotic financial systems cannot always be completely synchronized, but by Theorem 5, the control (48) could certainly synchronize the drive-response financial system (3)–(5) in a fixed time. It is worth giving several remarks concerning the possibility to improve the control (48).

- In the control law (48), the term
  $$-\frac{2\beta }{3}\left|{\tilde{v}}_{k}\left(x,t\right)-v_{k}\left(x,t\right)\right|\left({\tilde{v}}_{k}\left(x,t\right)-v_{k}\left(x,t\right)\right)$$
  in $W_{k}\left(x,t\right)$ can be ‘weakened’ to
  $$-\frac{(1+\vartheta )\beta }{3}\left|{\tilde{v}}_{k}\left(x,t\right)-v_{k}\left(x,t\right)\right|\left({\tilde{v}}_{k}\left(x,t\right)-v_{k}\left(x,t\right)\right),$$
  where ϑ is any positive constant, k=1,2,4. We insist on requiring that the constant ϑ is positive to guarantee that $w_{k}$ belongs to $L_{\mathrm{loc}}^{3}\left(\left[0,+\infty \right);L^{3}\left(\Omega \right)\right)$, k=1,2,4, where ${\left(w_{1},w_{2},w_{3},w_{4}\right)}^{⊤}$ is any trajectory quadruple of the controlled error system (51).
- In the control law (48), it depends not only on the structure of the drive-response financial system (3)–(5), but also on the information of the trajectory ${\left(v_{1},v_{2},v_{3},v_{4}\right)}^{⊤}$ of the drive financial system (3). More precisely, in the control law (48), the control $W_{1}\left(x,t\right)$ includes
  $$-(\frac{1}{2}|v_{1}\left(x,t\right)|+\frac{\beta +2}{2}|v_{2}\left(x,t\right)\left|\right)\left({\tilde{v}}_{1}\left(x,t\right)-v_{1}\left(x,t\right)\right),$$
  the control $W_{2}\left(x,t\right)$ includes
  $$-\frac{1+\beta }{2}\left|v_{1}\left(x,t\right)\right|\left({\tilde{v}}_{2}\left(x,t\right)-v_{2}\left(x,t\right)\right),$$
  and the control $W_{4}\left(x,t\right)$ includes
  $$-(\frac{\beta }{2}|v_{1}\left(x,t\right)|+\frac{\beta }{2}|v_{2}\left(x,t\right)\left|\right)\left({\tilde{v}}_{4}\left(x,t\right)-v_{4}\left(x,t\right)\right).$$
  From the point of view of system or control theory, it is flawed to incorporate trajectory information in the synchronization control. The flaw in the control law (48) arises due to the nonlinearity of the financial system (3). This flaw could be eliminated by restricting the trajectory quadruple ${\left(v_{1},v_{2},v_{3},v_{4}\right)}^{⊤}$ of the financial system (3) to be bounded in $L^{\infty }\left(\Omega ;R^{4}\right)$.

**Theorem** **6.** *Suppose that Assumptions 1 and 2 hold true. Assume that $m_{kh}\in \left(0,+\infty \right)$, k=1,2,3,4, h=2,3, and that $m_{k1}\in R^{+}$ (k=1,2,3,4) render the matrix*

$$\left(\begin{array}{cccc} m_{11}+a & 0 & 0 & -\frac{1}{2}\\ 0 & m_{21}+b\; & 0 & 0\\ 0 & 0 & m_{31}+c & 0\\ -\frac{1}{2} & 0 & 0 & m_{31}+\alpha \end{array}\right)$$

*to be semi-positive definite. For every pair (μ,γ), with 0<μ<1<γ, there exists a positive time instant $T_{0}$ satisfying (57), such that for every quadruple ${\left(v_{1}^{0},v_{2}^{0},v_{3}^{0},v_{4}^{0}\right)}^{⊤}$ of initial data in the Hilbert space $L^{2}\left(\Omega ;R^{4}\right)$ rendering the corresponding solution quadruple ${\left(v_{1},v_{2},v_{3},v_{4}\right)}^{⊤}$ to the initial-boundary value problem (3)–(7) satisfies*

$$\max_{1⩽k⩽4}\underset{t\in R^{+}}{ess sup}{∥v_{k}\left(\cdot ,t\right)∥}_{L^{\infty }\left(\Omega \right)}⩽B,$$

*where B is a given absolute positive constant, and every quadruple ${\left({\tilde{v}}_{1}^{0},{\tilde{v}}_{2}^{0},{\tilde{v}}_{3}^{0},{\tilde{v}}_{4}^{0}\right)}^{⊤}$ of initial data in the Hilbert space $L^{2}\left(\Omega ;R^{4}\right)$, we have*

$$\begin{array}{cccc} \; & \sum_{k=1}^{4}{∥{\tilde{v}}_{k}\left(\cdot ,t\right)-v_{k}\left(\cdot ,t\right)∥}_{L^{2}\left(\Omega \right)}^{2}=0, & \; & t\in [T_{0},+\infty ), \end{array}$$

*where the quadruple ${\left({\tilde{v}}_{1},{\tilde{v}}_{2},{\tilde{v}}_{3},{\tilde{v}}_{4}\right)}^{⊤}$ is the unique trajectory of the system (5)–(8) upon which the following control is implemented:*

$$\begin{array}{cc} W_{1}\left(x,t\right)=\; & -\left(m_{11}+\frac{B(\beta +3)}{2}\right)\left({\tilde{v}}_{1}\left(x,t\right)-v_{1}\left(x,t\right)\right)\\ \; & -m_{12}{\left(\sum_{k=1}^{4}{∥{\tilde{v}}_{k}\left(\cdot ,t\right)-v_{k}\left(\cdot ,t\right)∥}_{L^{2}\left(\Omega \right)}^{2}\right)}^{\mu -1}\left({\tilde{v}}_{1}\left(x,t\right)-v_{1}\left(x,t\right)\right)\\ \; & -m_{13}{\left|{\tilde{v}}_{1}\left(x,t\right)-v_{1}\left(x,t\right)\right|}^{2\gamma -2}\left({\tilde{v}}_{1}\left(x,t\right)-v_{1}\left(x,t\right)\right)\\ \; & -\frac{2\beta }{3}\left|{\tilde{v}}_{1}\left(x,t\right)-v_{1}\left(x,t\right)\right|\left({\tilde{v}}_{1}\left(x,t\right)-v_{1}\left(x,t\right)\right),\\ W_{2}\left(x,t\right)=\; & -\left(m_{21}+\frac{B(1+\beta )}{2}\right)\left({\tilde{v}}_{2}\left(x,t\right)-v_{2}\left(x,t\right)\right)\\ \; & -m_{22}{\left(\sum_{k=1}^{4}{∥{\tilde{v}}_{k}\left(\cdot ,t\right)-v_{k}\left(\cdot ,t\right)∥}_{L^{2}\left(\Omega \right)}^{2}\right)}^{\mu -1}\left({\tilde{v}}_{2}\left(x,t\right)-v_{2}\left(x,t\right)\right)\\ \; & -m_{23}{\left|{\tilde{v}}_{2}\left(x,t\right)-v_{2}\left(x,t\right)\right|}^{2\gamma -2}\left({\tilde{v}}_{2}\left(x,t\right)-v_{2}\left(x,t\right)\right)\\ \; & -\frac{2\beta }{3}\left|{\tilde{v}}_{2}\left(x,t\right)-v_{2}\left(x,t\right)\right|\left({\tilde{v}}_{2}\left(x,t\right)-v_{2}\left(x,t\right)\right),\\ W_{3}\left(x,t\right)=\; & -m_{31}\left({\tilde{v}}_{3}\left(x,t\right)-v_{3}\left(x,t\right)\right)-m_{33}{\left|{\tilde{v}}_{3}\left(x,t\right)-v_{3}\left(x,t\right)\right|}^{2\gamma -2}\left({\tilde{v}}_{3}\left(x,t\right)-v_{3}\left(x,t\right)\right)\\ \; & -m_{32}{\left(\sum_{k=1}^{4}{∥{\tilde{v}}_{k}\left(\cdot ,t\right)-v_{k}\left(\cdot ,t\right)∥}_{L^{2}\left(\Omega \right)}^{2}\right)}^{\mu -1}\left({\tilde{v}}_{3}\left(x,t\right)-v_{3}\left(x,t\right)\right),\\ W_{4}\left(x,t\right)=\; & -\left(m_{41}+B\beta \right)\left({\tilde{v}}_{4}\left(x,t\right)-v_{4}\left(x,t\right)\right)\\ \; & -m_{42}{\left(\sum_{k=1}^{4}{∥{\tilde{v}}_{k}\left(\cdot ,t\right)-v_{k}\left(\cdot ,t\right)∥}_{L^{2}\left(\Omega \right)}^{2}\right)}^{\mu -1}\left({\tilde{v}}_{4}\left(x,t\right)-v_{4}\left(x,t\right)\right)\\ \; & -m_{43}{\left|{\tilde{v}}_{4}\left(x,t\right)-v_{4}\left(x,t\right)\right|}^{2\gamma -2}\left({\tilde{v}}_{4}\left(x,t\right)-v_{4}\left(x,t\right)\right)\\ \; & -\frac{2\beta }{3}\left|{\tilde{v}}_{4}\left(x,t\right)-v_{4}\left(x,t\right)\right|\left({\tilde{v}}_{4}\left(x,t\right)-v_{4}\left(x,t\right)\right). \end{array}$$

**Proof.**  The proof of Theorem 6 actually resembles that of Theorem 5. Therefore, we choose to omit here the details of the proof. □

## 4. Numerical Simulations

In this section, we shall perform some numerical simulations to illustrate that our suggested control law (48) is effective in synchronizing the drive financial system (3) and the response system (5) in a fixed time. The basic assumption in our numerical research is that Ω=(0,8), a=0.9, b=0.2, c=1.5, α=0.17, β=0.2, and $D_{1}\left(x\right)=D_{2}\left(x\right)=D_{3}\left(x\right)=D_{4}\left(x\right)\equiv 0.001$ always hold in the drive system (3) and the response system (5).

**Figure 1:**
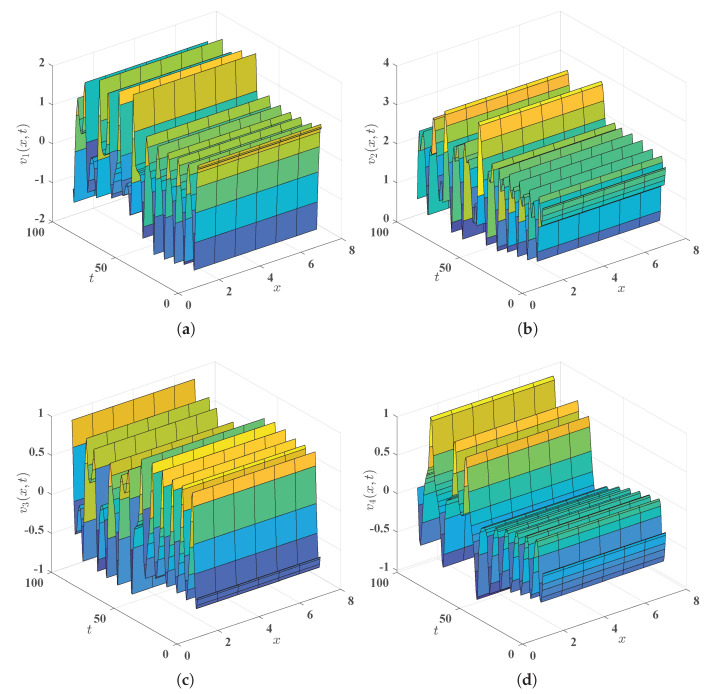
Numerical and graphical illustration of the occurrence of chaos phenomenon in the financial system (3) (an infinite-dimensional dynamical system) distributed in a line segment: The quadruple ${\left(v_{1}\left(x,t\right),v_{2}\left(x,t\right),v_{3}\left(x,t\right),v_{4}\left(x,t\right)\right)}^{⊤}$ denotes the solution to the initial-boundary value problem (3)–(7), with the interval Ω=(0,8), the parameters a=0.9, b=0.2, c=1.5, α=0.17, and β=0.2, the diffusion coefficients $D_{1}\left(x\right)=D_{2}\left(x\right)=D_{3}\left(x\right)=D_{4}\left(x\right)\equiv 0.001$, and the initial data $v_{1}^{0}\left(x\right)\equiv 1$, $v_{2}^{0}\left(x\right)\equiv 2$, $v_{3}^{0}\left(x\right)\equiv -0.5$, and $v_{4}^{0}\left(x\right)\equiv -0.2$, x∈Ω=(0,8); (**a**–**d**) display the graphs (surfaces) of the functions (having two indeterminates *x* and *t*) $v_{1}=v_{1}\left(x,t\right)$, $v_{2}=v_{2}\left(x,t\right)$, and $v_{3}=v_{3}\left(x,t\right)$, respectively, x∈Ω=(0,8), t∈[0,100]; the unit against which the time *t* is measured could be set arbitrarily to be second, hour, day, week, month, or some other suitable time period; the unit against which the space variable *x* is measured could be set arbitrarily to be meter, kilometre, or some other reasonable reference standard for measurement of length; the units of the time *t* and space variable *x* are actually chosen, in an arbitrary way, and fixed at the very beginning of the construction of the financial model (3); as with the space variable *x* and the time *t*, the units against which the economic quantities $v_{k}$ are measured (see Section 1 for the brief introduction of $v_{k}$), k=1,2,3,4, can also be set in several different ways; during our choosing the aforementioned units, we should abide by two basic rules (i) the choice of unit of each economic quantity $v_{k}$ should not contradict the choices of the units of the other three economic quantities, and (ii) the choices of units of the space variable *x*, the time *t*, and the economic quantities $v_{k}$ (k=1,2,3,4) should facilitate later calculations and applications of the obtained theoretical results, we could choose, say, the aforementioned units as in References [1,5].

**Figure 2:**
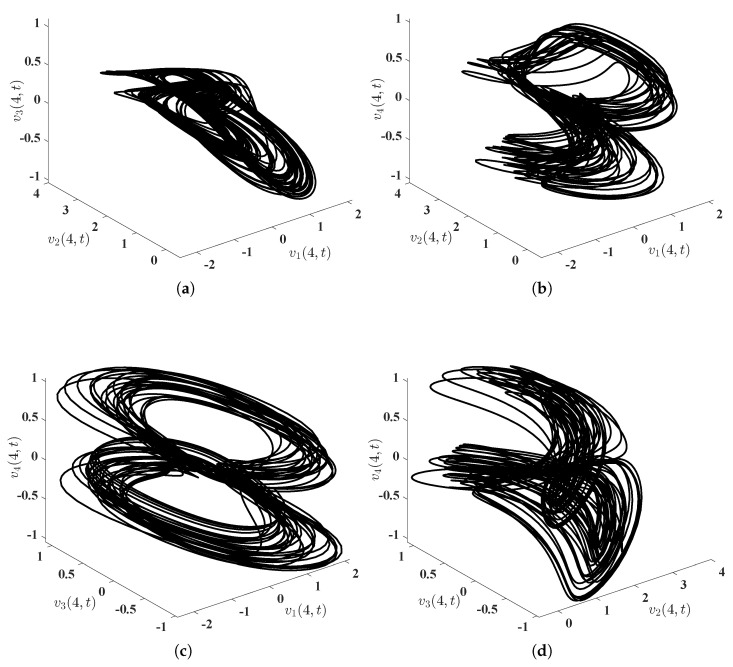
Numerical and graphical illustration of the occurrence of chaos phenomenon in the financial system (3) (an infinite-dimensional dynamical system) distributed in the line segment Ω=(0,8). The quadruple ${\left(v_{1}\left(x,t\right),v_{2}\left(x,t\right),v_{3}\left(x,t\right),v_{4}\left(x,t\right)\right)}^{⊤}$ denotes the solution, in the time interval [0,100], to the initial-boundary value problem (3)–(7), with the parameters a=0.9, b=0.2, c=1.5, α=0.17, and β=0.2, the diffusion coefficients $D_{1}\left(x\right)=D_{2}\left(x\right)=D_{3}\left(x\right)=D_{4}\left(x\right)\equiv 0.001$, and the initial data $v_{1}^{0}\left(x\right)\equiv 1$, $v_{2}^{0}\left(x\right)\equiv 2$, $v_{3}^{0}\left(x\right)\equiv -0.5$ and $v_{4}^{0}\left(x\right)\equiv -0.2$, x∈Ω=(0,8); the quadruple ${\left(v_{1}\left(4,t\right),v_{2}\left(4,t\right),v_{3}\left(4,t\right),v_{4}\left(4,t\right)\right)}^{⊤}$ denotes the restriction to the line segment {x;x=4}×[0,100] of the quadruple ${\left(v_{1}\left(x,t\right),v_{2}\left(x,t\right),v_{3}\left(x,t\right),v_{4}\left(x,t\right)\right)}^{⊤}$; (**a**–**d**) display the projections (three-dimensional curves) onto the three-dimensional Euclidean spaces $v_{1}-v_{2}-v_{3}$, $v_{1}-v_{2}-v_{4}$, $v_{1}-v_{3}-v_{4}$, and $v_{2}-v_{3}-v_{4}$, respectively, of the parametrized curve ${\left(v_{1}\left(4,t\right),v_{2}\left(4,t\right),v_{3}\left(4,t\right),v_{4}\left(4,t\right)\right)}^{⊤}$ in the four-dimensional Euclidean space $R^{4}$ with the time parameter *t* running over the interval [0,100]; see Figure 1 for the detailed explanation of the choices of the units against which the space variable *x*, the time *t*, and the economic quantities $v_{k}$ (k=1,2,3,4) are measured.

**Figure 3:**
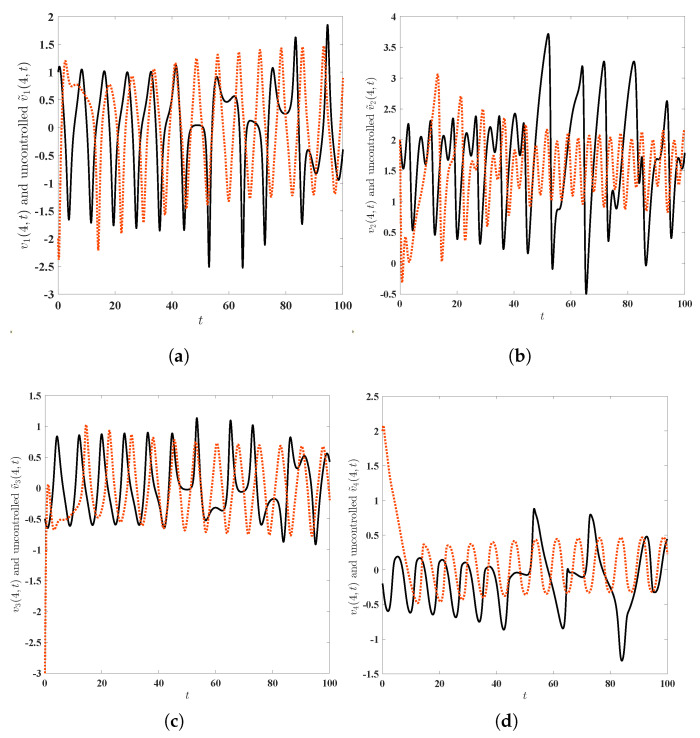
Numerical and graphical illustration of the asynchrony of the drive financial system (3) and its response system (5), without any control input, distributed in the line segment Ω=(0,8). The quadruples ${\left(v_{1}\left(x,t\right),v_{2}\left(x,t\right),v_{3}\left(x,t\right),v_{4}\left(x,t\right)\right)}^{⊤}$ and ${\left({\tilde{v}}_{1}\left(x,t\right),{\tilde{v}}_{2}\left(x,t\right),{\tilde{v}}_{3}\left(x,t\right),{\tilde{v}}_{4}\left(x,t\right)\right)}^{⊤}$ denote the solutions, in the time interval [0,100], to the initial-boundary value problems (3)–(7) and (5)–(8), respectively, with the parameters a=0.9, b=0.2, c=1.5, α=0.17, and β=0.2, the diffusion coefficients $D_{1}\left(x\right)=D_{2}\left(x\right)=D_{3}\left(x\right)=D_{4}\left(x\right)\equiv 0.001$, the initial data $v_{1}^{0}\left(x\right)\equiv 1$, $v_{2}^{0}\left(x\right)\equiv 2$, $v_{3}^{0}\left(x\right)\equiv -0.5$ and $v_{4}^{0}\left(x\right)\equiv -0.2$, the initial data ${\tilde{v}}_{1}^{0}\left(x\right)=\cos\left(\frac{\pi }{4}x\right)-1$, ${\tilde{v}}_{2}^{0}\left(x\right)=\cos\left(\frac{\pi }{2}x\right)+1$, ${\tilde{v}}_{3}^{0}\left(x\right)=-\cos\left(\pi x\right)-2$, and ${\tilde{v}}_{4}^{0}\left(x\right)=-\cos\left(\frac{\pi }{4}x\right)+1$, the control $W_{1}\left(x,t\right)=W_{2}\left(x,t\right)=W_{3}\left(x,t\right)=W_{4}\left(x,t\right)\equiv 0$, x∈Ω=(0,8), t∈[0,100]; the quadruples ${\left(v_{1}\left(4,t\right),v_{2}\left(4,t\right),v_{3}\left(4,t\right),v_{4}\left(4,t\right)\right)}^{⊤}$ and ${\left({\tilde{v}}_{1}\left(4,t\right),{\tilde{v}}_{2}\left(4,t\right),{\tilde{v}}_{3}\left(4,t\right),{\tilde{v}}_{4}\left(4,t\right)\right)}^{⊤}$ denote the restrictions to the line segment {x;x=4}×[0,100] of the quadruples ${\left(v_{1}\left(x,t\right),v_{2}\left(x,t\right),v_{3}\left(x,t\right),v_{4}\left(x,t\right)\right)}^{⊤}$ and ${\left({\tilde{v}}_{1}\left(x,t\right),{\tilde{v}}_{2}\left(x,t\right),{\tilde{v}}_{3}\left(x,t\right),{\tilde{v}}_{4}\left(x,t\right)\right)}^{⊤}$, respectively; (**a**–**d**) display the graphs (curves) of $v_{1}=v_{1}\left(4,t\right)$ (the solid curve) vs. ${\tilde{v}}_{1}={\tilde{v}}_{1}\left(4,t\right)$ (the dotted curve), $v_{2}=v_{2}\left(4,t\right)$ (the solid curve) vs. ${\tilde{v}}_{2}={\tilde{v}}_{2}\left(4,t\right)$ (the dotted curve), $v_{3}=v_{3}\left(4,t\right)$ (the solid curve) vs. ${\tilde{v}}_{3}={\tilde{v}}_{3}\left(4,t\right)$ (the dotted curve), and $v_{4}=v_{4}\left(4,t\right)$ (the solid curve) vs. ${\tilde{v}}_{4}={\tilde{v}}_{4}\left(4,t\right)$ (the dotted curve), respectively, with the time parameter *t* running over the interval [0,100]; see Figure 1 for the detailed explanation of the choices of the units against which the space variable *x*, the time *t*, and the economic quantities $v_{k}$ (k=1,2,3,4) are measured.

First of all, we solve numerically, via MATLAB, the initial-boundary value problem (3)–(7) with $v_{1}^{0}\left(x\right)\equiv 1$, $v_{2}^{0}\left(x\right)\equiv 2$, $v_{3}^{0}\left(x\right)\equiv -0.5$, $v_{4}^{0}\left(x\right)\equiv -0.2$, x∈(0,8), to arrive at the solution, denoted by ${\left(v_{1}\left(x,t\right),v_{2}\left(x,t\right),v_{3}\left(x,t\right),v_{4}\left(x,t\right)\right)}^{⊤}$. The surfaces (see Figure 1) of this solution and projections of this solution at the midpoint x=4 of the interval Ω=(0,8) (see Figure 2) ‘demonstrate’ visually and intuitively that the drive financial system (3) is chaotic. Similarly, we solve numerically, via MATLAB, the initial-boundary value problem (5)–(8)–(48) with $W_{1}=W_{2}=W_{3}=W_{4}\equiv 0$ (in other words, there is no control implemented upon the response system in this situation), ${\tilde{v}}_{1}^{0}\left(x\right)=\cos\left(\frac{\pi }{4}x\right)-1$, ${\tilde{v}}_{2}^{0}\left(x\right)=\cos\left(\frac{\pi }{2}x\right)+1$, ${\tilde{v}}_{3}^{0}\left(x\right)=-\cos\left(\pi x\right)-2$, ${\tilde{v}}_{4}^{0}\left(x\right)=-\cos\left(\frac{\pi }{4}x\right)+1$, to arrive at ${\left({\tilde{v}}_{1}\left(x,t\right),{\tilde{v}}_{2}\left(x,t\right),{\tilde{v}}_{3}\left(x,t\right),{\tilde{v}}_{4}\left(x,t\right)\right)}^{⊤}$. Thanks to the chaos phenomenon in the system (3) (see Figure 1 and Figure 2), we are not inclined to imagine that ${\left({\tilde{v}}_{1}\left(x,t\right),{\tilde{v}}_{2}\left(x,t\right),{\tilde{v}}_{3}\left(x,t\right),{\tilde{v}}_{4}\left(x,t\right)\right)}^{⊤}$ approaches ${\left(v_{1}\left(x,t\right),v_{2}\left(x,t\right),v_{3}\left(x,t\right),v_{4}\left(x,t\right)\right)}^{⊤}$ as time *t* escapes to infinity. Actually, the quadruple ${\left({\tilde{v}}_{1}\left(x,t\right),{\tilde{v}}_{2}\left(x,t\right),{\tilde{v}}_{3}\left(x,t\right),{\tilde{v}}_{4}\left(x,t\right)\right)}^{⊤}$ does not approach the quadruple ${\left(v_{1}\left(x,t\right),v_{2}\left(x,t\right),v_{3}\left(x,t\right),v_{4}\left(x,t\right)\right)}^{⊤}$ as t→+∞ can also be numerically ‘proved’; see Figure 3. To summarize here, the numerical simulations mentioned in this paragraph reveal that the drive financial system (3) and the response system (5) cannot achieve synchronization in a fixed time unless extra control is implemented.

**Figure 4:**
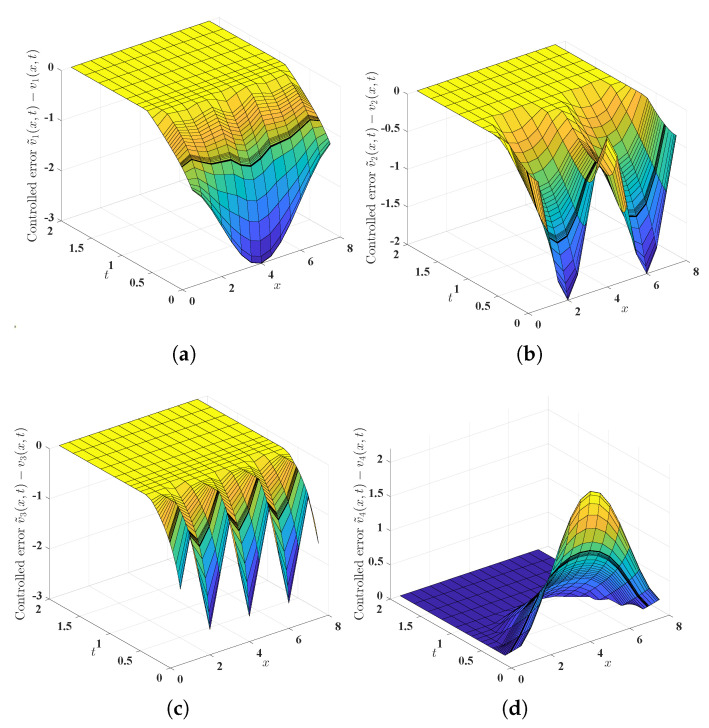
Numerical and graphical illustration of the idea that the response system (5) could be synchronized, in fixed time, to the drive financial system (3), distributed in the line segment Ω=(0,8), by the control law (48): The quadruples ${\left(v_{1}\left(x,t\right),v_{2}\left(x,t\right),v_{3}\left(x,t\right),v_{4}\left(x,t\right)\right)}^{⊤}$ and ${\left({\tilde{v}}_{1}\left(x,t\right),{\tilde{v}}_{2}\left(x,t\right),{\tilde{v}}_{3}\left(x,t\right),{\tilde{v}}_{4}\left(x,t\right)\right)}^{⊤}$ denote the solutions, in the time interval [0,2], to the initial-boundary value problems (3)–(7) and (5)–(8) supplemented by the nonlinear term (48), respectively, with the parameters a=0.9, b=0.2, c=1.5, α=0.17, β=0.2, $\mu =\frac{1}{2}$, γ=2, $m_{kh}=1$ (k=1,2,3,4, h=2,3), $m_{11}=m_{21}=m_{31}=0$, and $m_{41}=0.1078$; the diffusion coefficients $D_{1}\left(x\right)=D_{2}\left(x\right)=D_{3}\left(x\right)=D_{4}\left(x\right)\equiv 0.001$, the initial data $v_{1}^{0}\left(x\right)\equiv 1$, $v_{2}^{0}\left(x\right)\equiv 2$, $v_{3}^{0}\left(x\right)\equiv -0.5$, and $v_{4}^{0}\left(x\right)\equiv -0.2$, the initial data ${\tilde{v}}_{1}^{0}\left(x\right)=\cos\left(\frac{\pi }{4}x\right)-1$, ${\tilde{v}}_{2}^{0}\left(x\right)=\cos\left(\frac{\pi }{2}x\right)+1$, ${\tilde{v}}_{3}^{0}\left(x\right)=-\cos\left(\pi x\right)-2$, and ${\tilde{v}}_{4}^{0}\left(x\right)=-\cos\left(\frac{\pi }{4}x\right)+1$, x∈Ω=(0,8); (**a**–**d**) display the graphs (surfaces) of the functions (having two independent variables, that is, *x* and *t*) ${\tilde{v}}_{1}\left(x,t\right)-v_{1}\left(x,t\right)$, ${\tilde{v}}_{2}\left(x,t\right)-v_{2}\left(x,t\right)$, ${\tilde{v}}_{3}\left(x,t\right)-v_{3}\left(x,t\right)$, and ${\tilde{v}}_{4}\left(x,t\right)-v_{4}\left(x,t\right)$, respectively, x∈Ω=(0,8), t∈[0,2]; see Figure 1 for the detailed explanation of the choices of the units against which the space variable *x*, the time *t*, and the economic quantities $v_{k}$ (k=1,2,3,4) are measured.

**Figure 5:**
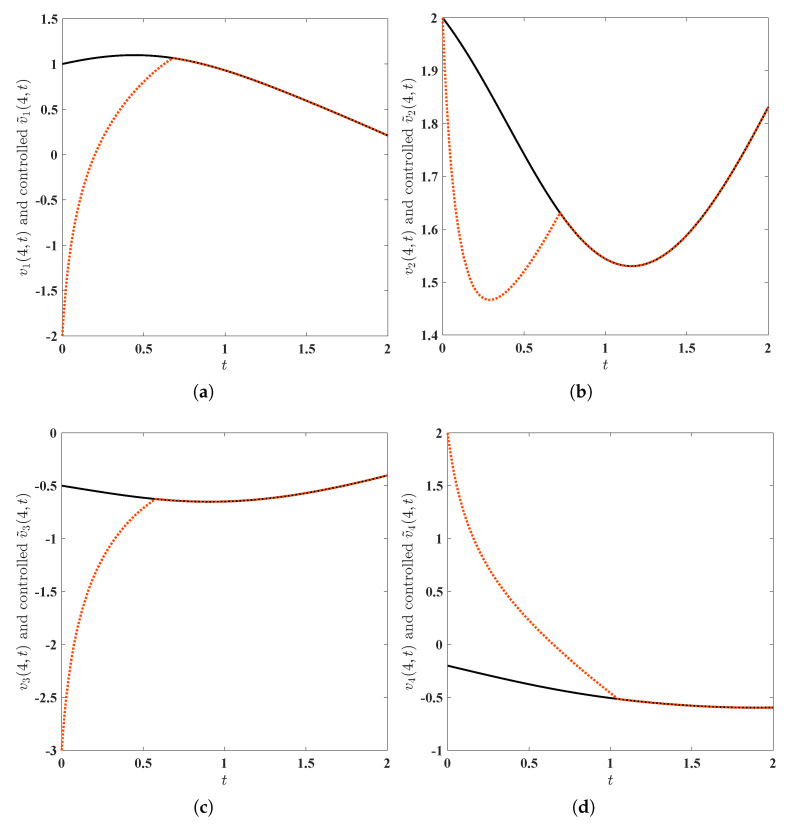
Numerical and graphical illustration of the idea that that the response system (5) could be synchronized, in fixed time, to the drive financial system (3), distributed in the line segment Ω=(0,8), by the control law (48): The quadruples ${\left(v_{1}\left(x,t\right),v_{2}\left(x,t\right),v_{3}\left(x,t\right),v_{4}\left(x,t\right)\right)}^{⊤}$ and ${\left({\tilde{v}}_{1}\left(x,t\right),{\tilde{v}}_{2}\left(x,t\right),{\tilde{v}}_{3}\left(x,t\right),{\tilde{v}}_{4}\left(x,t\right)\right)}^{⊤}$ denote the solutions, in the time interval [0,2], to the initial-boundary value problems (3)–(7) and (5)–(8)–(48), respectively, with the parameters a=0.9, b=0.2, c=1.5, α=0.17, β=0.2, $\mu =\frac{1}{2}$, γ=2, $m_{kh}=1$ (k=1,2,3,4, h=2,3), $m_{11}=m_{21}=m_{31}=0$, and $m_{41}=0.1078$, the diffusion coefficients $D_{1}\left(x\right)=D_{2}\left(x\right)=D_{3}\left(x\right)=D_{4}\left(x\right)\equiv 0.001$, the initial data $v_{1}^{0}\left(x\right)\equiv 1$, $v_{2}^{0}\left(x\right)\equiv 2$, $v_{3}^{0}\left(x\right)\equiv -0.5$, and $v_{4}^{0}\left(x\right)\equiv -0.2$, the initial data ${\tilde{v}}_{1}^{0}\left(x\right)=\cos\left(\frac{\pi }{4}x\right)-1$, ${\tilde{v}}_{2}^{0}\left(x\right)=\cos\left(\frac{\pi }{2}x\right)+1$, ${\tilde{v}}_{3}^{0}\left(x\right)=-\cos\left(\pi x\right)-2$, and ${\tilde{v}}_{4}^{0}\left(x\right)=-\cos\left(\frac{\pi }{4}x\right)+1$, x∈Ω=(0,8); the quadruples ${\left(v_{1}\left(4,t\right),v_{2}\left(4,t\right),v_{3}\left(4,t\right),v_{4}\left(4,t\right)\right)}^{⊤}$ and ${\left({\tilde{v}}_{1}\left(4,t\right),{\tilde{v}}_{2}\left(4,t\right),{\tilde{v}}_{3}\left(4,t\right),{\tilde{v}}_{4}\left(4,t\right)\right)}^{⊤}$ denote the restrictions to the line segment {x;x=4}×[0,2] of the quadruples ${\left(v_{1}\left(x,t\right),v_{2}\left(x,t\right),v_{3}\left(x,t\right),v_{4}\left(x,t\right)\right)}^{⊤}$ and ${\left({\tilde{v}}_{1}\left(x,t\right),{\tilde{v}}_{2}\left(x,t\right),{\tilde{v}}_{3}\left(x,t\right),{\tilde{v}}_{4}\left(x,t\right)\right)}^{⊤}$, respectively; (**a**–**d**) display the graphs (curves) of $v_{1}=v_{1}\left(4,t\right)$ (the solid curve) vs. ${\tilde{v}}_{1}={\tilde{v}}_{1}\left(4,t\right)$ (the dotted curve), $v_{2}=v_{2}\left(4,t\right)$ (the solid curve) vs. ${\tilde{v}}_{2}={\tilde{v}}_{2}\left(4,t\right)$ (the dotted curve), $v_{3}=v_{3}\left(4,t\right)$ (the solid curve) vs. ${\tilde{v}}_{3}={\tilde{v}}_{3}\left(4,t\right)$ (the dotted curve), and $v_{4}=v_{4}\left(4,t\right)$ (the solid curve) vs. ${\tilde{v}}_{4}={\tilde{v}}_{4}\left(4,t\right)$ (the dotted curve), respectively, with the time parameter *t* running over the interval [0,2]; see Figure 1 for the detailed explanation of the choices of the units against which the space variable *x*, the time *t*, and the economic quantities $v_{k}$ (k=1,2,3,4) are measured.

Illuminated by Theorem 5, we design the control (48) for the drive financial system (3) and the response system (5). As revealed in Theorem 5, the parameter μ can take an arbitrary value in (0,1) and γ can take an arbitrary value in (1,+∞). For the sake of simplicity, we put $\mu =\frac{1}{2}$ and γ=2. Additionally, as indicated in the synchronization criterion provided in Theorem 5, to obtain the desired fixed-time synchronizability, $m_{kh}$ (k=1,2,3,4, h=1,2,3) are required to satisfy $m_{kh}\in \left(0,+\infty \right)$ (k=1,2,3,4, h=2,3), and $m_{kh}\in R^{+}$ (k=1,2,3,4, h=1) render the matrix

$$\left(\begin{array}{cccc} m_{11}+0.9 & 0 & 0 & -\frac{1}{2}\\ 0 & m_{21}+0.2\; & 0 & 0\\ 0 & 0 & m_{31}+1.5 & 0\\ -\frac{1}{2} & 0 & 0 & m_{31}+0.17 \end{array}\right)$$

to be semi-positive definite (it is not difficult to find that the semi-positive definiteness is equivalent to $m_{21}\in R^{+}$, $m_{31}\in R^{+}$ and $\left(m_{11}+0.9\right)\left(m_{41}+0.17\right)⩾0.25$). For the convenience of later computations, we put $m_{kh}=1$ (k=1,2,3,4, h=2,3), $m_{11}=m_{21}=m_{31}=0$, $m_{41}=0.1078$. Theoretically, these choices could guarantee that the drive financial system (3) and the response system (5) achieve fixed-time synchronization; see Theorem 5. After some computations, we find that [17,+∞) is contained in the totality of the settling times. As plausibly shown by Figure 4 and Figure 5, our theoretical results of this paper (see Theorems 5 and 6) are indeed effective.

## 5. Conclusions

In this paper, are focused on studying a financial system, comprising the labor force, the stock, the money, and the production sub-blocks distributed in a certain line segment or planar region, whose dynamics can be governed by the system (3) of semi-linear parabolic partial differential equations supplemented by the homogeneous Neumann boundary condition. We obtained the new financial system (3) by adding diffusion terms to the well-studied financial system (2), which was shown by Yu, Cai, and Li [5] to be hyperchaotic. In principle, chaos in dynamical systems causes the generated time series to display high entropy values. This phenomenon for the financial system (2), the basis for the new system (3), was illustrated graphically in Reference [18]. We provided economic/financial scenarios in which the diffusion terms should be added to the classical financial system (2), yielding the system (3). Our explanation complements the motivations provided in References [9,14] for introducing diffusion terms to the system (2).

We provided a precise definition of the trajectories of the financial system (3) and its corresponding (controlled or uncontrolled) response system (5) in the infinite dimensional state space $L^{2}\left(\Omega ;R^{4}\right)$. Based on the definition of trajectories, we proved that there exists a unique trajectory, existing globally in time, of the system (3) or the system (5), a continuous curve in the Hilbert space $L^{2}\left(\Omega ;R^{4}\right)$, with its initial state given arbitrarily in $L^{2}\left(\Omega ;R^{4}\right)$, and that trajectories the system (3) depend continuously on their initial states.

We proposed a synchronization control, namely (48), for the response system (5), and provided two criteria ensuring that the drive system (3) and the response system (5) with the proposed control (48) implemented achieve fixed-time synchronization. We also performed several numerical simulations to prove that, in a visional manner, our synchronization theoretical results in this paper are indeed effective.

To provide a precise definition of trajectories the systems (3) and (5), we ‘borrowed’ the notion of weak solutions of evolution partial differential equations. To prove the global (in time) existence of trajectories of the system (3) or the system (5), we used the celebrated Galerkin’s method and two a priori estimates on two modified energy functionals (can also be viewed as Lyapunov functionals) established in this paper. To prove that every element in $L^{2}\left(\Omega ;R^{4}\right)$ admits a unique trajectory of the system (3) or the system (5) having this element as its initial state, and that trajectories of the system (3) depend continuously on their initial states, we used semigroup theory of bounded linear operators in functional analysis. To provide the claimed fixed-time synchronizability criteria, we proposed a new novel Lyapunov functional (can be viewed as a certain modified energy functional).

## Appendix A. Proof of the Continuous Dependence and Uniqueness Parts of Theorem 1

In this section, we shall complete the proof of Theorem 1 by proving that for every initial datum, the initial-boundary value problem (3)–(7) admits a unique solution corresponding to this datum, and by proving that the data-to-solution is continuous in a certain sense. Let us recall first Euler’s Gamma function

Γ(z)=∫0+∞tz−1e−tdtforz∈CwithRez>0,

and the Mittag-Leffler function

$$\begin{array}{cccc} \; & E_{\upsilon }\left(z\right)=\sum_{k=0}^{\infty }\frac{z^{k}}{\Gamma (k\upsilon +1)}\; & \; & \;\mathrm{for}\;z\in C, \end{array}$$

where the parameter υ is required to be positive in this paper. It is not difficult to find that $E_{\upsilon }\left(z\right)$ is strictly increasing in $R^{+}$. To begin our proof, we prepare the following necessary lemma.

**Lemma** **A1** (see [43]). *Let υ,T∈(0,+∞). Suppose that the functions a(t) and b(t) are nondecreasing and take values in $R^{+}$. Let e(t) be a continuous function mapping [0,T] into $R^{+}$. If*

$$\begin{array}{cccc} \; & e\left(t\right)⩽a\left(t\right)+b\left(t\right)\int_{0}^{t}{\left(t-s\right)}^{\upsilon -1}e\left(s\right)ds\; & \; & \;\mathrm{for}\;t\in [0,T], \end{array}$$

*then*

$$\begin{array}{cccc} \; & e\left(t\right)⩽a\left(t\right)E_{\upsilon }\left(\Gamma \left(\upsilon \right)b\left(t\right)t^{\upsilon }\right)\; & \; & \;\mathrm{for}\;t\in [0,T]. \end{array}$$

**Proof.** We assume that ${\left({\overset{´}{v}}_{1},{\overset{´}{v}}_{2},{\overset{´}{v}}_{3},{\overset{´}{v}}_{4}\right)}^{⊤}$ and ${\left({\overset{`}{v}}_{1},{\overset{`}{v}}_{2},{\overset{`}{v}}_{3},{\overset{`}{v}}_{4}\right)}^{⊤}$, belonging to

$$C\left(\left[0,T\right];L^{2}\left(\Omega ;R^{4}\right)\right)\cap L^{2}\left(0,T;H^{1}\left(\Omega ;R^{4}\right)\right),$$

are solutions, in the same time interval [0,T], to the initial-boundary value problem (3)–(7), corresponding to the initial data ${\left({\overset{´}{v}}_{1}^{0},{\overset{´}{v}}_{2}^{0},{\overset{´}{v}}_{3}^{0},{\overset{´}{v}}_{4}^{0}\right)}^{⊤}$ and ${\left({\overset{`}{v}}_{1}^{0},{\overset{`}{v}}_{2}^{0},{\overset{`}{v}}_{3}^{0},{\overset{`}{v}}_{4}^{0}\right)}^{⊤}$, respectively. By applying Duhamel’s principle, we have

(A1) $${\overset{´}{v}}_{1}\left(\cdot ,t\right)=e^{tA_{1}}{\overset{´}{v}}_{1}^{0}+\int_{0}^{t}e^{\left(t-s\right)A_{1}}\left(\left({\overset{´}{v}}_{2}\left(\cdot ,s\right)-a\right){\overset{´}{v}}_{1}\left(\cdot ,s\right)+{\overset{´}{v}}_{3}\left(\cdot ,s\right)+{\overset{´}{v}}_{4}\left(\cdot ,s\right)\right)ds,$$

(A2) $${\overset{´}{v}}_{2}\left(\cdot ,t\right)=e^{tA_{2}}{\overset{´}{v}}_{2}^{0}+\int_{0}^{t}e^{\left(t-s\right)A_{2}}\left(1-b{\overset{´}{v}}_{2}\left(\cdot ,s\right)-{\left({\overset{´}{v}}_{1}\left(\cdot ,s\right)\right)}^{2}\right)ds,$$

(A3) $${\overset{´}{v}}_{3}\left(\cdot ,t\right)=e^{tA_{3}}{\overset{´}{v}}_{3}^{0}-\int_{0}^{t}e^{\left(t-s\right)A_{3}}\left(c{\overset{´}{v}}_{3}\left(\cdot ,s\right)+{\overset{´}{v}}_{1}\left(\cdot ,s\right)\right)ds,$$

(A4) $${\overset{´}{v}}_{4}\left(\cdot ,t\right)=e^{tA_{4}}{\overset{´}{v}}_{4}^{0}-\int_{0}^{t}e^{\left(t-s\right)A_{4}}\left(\alpha {\overset{´}{v}}_{4}\left(\cdot ,s\right)+\beta {\overset{´}{v}}_{1}\left(\cdot ,s\right){\overset{´}{v}}_{2}\left(\cdot ,s\right)\right)ds,$$

(A5) $${\overset{`}{v}}_{1}\left(\cdot ,t\right)=e^{tA_{1}}{\overset{`}{v}}_{1}^{0}+\int_{0}^{t}e^{\left(t-s\right)A_{1}}\left(\left({\overset{`}{v}}_{2}\left(\cdot ,s\right)-a\right){\overset{`}{v}}_{1}\left(\cdot ,s\right)+{\overset{`}{v}}_{3}\left(\cdot ,s\right)+{\overset{`}{v}}_{4}\left(\cdot ,s\right)\right)ds,$$

(A6) $${\overset{`}{v}}_{2}\left(\cdot ,t\right)=e^{tA_{2}}{\overset{`}{v}}_{2}^{0}+\int_{0}^{t}e^{\left(t-s\right)A_{2}}\left(1-b{\overset{`}{v}}_{2}\left(\cdot ,s\right)-{\left({\overset{`}{v}}_{1}\left(\cdot ,s\right)\right)}^{2}\right)ds,$$

(A7) $${\overset{`}{v}}_{3}\left(\cdot ,t\right)=e^{tA_{3}}{\overset{`}{v}}_{3}^{0}-\int_{0}^{t}e^{\left(t-s\right)A_{3}}\left(c{\overset{`}{v}}_{3}\left(\cdot ,s\right)+{\overset{`}{v}}_{1}\left(\cdot ,s\right)\right)ds,$$

and

(A8) $${\overset{`}{v}}_{4}\left(\cdot ,t\right)=e^{tA_{4}}{\overset{`}{v}}_{4}^{0}-\int_{0}^{t}e^{\left(t-s\right)A_{4}}\left(\alpha {\overset{`}{v}}_{4}\left(\cdot ,s\right)+\beta {\overset{`}{v}}_{1}\left(\cdot ,s\right){\overset{`}{v}}_{2}\left(\cdot ,s\right)\right)ds.$$

After some routine calculations, we deduce from (A1) and (A5) immediately

(A9) $$\begin{array}{cc} \; & {\overset{´}{v}}_{1}\left(\cdot ,t\right)-{\overset{`}{v}}_{1}\left(\cdot ,t\right)=e^{tA_{1}}\left({\overset{´}{v}}_{1}^{0}-{\overset{`}{v}}_{1}^{0}\right)+\int_{0}^{t}e^{\left(t-s\right)A_{1}}\left({\overset{´}{v}}_{1}\left(\cdot ,s\right){\overset{´}{v}}_{2}\left(\cdot ,s\right)-{\overset{`}{v}}_{1}\left(\cdot ,s\right){\overset{`}{v}}_{2}\left(\cdot ,s\right)\right)ds\\ \; & -a\int_{0}^{t}e^{\left(t-s\right)A_{1}}\left({\overset{´}{v}}_{1}\left(\cdot ,s\right)-{\overset{`}{v}}_{1}\left(\cdot ,s\right)\right)ds+\int_{0}^{t}e^{\left(t-s\right)A_{1}}\left({\overset{´}{v}}_{3}\left(\cdot ,s\right)-{\overset{`}{v}}_{3}\left(\cdot ,s\right)\right)ds\\ \; & +\int_{0}^{t}e^{\left(t-s\right)A_{1}}\left({\overset{´}{v}}_{4}\left(\cdot ,s\right)-{\overset{`}{v}}_{4}\left(\cdot ,s\right)\right)ds. \end{array}$$

By applying Lemmas 1 and 2, we have

$$\begin{array}{cc} \; & ∥\int_{0}^{t}e^{\left(t-s\right)A_{1}}\left({\overset{´}{v}}_{1}\left(\cdot ,s\right){\overset{´}{v}}_{2}\left(\cdot ,s\right)-{\overset{`}{v}}_{1}\left(\cdot ,s\right){\overset{`}{v}}_{2}\left(\cdot ,s\right)\right){ds∥}_{L^{2}\left(\Omega \right)}\\ =\; & \frac{1}{2}∥\int_{0}^{t}e^{\left(t-s\right)A_{1}}(\left({\overset{´}{v}}_{1}\left(\cdot ,s\right)-{\overset{`}{v}}_{1}\left(\cdot ,s\right)\right)\left({\overset{´}{v}}_{2}\left(\cdot ,s\right)+{\overset{`}{v}}_{2}\left(\cdot ,s\right)\right)\\ \; & +\left({\overset{´}{v}}_{1}\left(\cdot ,s\right)+{\overset{`}{v}}_{1}\left(\cdot ,s\right)\right)\left({\overset{´}{v}}_{2}\left(\cdot ,s\right)-{\overset{`}{v}}_{2}\left(\cdot ,s\right)\right){)ds∥}_{L^{2}\left(\Omega \right)}\\ ⩽\; & \frac{M_{1}}{2}\int_{0}^{t}{\left(t-s\right)}^{-\frac{N}{4}}∥\left({\overset{´}{v}}_{1}\left(\cdot ,s\right)-{\overset{`}{v}}_{1}\left(\cdot ,s\right)\right)\left({\overset{´}{v}}_{2}\left(\cdot ,s\right)+{\overset{`}{v}}_{2}\left(\cdot ,s\right)\right)\\ \; & +\left({\overset{´}{v}}_{1}\left(\cdot ,s\right)+{\overset{`}{v}}_{1}\left(\cdot ,s\right)\right)\left({\overset{´}{v}}_{2}\left(\cdot ,s\right)-{\overset{`}{v}}_{2}\left(\cdot ,s\right)\right){∥}_{L^{1}\left(\Omega \right)}ds\\ ⩽\; & \frac{M_{1}}{2}\int_{0}^{t}{\left(t-s\right)}^{-\frac{N}{4}}(∥{\overset{´}{v}}_{1}\left(\cdot ,s\right)-{\overset{`}{v}}_{1}{\left(\cdot ,s\right)∥}_{L^{2}\left(\Omega \right)}{∥{\overset{´}{v}}_{2}\left(\cdot ,s\right)+{\overset{`}{v}}_{2}\left(\cdot ,s\right)∥}_{L^{2}\left(\Omega \right)}\\ \; & +∥{\overset{´}{v}}_{1}\left(\cdot ,s\right)+{\overset{`}{v}}_{1}{\left(\cdot ,s\right)∥}_{L^{2}\left(\Omega \right)}{∥{\overset{´}{v}}_{2}\left(\cdot ,s\right)-{\overset{`}{v}}_{2}\left(\cdot ,s\right)∥}_{L^{2}\left(\Omega \right)})ds. \end{array}$$

This, together with (A9), implies immediately

(A10) $$\begin{array}{cc} \; & ∥{\overset{´}{v}}_{1}\left(\cdot ,t\right)-{\overset{`}{v}}_{1}{\left(\cdot ,t\right)∥}_{L^{2}\left(\Omega \right)}⩽{∥e^{tA_{1}}\left({\overset{´}{v}}_{1}^{0}-{\overset{`}{v}}_{1}^{0}\right)∥}_{L^{2}\left(\Omega \right)}\\ \; & +∥\int_{0}^{t}e^{\left(t-s\right)A_{1}}\left({\overset{´}{v}}_{1}\left(\cdot ,s\right){\overset{´}{v}}_{2}\left(\cdot ,s\right)-{\overset{`}{v}}_{1}\left(\cdot ,s\right){\overset{`}{v}}_{2}\left(\cdot ,s\right)\right){ds∥}_{L^{2}\left(\Omega \right)}\\ \; & +a∥\int_{0}^{t}e^{\left(t-s\right)A_{1}}\left({\overset{´}{v}}_{1}\left(\cdot ,s\right)-{\overset{`}{v}}_{1}\left(\cdot ,s\right)\right){ds∥}_{L^{2}\left(\Omega \right)}\\ \; & +∥\int_{0}^{t}e^{\left(t-s\right)A_{1}}\left({\overset{´}{v}}_{3}\left(\cdot ,s\right)-{\overset{`}{v}}_{3}\left(\cdot ,s\right)\right){ds∥}_{L^{2}\left(\Omega \right)}\\ \; & +∥\int_{0}^{t}e^{\left(t-s\right)A_{1}}\left({\overset{´}{v}}_{4}\left(\cdot ,s\right)-{\overset{`}{v}}_{4}\left(\cdot ,s\right)\right){ds∥}_{L^{2}\left(\Omega \right)}\\ \; & ⩽∥{\overset{´}{v}}_{1}^{0}-{\overset{`}{v}}_{1}^{0}{∥}_{L^{2}\left(\Omega \right)}+a\int_{0}^{t}{∥{\overset{´}{v}}_{1}\left(\cdot ,s\right)-{\overset{`}{v}}_{1}\left(\cdot ,s\right)∥}_{L^{2}\left(\Omega \right)}ds\\ \; & +\frac{M_{1}}{2}\int_{0}^{t}{\left(t-s\right)}^{-\frac{N}{4}}(∥{\overset{´}{v}}_{1}\left(\cdot ,s\right)-{\overset{`}{v}}_{1}{\left(\cdot ,s\right)∥}_{L^{2}\left(\Omega \right)}{∥{\overset{´}{v}}_{2}\left(\cdot ,s\right)+{\overset{`}{v}}_{2}\left(\cdot ,s\right)∥}_{L^{2}\left(\Omega \right)}\\ \; & +∥{\overset{´}{v}}_{1}\left(\cdot ,s\right)+{\overset{`}{v}}_{1}{\left(\cdot ,s\right)∥}_{L^{2}\left(\Omega \right)}{∥{\overset{´}{v}}_{2}\left(\cdot ,s\right)-{\overset{`}{v}}_{2}\left(\cdot ,s\right)∥}_{L^{2}\left(\Omega \right)})ds\\ \; & +\int_{0}^{t}∥{\overset{´}{v}}_{3}\left(\cdot ,s\right)-{\overset{`}{v}}_{3}{\left(\cdot ,s\right)∥}_{L^{2}\left(\Omega \right)}ds+\int_{0}^{t}{∥{\overset{´}{v}}_{4}\left(\cdot ,s\right)-{\overset{`}{v}}_{4}\left(\cdot ,s\right)∥}_{L^{2}\left(\Omega \right)}ds. \end{array}$$

By mimicking steps in (A10), we deduce from (A2) and (A6) that

(A11) $$\begin{array}{cc} \; & ∥{\overset{´}{v}}_{2}\left(\cdot ,t\right)-{\overset{`}{v}}_{2}{\left(\cdot ,t\right)∥}_{L^{2}\left(\Omega \right)}⩽∥{\overset{´}{v}}_{2}^{0}-{\overset{`}{v}}_{2}^{0}{∥}_{L^{2}\left(\Omega \right)}+b\int_{0}^{t}{∥{\overset{´}{v}}_{2}\left(\cdot ,s\right)-{\overset{`}{v}}_{2}\left(\cdot ,s\right)∥}_{L^{2}\left(\Omega \right)}ds\\ \; & +M_{1}\int_{0}^{t}{\left(t-s\right)}^{-\frac{N}{4}}∥{\overset{´}{v}}_{1}\left(\cdot ,s\right)+{\overset{`}{v}}_{1}{\left(\cdot ,s\right)∥}_{L^{2}\left(\Omega \right)}{∥{\overset{´}{v}}_{1}\left(\cdot ,s\right)-{\overset{`}{v}}_{1}\left(\cdot ,s\right)∥}_{L^{2}\left(\Omega \right)}ds. \end{array}$$

By mimicking steps in (A10) and (A11), we deduce from (A3) and (A7) that

(A12) $$\begin{array}{cc} \; & ∥{\overset{´}{v}}_{3}\left(\cdot ,t\right)-{\overset{`}{v}}_{3}{\left(\cdot ,t\right)∥}_{L^{2}\left(\Omega \right)}⩽∥{\overset{´}{v}}_{3}^{0}-{\overset{`}{v}}_{3}^{0}{∥}_{L^{2}\left(\Omega \right)}+\int_{0}^{t}{∥{\overset{´}{v}}_{1}\left(\cdot ,s\right)-{\overset{`}{v}}_{1}\left(\cdot ,s\right)∥}_{L^{2}\left(\Omega \right)}ds\\ \; & +c\int_{0}^{t}{∥{\overset{´}{v}}_{3}\left(\cdot ,s\right)-{\overset{`}{v}}_{3}\left(\cdot ,s\right)∥}_{L^{2}\left(\Omega \right)}ds. \end{array}$$

By mimicking steps in (A10)–(A12), we deduce from (A4) and (A8) that

(A13) $$\begin{array}{cc} \; & ∥{\overset{´}{v}}_{4}\left(\cdot ,t\right)-{\overset{`}{v}}_{4}{\left(\cdot ,t\right)∥}_{L^{2}\left(\Omega \right)}⩽∥{\overset{´}{v}}_{4}^{0}-{\overset{`}{v}}_{4}^{0}{∥}_{L^{2}\left(\Omega \right)}+\alpha \int_{0}^{t}{∥{\overset{´}{v}}_{4}\left(\cdot ,s\right)-{\overset{`}{v}}_{4}\left(\cdot ,s\right)∥}_{L^{2}\left(\Omega \right)}ds\\ \; & +\frac{M_{1}\beta }{2}\int_{0}^{t}{\left(t-s\right)}^{-\frac{N}{4}}(∥{\overset{´}{v}}_{1}\left(\cdot ,s\right)-{\overset{`}{v}}_{1}{\left(\cdot ,s\right)∥}_{L^{2}\left(\Omega \right)}{∥{\overset{´}{v}}_{2}\left(\cdot ,s\right)+{\overset{`}{v}}_{2}\left(\cdot ,s\right)∥}_{L^{2}\left(\Omega \right)}\\ \; & +∥{\overset{´}{v}}_{1}\left(\cdot ,s\right)+{\overset{`}{v}}_{1}{\left(\cdot ,s\right)∥}_{L^{2}\left(\Omega \right)}{∥{\overset{´}{v}}_{2}\left(\cdot ,s\right)-{\overset{`}{v}}_{2}\left(\cdot ,s\right)∥}_{L^{2}\left(\Omega \right)})ds. \end{array}$$

For the convenience of our later presentation, we denote

(A14) $$\begin{array}{cccc} \; & s\left(t\right)=\sum_{k=1}^{4}{∥{\overset{´}{v}}_{k}\left(\cdot ,t\right)-{\overset{`}{v}}_{k}\left(\cdot ,t\right)∥}_{L^{2}\left(\Omega \right)} & \; & \mathrm{for}\;t\in [0,T], \end{array}$$

from which it follows that

(A15) $$s\left(0\right)=\sum_{k=1}^{4}{∥{\overset{´}{v}}_{k}^{0}-{\overset{`}{v}}_{k}^{0}∥}_{L^{2}\left(\Omega \right)}.$$

By using the celebrated GM-AM inequality, we can prove

$$\begin{array}{cc} \; & \sum_{k=1}^{4}\left(∥{\overset{´}{v}}_{k}{\left(\cdot ,t\right)∥}_{L^{2}\left(\Omega \right)}+{∥{\overset{`}{v}}_{k}\left(\cdot ,t\right)∥}_{L^{2}\left(\Omega \right)}\right)\\ ⩽\; & 2+\sum_{k=1}^{4}∥{\overset{´}{v}}_{k}{\left(\cdot ,t\right)∥}_{L^{2}\left(\Omega \right)}^{2}+\sum_{k=1}^{4}{∥{\overset{`}{v}}_{k}\left(\cdot ,t\right)∥}_{L^{2}\left(\Omega \right)}^{2},\;t\in \left[0,T\right]. \end{array}$$

However, in view of (43) and (44) in the proof of Theorem 1 (see also Theorem 2), we find

$$\begin{array}{cccc} \; & \sum_{k=1}^{4}∥{\overset{´}{v}}_{k}{\left(\cdot ,t\right)∥}_{L^{2}\left(\Omega \right)}^{2}⩽M_{8}\sum_{k=1}^{4}{∥{\overset{´}{v}}_{k}^{0}∥}_{L^{2}\left(\Omega \right)}^{2}e^{M_{9}t}\; & \; & \mathrm{for}\;t\in [0,T],\\ \; & \sum_{k=1}^{4}∥{\overset{`}{v}}_{k}{\left(\cdot ,t\right)∥}_{L^{2}\left(\Omega \right)}^{2}⩽M_{8}\sum_{k=1}^{4}{∥{\overset{`}{v}}_{k}^{0}∥}_{L^{2}\left(\Omega \right)}^{2}e^{M_{9}t}\; & \; & \mathrm{for}\;t\in [0,T], \end{array}$$

where the positive constants, $M_{8}$ and $M_{9}$, given exactly as in (47) in Theorem 2, depend merely on *a*, *b*, *c*, α, β, Ω, $D_{k}$ (k=1,2,3,4). Based on (A10)–(A13), we have, by utilizing some calculations,

(A16) $$\begin{array}{cc} \; & s\left(t\right)⩽s\left(0\right)+\max\left(a+1,b,c+1,\alpha +1\right)\int_{0}^{t}s\left(s\right)ds\\ \; & +\frac{M_{1}\left(\max(\beta ,1)+1\right)}{2}\int_{0}^{t}{\left(t-s\right)}^{-\frac{N}{4}}\sum_{k=1}^{2}\left(∥{\overset{´}{v}}_{k}{\left(\cdot ,s\right)∥}_{L^{2}\left(\Omega \right)}+{∥{\overset{`}{v}}_{k}\left(\cdot ,s\right)∥}_{L^{2}\left(\Omega \right)}\right)s\left(s\right)ds\\ \; & ⩽s\left(0\right)+r\left(t\right)\int_{0}^{t}{\left(t-s\right)}^{-\frac{N}{4}}s\left(s\right)ds,\;t\in \left[0,T\right], \end{array}$$

where the function r(t) is given by

$$\begin{array}{cc} \; & r\left(t\right)=t^{\frac{N}{4}}\max\left(a+1,b,c+1,\alpha +1\right)\\ \; & +\frac{M_{1}\left(\max(\beta ,1)+1\right)}{2}\left(2+M_{8}(\sum_{k=1}^{4}∥{\overset{´}{v}}_{k}^{0}{∥}_{L^{2}\left(\Omega \right)}^{2}+\sum_{k=1}^{4}∥{\overset{`}{v}}_{k}^{0}{∥}_{L^{2}\left(\Omega \right)}^{2})e^{M_{9}t}\right),\;t\in \left[0,T\right]. \end{array}$$

Thanks to the observation that the function r(t) is strictly increasing in [0,T] and that the function $E_{\frac{4-N}{4}}\left(z\right)$ is strictly increasing in $R^{+}$, by Lemma A1, we deduce from (A16) that

$$\begin{array}{cc} s(t)⩽\; & s\left(0\right)E_{\frac{4-N}{4}}\left(\Gamma \left(\frac{4-N}{4}\right)r\left(t\right)t^{\frac{4-N}{4}}\right)\\ ⩽\; & s\left(0\right)E_{\frac{4-N}{4}}\left(\Gamma \left(\frac{4-N}{4}\right)r\left(T\right)T^{\frac{4-N}{4}}\right),\;t\in \left[0,T\right]. \end{array}$$

This, together with (A14) and (A15), implies

$$\begin{array}{c} \underset{t\in [0,T]}{\sup}\sum_{k=1}^{4}∥{\overset{´}{v}}_{k}\left(\cdot ,t\right)-{\overset{`}{v}}_{k}{\left(\cdot ,t\right)∥}_{L^{2}\left(\Omega \right)}⩽E_{\frac{4-N}{4}}\left(\Gamma \left(\frac{4-N}{4}\right)r\left(T\right)T^{\frac{4-N}{4}}\right)\sum_{k=1}^{4}{∥{\overset{´}{v}}_{k}^{0}-{\overset{`}{v}}_{k}^{0}∥}_{L^{2}\left(\Omega \right)}. \end{array}$$

This, together with Theorem 2, implies that for every T∈(0,+∞), the data-to-solution map

$$L^{2}\left(\Omega ;R^{4}\right)∋{\left(v_{1}^{0},v_{2}^{0},v_{3}^{0},v_{4}^{0}\right)}^{⊤}\mapsto {\left(v_{1},v_{2},v_{3},v_{4}\right)}^{⊤}\in C\left(\left[0,T\right];L^{2}\left(\Omega ;R^{4}\right)\right)$$

is locally Lipschitz continuous. Lastly, it is worth mentioning that it is not difficult to find that the uniqueness follows directly from the local Lispchitz continuity of the data-to-solution map. □
